# Progress in adolescent health and wellbeing: tracking 12 headline indicators for 195 countries and territories, 1990–2016

**DOI:** 10.1016/S0140-6736(18)32427-9

**Published:** 2019-03-16

**Authors:** Peter S Azzopardi, Stephen J C Hearps, Kate L Francis, Elissa C Kennedy, Ali H Mokdad, Nicholas J Kassebaum, Stephen Lim, Caleb M S Irvine, Theo Vos, Alex D Brown, Surabhi Dogra, Stuart A Kinner, Natasha S Kaoma, Mariam Naguib, Nicola J Reavley, Jennifer Requejo, John S Santelli, Susan M Sawyer, Vegard Skirbekk, Marleen Temmerman, Jordan Tewhaiti-Smith, Joseph L Ward, Russell M Viner, George C Patton

**Affiliations:** a Murdoch Children's Research Institute, Melbourne, VIC, Australia; b Maternal and Child Health Program, Burnet Institute, Melbourne, VIC, Australia; c Wardliparingga Aboriginal Research Unit, South Australian Health and Medical Research Institute, Adelaide, SA, Australia; d Department of Paediatrics, The University of Melbourne, Parkville, VIC, Australia; e Melbourne School of Population and Global Health, The University of Melbourne, Parkville, VIC, Australia; f Centre for Adolescent Health, Royal Children's Hospital, Parkville, VIC, Australia; g Department of Epidemiology and Preventive Medicine, Monash University, Melbourne, VIC, Australia; h Institute for Health Metrics and Evaluation, University of Washington, Seattle, WA, USA; i Department of Anesthesiology and Pain Medicine, University of Washington, Seattle, WA, USA; j Faculty of Health and Medical Science, University of Adelaide, Adelaide, SA, Australia; k Youth Commissioner, Lancet Standing Commission on Adolescent Health and Wellbeing, Lusaka, Zambia; l Copper Rose, Lusaka, Zambia; m United Nations Children's Fund, New York, NY, USA; n Institute for International Programs, Johns Hopkins Bloomberg School of Public Health, Baltimore, MD, USA; o Mailman School of Public Health, Columbia University, New York, NY, USA; p Department of Population and Family Health at the Mailman School, Columbia University, New York, NY, USA; q Centre for Fertility and Health, Norwegian Institute of Public Health, Nydalen, Oslo, Norway; r Centre of Excellence in Women, Child and Adolescent Health, Aga Khan University, Nairobi, Kenya; s Faculty of Medicine and Health Sciences, Ghent University, Ghent, Belgium; t UCL Institute of Child Health, University College London, London, UK

## Abstract

**Background:**

Rapid demographic, epidemiological, and nutritional transitons have brought a pressing need to track progress in adolescent health. Here, we present country-level estimates of 12 headline indicators from the *Lancet* Commission on adolescent health and wellbeing, from 1990 to 2016.

**Methods:**

Indicators included those of health outcomes (disability-adjusted life-years [DALYs] due to communicable, maternal, and nutritional diseases; injuries; and non-communicable diseases); health risks (tobacco smoking, binge drinking, overweight, and anaemia); and social determinants of health (adolescent fertility; completion of secondary education; not in education, employment, or training [NEET]; child marriage; and demand for contraception satisfied with modern methods). We drew data from the Global Burden of Diseases, Injuries, and Risk Factors Study (GBD) 2016, International Labour Organisation, household surveys, and the Barro-Lee education dataset.

**Findings:**

From 1990 to 2016, remarkable shifts in adolescent health occurred. A decrease in disease burden in many countries has been offset by population growth in countries with the poorest adolescent health profiles. Compared with 1990, an additional 250 million adolescents were living in multi-burden countries in 2016, where they face a heavy and complex burden of disease. The rapidity of nutritional transition is evident from the 324·1 million (18%) of 1·8 billion adolescents globally who were overweight or obese in 2016, an increase of 176·9 million compared with 1990, and the 430·7 million (24%) who had anaemia in 2016, an increase of 74·2 million compared with 1990. Child marriage remains common, with an estimated 66 million women aged 20–24 years married before age 18 years. Although gender-parity in secondary school completion exists globally, prevalence of NEET remains high for young women in multi-burden countries, suggesting few opportunities to enter the workforce in these settings.

**Interpretation:**

Although disease burden has fallen in many settings, demographic shifts have heightened global inequalities. Global disease burden has changed little since 1990 and the prevalence of many adolescent health risks have increased. Health, education, and legal systems have not kept pace with shifting adolescent needs and demographic changes. Gender inequity remains a powerful driver of poor adolescent health in many countries.

**Funding:**

Australian National Health and Medical Research Council, and the Bill & Melinda Gates Foundation.

## Introduction

Adolescence is a formative phase of life during which patterns of growth, development, and behaviour lay a foundation for health in later life and for the next generation.^1^, ^2^ The importance of adolescent health has been further heightened by the population of people aged 10–24 years being the largest in history, at 1·8 billion in 2016.^3^ In response, in 2015 the United Nations (UN) extended the existing Every Woman, Every Child agenda to include adolescents through the Global Strategy for Women's, Children's, and Adolescents' Health.^4^, ^5^, ^6^

The Global Strategy highlighted a need for sound health data to drive accountability.^1^, ^7^ Adolescents were mentioned in 12 of 232 Sustainable Development Goals (SDGs) indicators relevant to health, including indicators associated with nutrition, reproductive health, sexual and intimate partner violence, child marriage, education, and employment.^8^ The 60 indicators for the Global Strategy overlapped with those of the 12 SDGs and included adolescent mortality and fertility as adolescent-specific indicators.^9^, ^10^ The inclusion of adolescents in Countdown to 2030,^11^ in which focus had previously been exclusively on maternal and child health, marked a further step towards a system for tracking adolescent health. However, in these and other initiatives designed to monitor progress in adolescent health and wellbeing (eg, the United Nations [UN] International Children's Emergency Fund's [UNICEF] proposed National Adolescent Assessment Cards^12^), the lack of good quality and timely data has remained a barrier to progress.^13^ Failure to include all adolescents in routine data collections has led to gaps, particularly for younger adolescents (aged 10–14 years), males, and those out of school.^1^, ^14^ Additionally, many aspects of adolescent health and health risk, including mental and substance use disorders and sexual violence and abuse remain poorly covered in existing global surveys.^7^, ^15^, ^16^

**Research in context**

**Evidence before this study**

A lack of well defined indicators that can be readily populated with data has been a barrier to progress and investment in adolescent health and wellbeing. We reviewed existing indicator frameworks as part of the 2016 *Lancet* Commission on adolescent health and wellbeing, and then as part of a technical working paper for United Nations (UN) International Children's Emergency Fund's (UNICEF) in 2017. We reviewed the Sustainable Development Goals; the Global Strategy of Women's Children's and Adolescent's Health; UNICEF's proposed adolescent health tracker; Countdown to 2030; a mapping of adolescent health indicators in a *Lancet* series on adolescent health; and earlier work by WHO to define core indicators of adolescent health. Indicator frameworks published before the Commission have had little comparability, captured restricted aspects of adolescent health, and generally mapped poorly to available data. The Commission proposed 12 headline indicators that mapped to a conceptual model for adolescent health and wellbeing that encompassed major health needs, health risks, and social determinants of health. The headline indicators were designed to capture shifting patterns of adolescent health with progress through the epidemiological transition and availability through existing data sources.

**Added value of this study**

To our knowledge, this is the first comprehensive and integrated overview of recent global shifts in adolescent health at a global level, extending to country-level estimates for 195 countries and territories. We outline the shifts in adolescent health needs that have taken place between 1990 and 2016 in the context of rapid epidemiological and nutritional transitions and major demographic changes. We describe trends in health risks and major social determinants of health. We also report trends over time for each indicator since 1990 to identify in which countries the least progress has been made and where health needs are rapidly emerging.

**Implications of all the available evidence**

A pressing need exists for adoption of comprehensive and integrated approaches to adolescent health and monitoring the success of these investments. Disease burden has decreased in many countries, but demographic shifts mean many more adolescents face major health problems. Geographical inequality has increased with a shift in disease burden to countries that have the lowest resources and the largest adolescent populations. Compared with 1990, an additional 250 million adolescents lived in multi-burden countries in 2016, where they faced a triple burden of health problems extending from infectious diseases and other group 1 causes of disease burden, to high prevalences of injury, violence, and non-communicable diseases including mental disorders. Nutritional health risks have become more prominent with a 120% increase in the prevalence of adolescent overweight or obesity. Furthermore, the absolute number of adolescents living with anaemia was 20% higher in 2016 than in 1990. Indicators reflecting the capacity of health, educational, employment, and legal systems suggest that they have not kept pace with rapid demographic and geographical shifts in adolescent needs. To ensure targeted and accountable action, the global community must now agree on a minimum set of well defined indicators that are specific to adolescent health and that can be readily populated with data; the indicators as reported here could provide an important foundation for this task.

The 2016 *Lancet* Commission on adolescent health and wellbeing^1^ proposed a set of 12 headline indicators as an interim mechanism for tracking progress in adolescent health. The indicators were defined for their comparatively high data coverage and quality. They captured disease burden, health risks, and prominent social determinants of health during the adolescent years.^1^, ^17^ We aimed to populate these indicators with data for 195 countries and territories from 1990 to 2016. We additionally aimed to aggregate these estimates for each country and group the countries into three disease-burden groups that were laid out by the Commission, and to delineate shifting priorities with progression through the epidemiological transition.^1^

## Methods

### Data sources and definitions

We populated the 12 headline indicators for adolescent health as defined by the *Lancet* Commission on adolescent health and wellbeing (table 1),^1^ hereafter referred to as the Commission, with global, country-level, and disease-group data. In selecting data sources, we considered coverage, data quality, international comparability, and recency of data. We first reviewed relevant data available at the Institute of Health Metrics and Evaluation (IHME). Although some data might be available elsewhere, IHME has extensively catalogued primary health data for each country across the world. For their Global Burden of Diseases, Injuries, and Risk Factors Study (GBD), they have harmonised estimates and used models to fill data gaps to produce estimates that are updated annually for 333 health outcomes and 84 risks and determinants by country or territory, sex, and 5-year age group.^18^, ^19^ We used data from GBD 2016, which included 195 countries and territories. Specific methods and definitions of GBD 2016 are described elsewhere.^18^, ^19^, ^20^, ^21^ Data for some indicators of social determinants were not readily available at IHME and we reviewed other relevant global data collections to obtain these.^7^, ^13^

**Table 1:** Definitions and data availability for 12 headline indicators of adolescent health from the *Lancet* Commission on adolescent health and wellbeing

	**Data source**	**Countries covered (n=195)**	**Definition**	**Short title**
**Health outcome**				
DALYs due to communicable, maternal, and nutritional diseases in individuals aged 10–24 years	IHME	195 (100%)	DALYs per 100 000 adolescents due to communicable, maternal, and nutritional diseases in individuals aged 10–24 years	Group 1 DALYs
DALYs due to injury and violence in individuals aged 10–24 years	IHME	195 (100%)	DALYs per 100 000 adolescents due to injury and violence in individuals aged 10–24 years	Injury DALYs
DALYs due to non-communicable diseases in individuals aged 10–24 years	IHME	195 (100%)	DALYs per 100 000 adolescents due to non-communicable diseases in individuals aged 10–24 years	Non-communicable disease DALYs
**Health risks**				
Daily smoking in individuals aged 10–24 years	IHME	195 (100%)	Prevalence of use of any smoked tobacco product in individuals aged 10–24 years	Daily tobacco
Binge drinking in past 12 months in individuals aged 15–19 years	IHME	195 (100%)	Prevalence of binge alcohol use (>48 g of alcohol for females, >60 g for males) in the past 12 months for individuals aged 15–19 years	Binge drinking
Individuals aged 10–24 years who exceed WHO guidelines for overweight	IHME	195 (100%)	Prevalence of overweight and obesity (IOTF thresholds, age-specific and sex-specific thresholds, equivalent to a BMI ≥25 kg/m^2^ at age 18 years) in individuals aged 10–24 years	Overweight and obesity
Prevalence of iron deficiency anaemia in individuals aged 10–24 years	IHME	195 (100%)	Prevalence of anaemia in individuals aged 10–24 years: for those aged 10–14 years haemoglobin <115 g/L; for those aged 15–24 years, <130 g/L for males, <120 g/L for non-pregnant females, and <110 g/L for pregnant females	Anaemia
**Social determinants of health**				
Completing ≥12 years of education among individuals aged 20–24 years	Barro-Lee	143 (93%)	Proportion completing secondary school among individuals aged 20–24 years	Secondary education
Individuals aged 20–24 years who are NEET	International Labour Organization	123 (75%)	Proportion of individuals aged 15–24 years not in employment, education, or training	NEET
Annual birth rate per 1000 adolescents aged 10–19 years	IHME	195 (100%)	Birth rate (livebirths per 1000 population per year) in females aged 15–19 years	Adolescent livebirths
Marriage before age 18 years in women aged 20–24 years	Multiple Indicator Cluster Survey and Demographic and Health Survey	120 (62%)	Proportion of females aged 20–24 years in marriage or union before age 18 years	Child marriage
Females aged 15–24 years with met need for contraception	IHME	195 (100%)	Proportion of females aged 15–24 years whose demand for contraception is satisfied with a modern method	Demand for modern contraception satisfied

DALYs=disability-adjusted life-years. IHME=Institute for Health Metrics and Evaluation. IOTF=World Obesity Federation. BMI=body-mass index. NEET=not in education, employment, or training.

In the Commission, the indicators were organised into three main categories: health outcomes, health risks, and social determinants of health. Brief definitions of each indicator and the data sources used are shown in table 1 (full definitions of each indicator and data sources are in the appendix). Indicators were generally defined for adolescents aged 10–24 years. This age definition reliably captures developmental stage during biological, social, and neurocognitive transitions.^3^

We reported indicators for adolescents in 195 locations, as defined by IHME, comprising 188 countries (as classified by the UN) and seven territories (Puerto Rico, American Samoa, Bermuda, Greenland, Guam, Northern Mariana Islands, and the Virgin Islands). Hereafter, countries refers to both UN countries and territories. Because the population of these 195 countries is more than 99·9% of the global population, in this analyses we report our data as global estimates.

The Commission defined three country disease groups (country groups) on the basis of adolescent disease burden to represent different stages of the epidemiological transition (and therefore different patterns of health need) for adolescents.^1^

Countries were defined as multi-burden if adolescents (aged 10–24 years, both sexes combined) of that country had a burden of communicable, maternal, and nutritional conditions (ie, group 1 conditions; table 1) of 2500 disability-adjusted life-years (DALYs) or more per 100 000 adolescents. This threshold was set using GBD 2013 data to capture countries where DALYs caused by group 1 conditions in 2013 were at least double the average rate for countries where non-communicable diseases were predominant, and countries where group 1 conditions accounted for at least 20% of the total burden among those aged 10–24 years.

Countries were defined as injury excess if adolescents were estimated to have 2500 DALYs or more per 100 000 adolescents due to injury and less than 2500 DALYs per 100 000 population due to group 1 conditions. This threshold was set to identify those countries that had a low burden of group 1 conditions but that had a burden of injuries at least twice that of countries where non-communicable diseases were predominant, and countries where injury accounted for least 20% of the total burden for adolescents.

Countries were defined as non-communicable disease predominant if both injuries and group 1 conditions each contributed less than 2500 DALYs per 100 000 adolescents to the disease burden. We used these definitions to define country groups in 1990 and 2016; country groupings reported are for 2016 unless otherwise specified. Hence, with progression through the epidemiological transition, a country would be expected to move up from the multi-burden group to the injury excess group, and from the injury-excess group to the non-communicable disease-predominant group. These country groupings broadly corresponded to socioeconomic development (defined in the appendix). The median Socio-demographic Index (SDI) value for multi-burden countries was 0·45 (range 0·19–0·74), which was generally lower than injury-excess countries (SDI median 0·71, range 0·47–0·88) and the non-communicable disease-predominant countries (SDI median 0·8, range 0·45–0·94). The country groupings also broadly corresponded with World Bank income levels, with almost all (30 [97%] of 31) low-income countries in 2016 classified as multi-burden and almost all (52 [88%] of 59) high-income countries classified as non-communicable disease predominant (appendix).

### Analysis and reporting

We report the most recent estimate for each indicator at a country level. For each indicator we identified the countries with the lowest and highest observed values and ranked each country in between, presenting this ranking as a heat map. We also report estimates for each indicator for the three country groups and globally. We report group estimates using country-level estimates for 2016 for indicators drawn from IHME, 2010 estimates for secondary education, and the most recent country-specific data available for child marriage (2003–16), and not in education, employment, or training (NEET; 2005–16; appendix). For indicators drawn from IHME data, we generated group counts as the sum of counts in each country. We then used these group counts of numerator and denominator to generate estimates of group prevalence. For the indicators of secondary education, child marriage, and NEET, the group prevalence estimates reflect only countries for which data were available, and we calculated the count estimates (estimated number of adolescents) using the prevalence derived from the available data and applied that to the total denominator population of that group. For the indicator of child marriage, data were not available for many countries in the non-communicable disease-predominant group and data were mostly missing for high-income countries. To estimate the count and prevalence of child marriage in the non-communicable disease-predominant country group, we assumed that non-communicable disease-predominant countries without an estimate had a prevalence equivalent to the lowest prevalence observed in the rest of the group. Data for child marriage were of sufficient coverage for injury-excess and multi-burden countries. In addition to estimating the group counts on the basis of observed data, we estimated global counts from two scenarios to distinguish changes between 1990 and 2016 due to shifts in health and due to population change—ie, stable demography and changing epidemiology, and changing demography with fixed epidemiology.

We populated indicators associated with NEET and child marriage from primary data; however, standard errors (and therefore confidence intervals) were not readily available for these estimates. We drew data for estimates for the other indicators from modelled data based on multiple individual primary data sources. Uncertainty estimates for these estimates (distinct from confidence intervals in that they represent uncertainty derived from primary data, model estimation, and model specification) were available at a country level for adolescents but were not readily available for manipulated data (including aggregate country groupings). Uncertainty estimates at a country level for indicators drawn from IHME (other than adolescent fertility) are provided in the appendix. Adolescent fertility in GBD 2016 was modelled using a hybrid approach of modelling the total fertility rate directly and then fitting UN World Population Prospects age patterns of fertility to those estimates; uncertainty estimates for adolescent fertility were not available. Uncertainty estimates were not available for indicators of injury and non-communicable disease burden because of the redistribution of self-harm from the IHME level 1 injury group to the level 1 non-communicable disease group because of its association with mental disorders. To enable assessment of data quality, we provide uncertainty estimates for the IHME level 1 groups and self-harm separately. Furthermore, we provide uncertainty estimates for all-cause years of life lost (YLL) and years lost due to disability (YLD) for adolescents to allow an assessment of the quality of data of the two broad components of DALYs.

We report annual rates of change for each indicator at a country and group level. Linear regression models were fitted to available data points for each indicator and location, and we used the β coefficient to estimate the annual rate of change (expressed as percentage change). We used linear regression (rather than the estimates at each extreme of the time series) to account for fluctuations in some indicators over time (eg, an increase in DALYs due to war or conflict). We used data points for 1990–2015 in intervals of 5 years plus 2016 for data drawn from IHME. For data drawn from Barro-Lee, an educational attainment dataset that covers the period 1950–2010, we used intervals of 5 years for data from the period 1990–2010. Rates of change for NEET and child marriage could not be caulated because of restricted data over time.

### Role of the funding source

The funders provided salaries for research staff and had no role in study design, data collection, data analysis, data interpretation, or writing of the report. PSA, SJCH, KLF, ECK, and GCP had access to all the data; AHM, NJK, SL, CMSI, and TV had access to all the data as provided by IHME; and all other authors able to access the data as requested. The corresponding author had final responsibility for the decision to submit for publication.

## Results

### Global trends in adolescent health and demography

**Figure 1:**
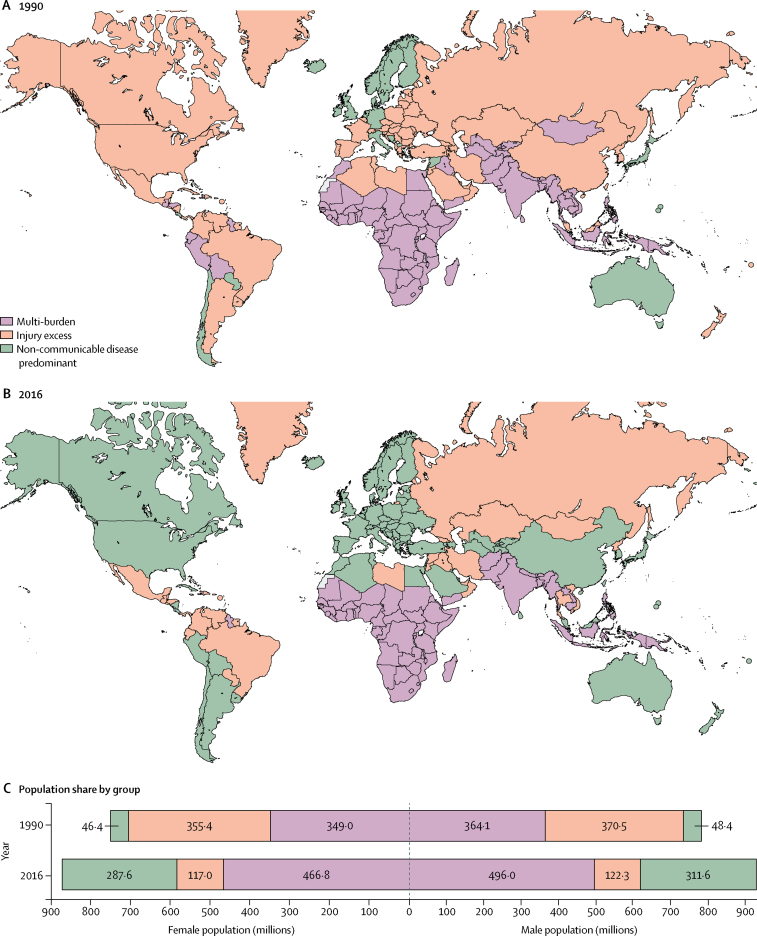
Adolescent health country groupings in 1990 (A) and 2016 (B) with population distribution of adolescents in the three groups at both timepoints, by sex (C)

In 1990, 90 (46%) of 195 countries were classified as multi-burden, which included most countries in sub-Saharan Africa; central, south, and east Asia; and several countries in Latin America, Oceania, the Middle East, and north Africa (figure 1). 73 (38%) countries were classified as injury excess in 1990, mostly in eastern Europe and Latin America, but also several countries in western Europe (including France, Spain, Switzerland), the Middle East, and the USA. The remaining 32 (16%) countries were classified as non-communicable disease predominant.

Between 1990 and 2016, 73 (37%) countries shifted their disease group, all of which progressed through the epidemiological transition, except for Dominica, Jamaica, Paraguay, and Syria, which moved from the non-communicable disease-predominant group to the injury-excess group (figure 1). No country moved back to multi-burden. By 2016, many countries in central and east Asia, western Europe, the Middle East, and the Americas had made a transition to the non-communicable disease-predominant group, with 88 (45%) countries in this group. Of the countries in this group, most transitioned from being in the injury-excess group in 1990 (49 [56%]), but 11 (13%) made the transition from multi-burden to non-communicable disease predominant (Bolivia, Egypt, Kyrgyzstan, the Maldives, Morocco, Peru, Samoa, Sri Lanka, Tajikistan, Turkmenistan, and Uzbekistan). In 2016, the multi-burden group comprised 70 (36%) countries, which were predominantly in sub-Saharan Africa and south Asia. The 37 (19%) countries in the injury-excess group in 2016 (including nine countries that had transitioned from the multi-burden group) were predominantly in eastern Europe, central Asia, and Latin America.

Population-level changes are shown in figure 1C. In 1990, the global population of individuals aged 10–24 years was estimated to be 1·53 billion people (783·0 million males and 750·8 million females), with 713·1 million (46%) living in multi-burden countries, 725·9 million (47%) in injury-excess countries, and 94·8 million (6%) in non-communicable disease-predominant countries. In 2016, the adolescent population had increased to 1·8 billion people (929·9 million males and 871·4 million females). This population growth largely occurred in countries that were classified as multi-burden in 2016 (appendix). As a result, the global population share of adolescents living in multi-burden countries increased from 46% in 1990 to 53% (962·8 million) in 2016, an increase of 249·6 million adolescents. In 2016, 239·3 million (13%) adolescents lived in countries classified as injury excess, a decrease of 486·5 million adolescents since 1990. By 2016, the proportion of adolescents living in countries that were non-communicable disease predominant had increased to 599·3 million (33%), an increase of 504·4 million people. Population change by World Bank income group are shown in the appendix; 945 million (60%) adolescents in 1990 lived in low-income countries, whereas in 2016 most adolescents lived in lower-middle-income countries (835 million [46%]) and upper-middle-income countries (544 million [30%]). India and China together accounted for a third (622 million [35%]) of the global population of adolescents in 2016. India's adolescent population increased by 40%, from 264 million in 1990 to 370 million in 2016; however, China's adolescent population decreased by 28% from 352 million in 1990 to 253 million in 2016 (appendix).

### Indicator estimates for country groupings and individual countries

**Figure 2:**
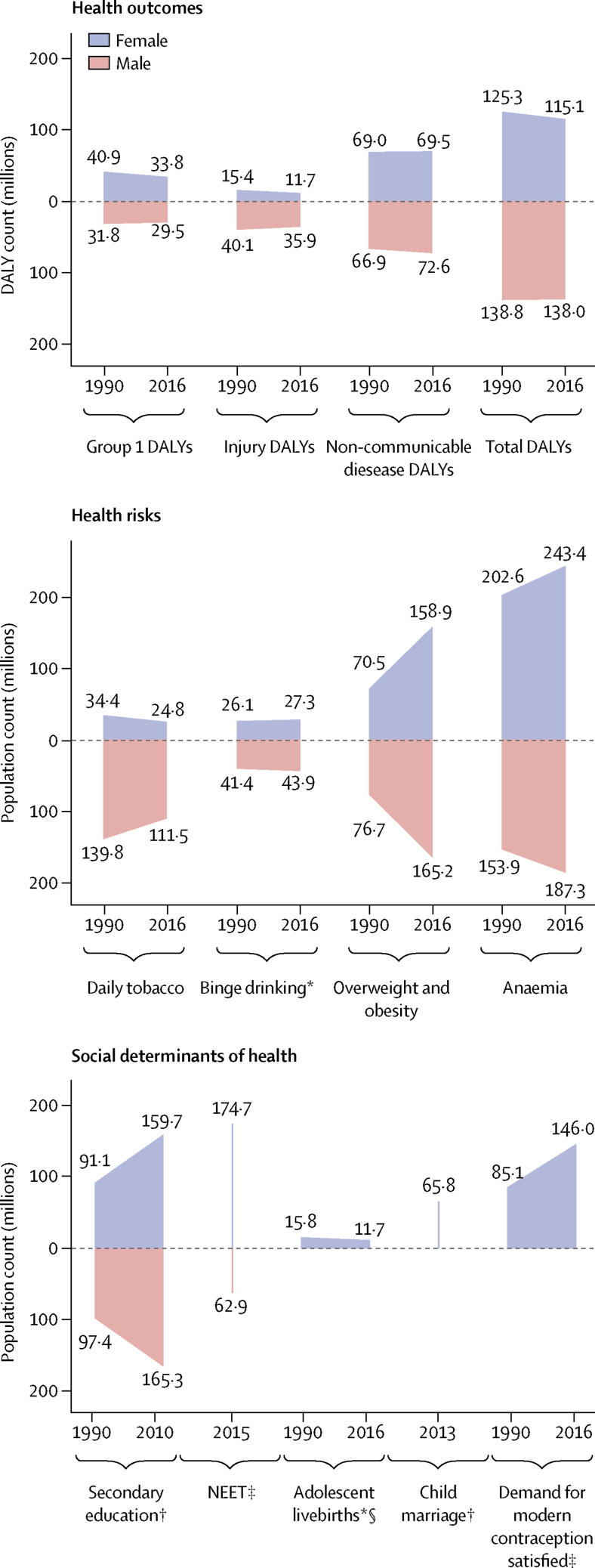
Global counts for 12 indicators of adolescent health in 1990 and 2016, by sex Data are DALY counts in millions, or population in millions. Each indicator is shown at two timepoints, except for NEET and child marriage because insufficient data were available. Counts for NEET, child marriage, and secondary education are estimated using group-specific prevalences (on the basis of available data) and applied to total denominator counts. Data are for adolescents aged 10–24 years, unless otherwise stated. DALYs=disability-adjusted life-years. NEET=not in education, employment, or training. *For individuals aged 15–19 years. †For individual aged 20–24 years. ‡For individuals aged 15–24 years. §Counts for livebirths are incident births, and do not include girls aged 15–19 years who gave birth before 2016.

**Figure 3:**
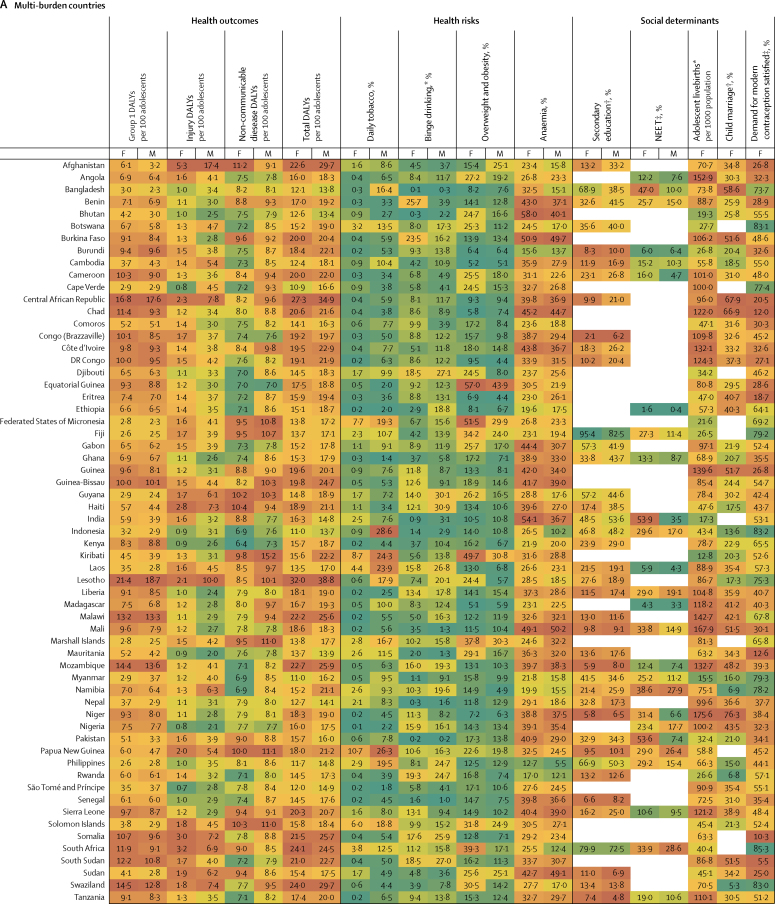

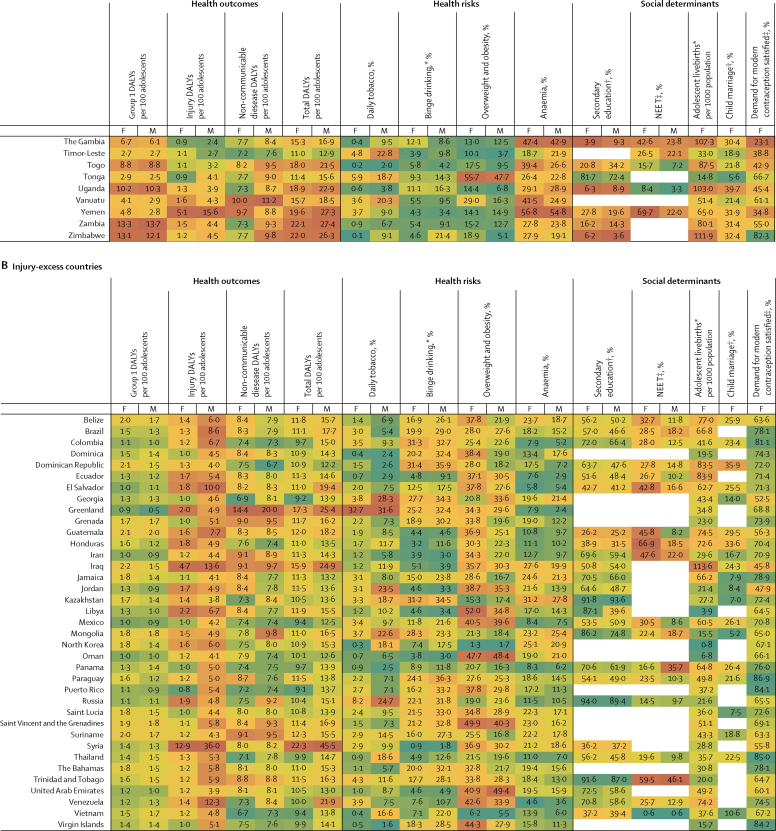

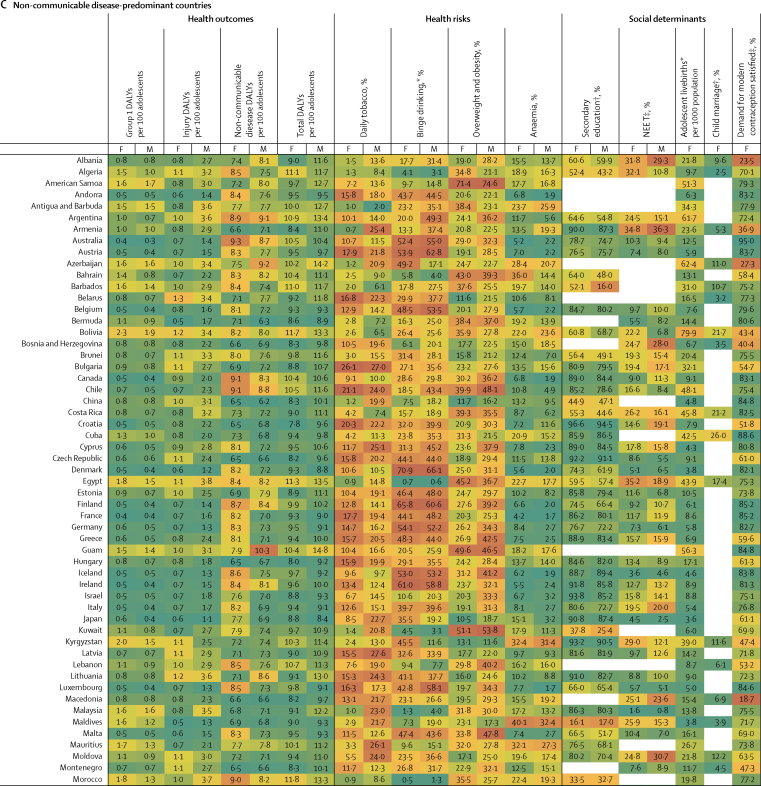

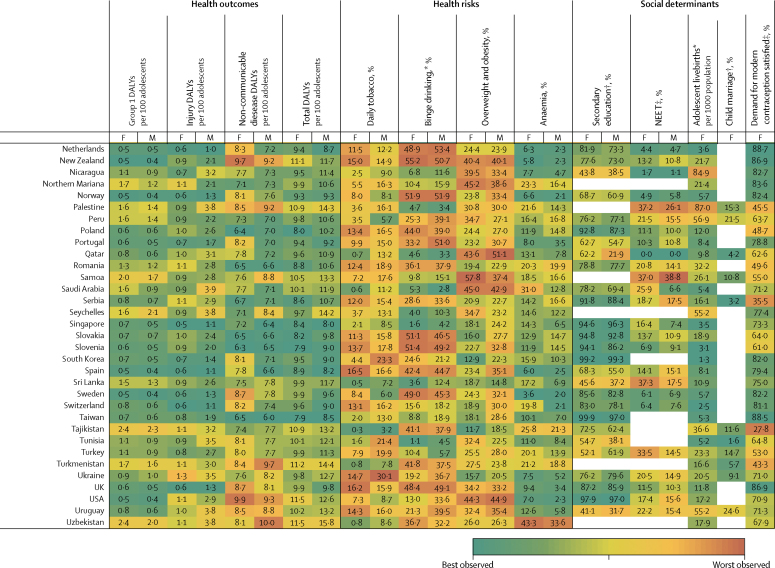
Multi-burden (A), injury-excess (B), and non-communicable disease-predominant (C) country-specific estimates of the 12 indicators for adolescent health and wellbeing, by sex Data are most recent country-level estimates for each indicator (2010 for education [except New Zealand, 2005], median of 2013 for child marriage, median of 2015 for NEET, and 2016 for all others), blank spaces indicate missing data. Health outcomes are DALYs per 100 adolescents, and health risks and social determinants are prevalences, unless otherwise stated. Data are for adolescents aged 10–24 years, unless otherwise stated. For each indicator and sex, countries are shaded on a scale from green (best value observed) to red (worst value observed); for most indicators green signifies the lowest value with the exception of secondary education and demand for contraception satisfied for which it signifies the highest value. DALYs=disability-adjusted life-years. F=females. M=males. NEET=not in education, employment, or training. *For individuals aged 15–19 years. †For individual aged 20–24 years. ‡For individuals aged 15–24 years.

Estimates for each indicator globally and across the three country groupings (including annual rates of change) are shown in table 2. Estimates of the global counts of adolescents for each indicator are shown in figure 2; counts across the three country groupings are in the appendix. Indicator estimates for individual countries are shown in figure 3, with the distribution of estimates and country-level rates of change shown in the appendix. Here we provide a summary of key findings by indicator, presenting trends at a global and country-group level, and then some key findings at a country level.

**Table 2:** Contemporary estimates for indicators of adolescent health across the three country groups and rates of change since 1990

	**Health outcomes**				**Health risks**				**Social determinants of health**				
	Group 1 DALYs	Injury DALYs	Non-communicable disease DALYs	Total DALYs	Daily tobacco	Binge drinking*	Overweight and obesity	Anaemia	Secondary education†	NEET‡	Adolescent livebirths* (per 1000 population)	Child marriage†	Demand for modern contraception satisfied‡
**Multi-burden**													
Female	6334·5 (−1·6%)	1477·7 (−1·1%)	8259·2 (−0·6%)	16 071·4 (−1·1%)	1·5% (+1·2%)	4·3% (+0·7%)	13·0% (+5·7%)	40·2% (−0·4%)	42·1% (+14·9%)	40·3% (NA)	54·2 (−1·7%)	38·2% (NA)	53·0% (+2·1%)
Male	5141·5 (−1·3%)	3723·5 (−0·5%)	8121·8 (−0·3%)	16 986·8 (−0·7%)	9·2% (−0·4%)	7·2% (+0·4%)	11·1% (+4·8%)	29·3% (−0·4%)	43·0% (+9·5%)	8·3% (NA)	··	··	··
**Injury excess**													
Female	1372·0 (−1·4%)	1780·6 (−0·8%)	7837·4 (−0·2%)	10 990·0 (−0·5%)	2·8% (−2·1%)	13·5% (+0·2%)	28·1% (+3·3%)	14·3% (−0·7%)	62·1% (+3·7%)	26·7% (NA)	52·6 (−1·0%)	24·0% (NA)	72·7% (+1·0%)
Male	1226·9 (−1·4%)	7242·7 (−0·3%)	8054·3 (−0·1%)	16 523·9 (−0·4%)	11·3% (−1·5%)	20·3% (+0·1%)	25·6% (+3·8%)	11·1% (−0·8%)	56·0% (+3·0%)	12·7% (NA)	··	··	··
**Non-communicable disease predominant**													
Female	917·6 (−1·8%)	961·5 (−1·9%)	7567·2 (−0·6%)	9446·4 (−1·0%)	5·1% (−1·2%)	17·0% (+0·3%)	22·7% (+3·9%)	13·6% (−0·3%)	61·1% (+0·3%)	18·4%§(NA)	14·1 (−1·8%)	3·58%¶(NA)	78·1% (+0·6%)
Male	798·9 (−1·6%)	2766·9 (−1·7%)	7201·9 (−0·6%)	10 767·7 (−1·0%)	16·8% (−1·0%)	24·0% (+0·4%)	25·3% (+3·5%)	9·1% (−0·3%)	60·5% (+0·2%)	12·6%§(NA)	··	··	··
**Global**													
Female	3880·2 (−1·1%)	1348·0 (−1·2%)	7974·1 (−0·5%)	13 202·4 (−0·8%)	2·8% (−1·5%)	9·6% (−0·3%)	18·2% (+3·6%)	27·9% (+0·1%)	53·3% (+1·9%)	32·8%§(NA)	41·2 (−1·3%)	22·8%¶(NA)	66·6% (+0·7%)
Male	3171·5 (−0·9%)	3865·8 (−0·9%)	7804·7 (−0·4%)	14 841·8 (−0·6%)	12·0% (−1·2%)	14·4% (−0·3%)	17·8% (+3·1%)	20·1% (+0·1%)	52·6% (+1·5%)	10·0%§(NA)	··	··	··

Data are DALYs per 100 000 adolescents for health outcomes and prevalence for health risks and determinants with the exception of livebirths, which are per 1000 population, with the annual rate of change in parentheses where available. Estimates are the most recent available (2010 for education [except New Zealand, 2005], median of 2013 for child marriage, median of 2015 for NEET, and 2016 for all others) for adolescents aged 10–24 years, unless otherwise stated. Data were available for over 80% of the denominator population, unless otherwise indicated. DALYs=disability-adjusted life-years. NA=not applicable. NEET=not in education, employment, or training.

* For individuals aged 15–19 years.

† For individual aged 20–24 years.

‡ For individuals aged 15–24 years.

§ Coverage was only 50–80% of the denominator population of this country group.

¶ These estimates for child marriage are based on low coverage primary data (18%; see methods for detail on estimation).

### Health outcomes

Adolescents had a total burden of 253 million DALYs in 2016 (figure 2); 159 million DALYs (63%) were among adolescents in multi-burden countries; 33 million (13%) in injury-excess countries; and 61 million (24%) in non-communicable disease-predominant countries (appendix). A global decrease of 11 million DALYs occurred between 1990 and 2016, largely driven by epidemiological change (appendix). The prevalence of DALYs was highest in the multi-burden group and lowest in the non-communicable disease-predominant group for both males and females (table 2). Males in the injury-excess group had a DALY prevalence similar to males in the multi-burden group (table 2). Uncertainty estimates for all cause DALYs, YLL, and YLDs are reported in the appendix

In 2016, group 1 conditions accounted for 25% (63 million of 253 million DALYs) of the global DALY burden for adolescents (figure 2). 87% (55 million) of DALYs due to group 1 conditions were borne by adolescents in the multi-burden country group, with the prevalence of group 1 DALYs being 20% higher in females than in males in these countries (table 2; appendix). However, the global annual rate of change in group 1 DALYs since 1990 was greater among females (−1·1%) than males (−0·9%), with a global decrease of 9·4 million DALYs due to group 1 conditions between 1990 and 2016 (figure 2; appendix). Of multi-burden countries, Rwanda had the greatest annual decrease in group 1 DALYs from 1990 to 2016: −2·8% for females and −2·9% for males (appendix). In 2016, Lesotho and Central African Republic, both multi-burden countries, had the highest group 1 DALY prevalence for females and males (>15 000 DALYs per 100 000 population), whereas Australia and France had prevalences of less than 500 DALYs per 100 000 population (figure 3). DALY prevalence due to group 1 conditions decreased in almost all countries, except in four multi-burden countries (Lesotho, Swaziland, South Africa, Zimbabwe) and two injury-excess countries (Virgin Islands and Mozambique; appendix). Between 1990 and 2016, females in Lesotho had the greatest annual increase in group 1 DALYs (6·8%) of any sex in any country. Uncertainty estimates for country-level estimates of group 1 DALYs are shown in the appendix.

Injuries accounted for almost a fifth (48 million [19%]) of the total DALYs for adolescents in 2016 (figure 2). In 2016, 53% (25 million) of DALYs due to injury were borne by adolescents in multi-burden countries, and 11 million (23%) were in injury-excess settings (despite comprising only 13% of the global population of adolescents; appendix). Globally, DALY prevalence due to injuries was almost three times higher among males than females (table 2). This sex difference was more substantial in injury-excess countries than in the other country groups, with the prevalence of DALYs for males four-times higher than for females (table 2). As a result, males carried almost 75% of all DALYs due to injury (35·5 million of 48 million DALYs) in 2016, with almost 40% of the global injury burden being among males in multi-burden settings (18 million DALYs; appendix). Although the global DALY count decreased by 7·8 million DALYs due to injury between 1990 and 2016 (figure 2), males had a smaller rate of annual change than females did, particularly in injury-excess countries (table 2). In 2016, the highest prevalence of DALYs due to injury (>10 000 per 100 000 adolescents) for males were in the injury excess countries Syria, Iraq, and Venezuela, and the multi-burden countries Afghanistan, Yemen, and Lesotho (figure 3). In 2016, the prevalence of injury DALYs for females were highest (>5000 per 100 000) in Syria, Afghanistan, Yemen, and Iraq. Syria had an average annual increase in the prevalence of injury DALYs of 42·8% for females and 38·0% for males between 1990 and 2016 (appendix).

In 2016, non-communicable diseases were the leading contributor to disease burden in adolescents, accounting for 56% (142 million) of 253 million of global adolescent DALYs. 55% (78·8 million DALYs) of the global non-communicable disease burden was borne by adolescents in multi-burden countries, and 31% (44·2 million DALYs) by adolescents in non-communicable disease-predominant countries (appendix). In 2016, the prevalences of DALYs due to non-communicable diseases were higher in multi-burden and injury-excess countries than in non-communicable disease-predominant countries (table 2). Rates of change in DALYs were substantially lower for DALYs due to non-communicable disease than for DALYs due to injuries or group 1 conditions (table 2). An overall increase of 6·1 million DALYs due to non-communicable diseases between 1990 and 2016 was driven largely by population growth (appendix). Greenland, an injury-excess country, and several multi-burden countries in the Pacific (eg, Kiribati, Solomon Islands, Vanuatu, and Papua New Guinea) had the highest prevalences of DALYs from non-communicable diseases (figure 3). We could not calculate uncertainty estimates for the modified groups and so we provide country-level uncertainty estimates for IHME level 1 groups of injury, non-communicable disease, and separately for self harm in the appendix.

### Health risks

In 2016, 136 million adolescents smoked daily (112 million males and 25 million females), a decrease of 38 million compared with 1990 (figure 2). Of smokers in 2016, 67 million (49%) lived in non-communicable disease-predominant countries and 52 million (38%) in multi-burden countries (appendix). Daily smoking prevalence was higher among males across all country groups, and was highest in the injury-excess (13·8 million [11%] of 122 million males and 3·3 million [3%] of 117 million females; table 2) and non-communicable disease-predominant group (52·4 million [17%] of 312 million males, 14·7 million [5%] of 287·6 million females; table 2). The prevalence of tobacco use decreased from 1990 to 2016, except among females in multi-burden countries where, although levels remain low, a small annual increase has occurred since 1990 (1·2%; table 2). Furthermore, in 2016, 40% (45·4 million of 111·5 million) of male daily smokers and 27% (6·8 million of 24·8 million) of female daily smokers were in multi-burden countries, which is a substantial increase compared with 1990 (41·1 million [30%] of 139·8 million male smokers and 4·1 million [12%] of 34·4 million female smokers. In 2016, smoking prevalence was over 20% for females in the injury-excess country Greenland, and in the non-communicable disease-predominant countries Bulgaria, Chile, and Croatia (figure 3). For males, the smoking prevalence was over 25% in the multi-burden countries Indonesia and Papua New Guinea, the injury-excess countries Greenland and Georgia, and the non-communicable disease-predominant countries Ukraine, Latvia, Bulgaria, Mauritius, Armenia, and Cyprus (figure 3). Smoking increased more than 5% annually from 1990 to 2016 for females living in Egypt, Montenegro, South Korea, Kyrgyzstan, India, and Palestine, and for males living in Montenegro (appendix).

In China in 2016, 20% (26·8 million of 134·4 million) of males were smoking daily compared with 1% (1·4 million of 118·3 million) of females, with an annual change of −0·8% for males and −1·9% these prevalences are decreasing (appendix). Conversely, smoking prevalence is increasing in India (6·4% annual increase for females and 0·4% annual increase for males), although the prevalence in 2016 was relatively low, with 3% (4·3 million of 175·1 million) of females and 8% (14·8 million of 195·0 million) of males smoking daily (appendix).

In 2016, 71 million adolescents aged 15–19 years reported binge drinking in the past 12 months (44 million males and 27 million females), a small increase since 1990 (41 million males and 26 million females; figure 2). 39 million (55%) adolescents who reported binge drinking lived in non-communicable disease-predominant countries, 19 million (26%) lived in multi-burden settings, and 13 million (19%) lived in injury-excess countries (appendix). However, the prevalence of binge drinking was highest in countries that were non-communicable disease predominant (23·7 million [24%] of 98·7 million males and 15·5 million [17%] of 91·1 million females aged 15–19 years) and injury excess (8·1 million [20%] of 40·0 million males and 5·2 million [14%] of 38·3 million females aged 15–19 years). The prevalence of binge drinking changed little over time and females in multi-burden countries had the most substantial annual increase from 1990 to 2016 (0·7%). Substantial differences were observed between countries (figure 3). The prevalences of binge drinking among females aged 15–19 years living in Ireland, Denmark, New Zealand, and Finland—all non-communicable disease-predominant countries—were greater than 55% (figure 3). For males, the prevalence of binge drinking was over 60% in Austria, Denmark, and Finland—also all non-communicable disease-predominant countries (figure 3). By contrast, the multi-burden countries Bangladesh and Pakistan and the non-communicable disease predominant country Egypt had prevalences under 1% (figure 3). Ethiopia showed the greatest overall annual increase between 1990 and 2016 (2·3% for females and 3·6% for males), whereas Zambia had the greatest decrease (−2·0% for females and −2·2% for males), both of which are multi-burden countries (appendix). In China in 1990, the prevalence of binge drinking was 5% in females (2·8 million of 60·1 million) and 15% in males (9·4 million of 63·6 million), which had increased by 2016 to 8% for females (2·7 million of 35·8 million) and 18·2% (7·4 million of 40·8 million). In India in 2016, prevalence of binge drinking was low, at 1% (0·5 million of 58·7 million) for females and 3% (2·0 million of 65·4 million) for males. The prevalence of binge drinking in India appears relatively steady over time, with the rate of change from 1990 to 2016 varing by less than a percent for females (−0·5%) and males (0·4%).

In 2016, almost one in five (324 million [18%]) of the world's adolescents was overweight or obese; a 120% (176·8 million) increase from the 147·3 million adolescents who were overweight or obese in 1990 (figure 2). This increase in overweight and obese adolescents was driven by changes in the prevalence of overweight and obesity rather than population change (appendix). In 2016, 144 million (44%) overweight or obese adolescents lived in non-communicable disease predominant countries and 116 million (36%) in multi-burden countries. In 2016, the prevalence of overweight and obesity was highest for adolescents in the non-communicable disease-predominant (144·1 million [24%] of 600 million) and injury-excess countries (64 million [27%] of 239 million), with higher prevalences among males than females in non-communicable disease-predominant countries, and higher prevalences among females than males in the injury-excess and multi-burden countries (table 2). Since 1990, a substantial increase in overweight and obesity occurred annually for both males and females in all country groups, but this increase was particularly prominent for females in multi-burden countries (5·7%; table 2). Females living in the non-communicable disease-predominant countries American Samoa and Samoa and the multi-burden countries Equatorial Guinea and Tonga had the highest prevalences of overweight and obesity, all more than 55% (figure 3). For males, prevalence was greater than 50% in the non-communicable disease-predominant countries American Samoa, Kuwait, and Qatar (figure 3). Only one country (Democratic Republic of the Congo) had a decrease in overweight and obesity over time (appendix). Both China and India had large increases in the prevalence of overweight and obesity, with males in both countries having an annual rate of change of 6·2%, whereas females had annual rates of change of 4·8% in China and 8·5% in India (appendix)

Anaemia affected almost one in four adolescents globally (430 million [24%]) in 2016, an increase of 20% from 357 million in 1990. This increase in the number of adolescents with anaemia was largely driven by changing demography (appendix). In 2016, 333 million (77%) adolescents with anaemia lived in multi-burden settings, an increase of 75 million compared with 1990 (appendix). 45% (194 million) of adolescents with anaemia lived in India and China. The prevalence of anaemia was higher for females than for males across all groups, and was highest for both males and females in multi-burden countries, with 40% (188 million of 467 million) of females in multi-burden countries having anaemia (table 2). Prevalence of anaemia decreased over time for all country groups for both males and females but the rate of change was small, with an annual decrease of only 0·4% for both sexes in multi-burden countries. Prevalence of anaemia was more than 50% for females in the multi-burden countries Bhutan, Yemen, India, and Burkina Faso. For males, prevalence was highest (>45%) in the multi-burden countries Yemen, Mali, Burkina Faso, and Sudan. The lowest prevalences of anaemia (<4%) averaged between the sexes were in non-communicable disease-predominant countries—specifically, Sweden, France, Australia, Denmark, Belgium, and Ireland (<4%; figure 3).

### Social determinants of health

Globally in 2010, an estimated 325 million adolescents aged 20–24 years (just over half the total population of 620 million adolescents aged 20–24 years [303 million males and 317 million females]) had completed secondary education, an increase of 136 million since 1990. However, with little change from the estimated 300 million not completing education in 1990, an estimated 295 million (61%) adolescents aged 20–24 years had not completed secondary education in 2010 (159 million in multi-burden countries, 36 million in injury-excess countries, and 100 million in non-communicable disease-predominant countries). The proportion of adolescents who completed secondary education was lowest in multi-burden countries, with only 42% (49·2 million of 116·8 million) of females and 43% (52·4 million of 121·8 million) of males aged 20–24 years, estimated from 47 multi-burden coutries with data, completing secondary education in the multi-burden group in 2010 (table 2). However, the greatest increases in completion of secondary education have been in multi-burden countries, with a 14·9% annual increase for females aged 20–24 years since 1990. Females living in the non-communicable disease-predominant countries Taiwan, South Korea, the USA, and Croatia, and the multi-burden country Fiji had secondary school completion greater than 95% (figure 3). For males, prevalence of secondary school completion was highest (>95%) in the non-communicable disease-predominant countries South Korea, the USA, Taiwan, and Singapore (figure 3). Several multi-burden countries had prevalences of completion of less than 5%: Congo and The Gambia for females, and Zimbabwe and Tanzania for males (figure 3). Large differences in secondary school completion by sex were seen in some countries. Females living in Barbados, Qatar, and Libya were twice as likely to complete secondary education as males were. By contrast, males living in Congo, Afghanistan, The Gambia, Haiti, Central African Republic, and Democratic Republic of Congo were twice as likely to complete secondary education as females were (figure 3). Females living in the multi-burden countries Yemen, India, Malawi, and Mali and the injury-excess country Vietnam had more than 30% annual increases in secondary school completion between 1990 and 2010 (appendix).

Most recent estimates indicate 238 million adolescents aged 15–24 years were NEET (175 million females and 63 million males), of whom 60% (142 million adolescents, 117 million females and 25 million males) lived in multi-burden settings. Global prevalence of NEET was around three times higher among females (33% [138 million of 421 million]) than among males (10% [44·4 million of 443·0 million]) aged 15–24 years, estimated from 123 countries with available NEET data, with the sex difference being particularly noticeable for multi-burden countries (table 2; appendix). Females living in the multi-burden countries Yemen, India, and Pakistan, and the injury-excess countries Honduras, and Trinidad and Tobago had NEET prevalences of more than 50% (figure 3). Males living in the injury-excess countries Trinidad and Tobago and Panama and the non-communicable disease-predominant countries Samoa and Armenia had prevalences of NEET of more than 35% (figure 3). Large sex differences in NEET were seen in India, with the prevalence of NEET among female adolescents being more than 15 times higher than among males, and in Pakistan, being more than seven times higher than among males (figure 3). Insufficient data were available to calculate the rate of change in NEET prevalence for all countries (appendix).

In 2016, adolescent mothers aged 15–19 years had 11·7 million livebirths (this number is for new births, and does not include girls aged 15–19 years who gave birth before 2016), a 25% decrease from 15·7 million in 1990. In 2016, 73% (8·5 million) of adolescent livebirths occurred in multi-burden countries, 17% (2 million) in injury-excess countries, and 11% (1·3 million) in non-communicable disease-predominant countries (appendix). The rate of livebirths among adolescents was highest in multi-burden and injury-excess countries compared with non-communicable disease-predominant countries (table 2). The rate of livebirths among adolescents decreased substantially across all country settings, but the smallest decrease was in the injury-excess group (table 2). Of the 30 countries with the highest adolescent livebirth rate, 28 (93%) were classified as multi-burden (figure 3). Niger had the highest rate of adolescent births (175 livebirths per 1000 females aged 15–19 years per year), with Mali, Angola, Malawi, Guinea, Mozambique, and Côte d'Ivoire all having rates between 167 and 132 livebirths per 1000 females aged 15–19 years per year. Of the 50 countries with the lowest rates of adolescent births, 46 (92%) were non-communicable disease-predominant countries (figure 3). South Korea, North Korea, and Switzerland had rates of adolescent births below three per 1000 females aged 15–19 years per year. Ten countries had an increase in the rate of adolescent livebirths between 1990 and 2016: the non-communicable disease-predominant countries Azerbaijan, Malta, and Albania; the injury-excess countries Iraq and Vietnam; and the multi-burden countries the Philippines, Zimbabwe, Lesotho, Cambodia, and Democratic Republic of the Congo (appendix).

For child marriage, data were only available for 31 (35%) of 88 countries in the non-communicable disease-predominant group, which covered 18% of females aged 20–24 years in this group. Using data from 2003–16 (the most recent year of data collection varied between countries, median year of data collection was 2013), we estimated that 66 million females aged 20–24 years reported being married before the age of 18, of whom an estimated 51·5 million (79%) lived in multi-burden countries, 10·1 million (15%) lived in injury-excess countries, and 4·0 million (6%) lived in non-communicable disease-predominant countries. Prevalence of child marriage was high in multi-burden countries, with 38% (50·5 million of 132·3 million females estimated from 66 countries with available data) of females aged 20–24 years in the multi-burden group married before age 18 years. Niger, Chad, Central African Republic, Mali, Bangladesh, Guinea, Burkina Faso, and South Sudan—all multi-burden countries—had prevalences of child marriage of more than 50% (figure 3). Child marriage was also high (>20%) in some non-communicable disease-predominant countries situated predominantly in central America and Latin America, including Cuba, Uruguay, Bolivia, Peru, and Costa Rica (figure 3). Rate of change could not be calculated because of the lack of data.

In 2016, of 219·1 million females aged 15–24 years, 146·0 million (67%) had their demand for contraception satisfied with modern methods (49·5 million [34%] in multi-burden countries, 23·5 million [16%] in injury-excess countries, and 73 million [50%] in non-communicable disease-predominant countries), an increase of 61 million since 1990 (figure 2; appendix). Despite this increase, unmet demand also increased from 68·4 million (45%) in 1990 to 73·1 million (33%) in 2016. The prevalence of females who had their contraceptive needs met was higher in non-communicable disease-predominant countries than in injury-excess and multi-burden countries (table 2). Rates of annual improvement since 1990 were greatest in multi-burden countries (2·1%; table 2). Countries that notably had a low prevalence of demand for contraception being satisfied (ie, <20%) were the multi-burden countries South Sudan, Somalia, Chad, Mauritania, and Eritrea, and the non-communicable disease-predominant country Macedonia (figure 3). Countries with the highest prevalence of demand satisfied (>85%) were mostly in the non-communicable disease-predominant group, including Australia, the Netherlands, Cuba, Taiwan, New Zealand, the UK, France, and Finland, but also the injury-excess countries Thailand and Paraguay, and the multi-burden country South Africa (figure 3). Among all countries, the multi-burden countries Burundi, Senegal, and Guinea-Bissau had the most substantial improvements in demand satisfied, each with a more than 25% increase annually (appendix). Prevalence of demand satisfied decreased in ten countries over time; Serbia, a non-communicable disease-predominant country, and Guyana, multi-burden country, had annual decreases in prevalence of more than 1% (appendix).

## Discussion

Great changes have occurred in adolescent health over the past 25 years. The decrease in disease burden due to group 1 conditions and injuries has been particularly notable, with most countries now predominant in non-communicable diseases. However, the epidemiological transition has coincided with rapid demographic changes. Today's non-communicable disease-predominant and injury-excess countries have fewer adolescents than they did in 1990; by contrast, the number of adolescents in multi-burden countries has greatly increased. As a result, 35% more adolescents were living in multi-burden countries in 2016 than in 1990. Shifts in health risks since 1990 were equally sobering. Nutritional risks have become more prominent than in 1990, with the number of overweight or obese adolescents more than doubling and the number of adolescents with anaemia increasing by 20% during this period. Despite having been a focus of policy attention in many high-income countries, the number of adolescents who binge drink has not shifted since 1990, and although the global number of adolescent daily smokers has decreased by around 20%, the global prevalence among males aged 10–24 years is still over 10%.

One area of clear progress has been in secondary school completion, particularly for girls. Even so, only around half of the world's adolescents are completing secondary education and the absolute number of adolescents not completing secondary education in 2010 is little changed from 20 years earlier. Progress was seen in livebirths to adolescent mothers, an important determinant of health for girls and young women, but the rate of livebirths remains high in many multi-burden and injury-excess countries. Continued high prevalences of child marriage and substantial inequalities in NEET between the sexes suggest that inequitable gender norms remain powerful determinants of adolescent health and wellbeing.

Satisfaction of demand for contraception for females was included as an indicator of the extent to which health systems can deliver an essential resource for health that is sensitive to social norms. We observed improvements for this indicator across each country grouping, particularly for multi-burden countries, but with great variation between countries in the prevalence of demand satisfied. However, in 2016, 73 million young women did not have their demand for contraception met with modern methods, an increase of 5 million since 1990, suggesting that improvements in access to contraception have not kept pace with need.

These changes in adolescent health outcomes, health risks, and social determinants must be interpreted in light of demographic changes. Although the prevalences of tobacco smoking are still lower in most multi-burden countries than in most non-communicable disease-predominant countries, 41% of male and 27% of female daily smokers now live in multi-burden countries, a substantial increase compared with 1990 (29% of male and 12% of female daily smokers). High prevalences of tobacco use (>25%) for males in multi-burden (eg, Indonesia and Papua New Guinea) and injury-excess countries (eg, Georgia and Greenland) highlight where tobacco control efforts are urgently needed. This need is also true for countries where the prevalence of smoking is increasing rapidly, such as for females in Palestine and India. Equally, although the prevalence of anaemia has decreased in multi-burden countries, this decrease has been out-paced by population growth so that, compared with 1990, an additional 74 million adolescents with anaemia were living in multi-burden countries in 2016. This increase in unmet adolescent health needs has emerged despite growing availability of evidence-based interventions.^1^ Unmet need around adolescent non-communicable diseases and associated health risks remains a priority in countries that have passed through the epidemiological transition. However, the greatest challenges in adolescent health are in low-resource multi-burden countries where adolescents commonly face a triple disease burden from group 1 conditions, injuries, and non-communicable diseases, and health systems have largely not kept pace with demographic change.

Therefore, multi-burden countries are a priority for advancing global adolescent health. To achieve the target of no country being in the multi-burden disease group by 2030,^1^ group 1 conditions would need to decrease by almost 4000 DALYs per 100 000 adolescents in the next 15 years, an annual decrease of approximately 4%. This annual change is about three times more than that observed in the multi-burden group for 1990–2016; only Rwanda in this group achieved an annual decrease of almost 3%. Tackling adolescent health in multi-burden countries will need to accelerate progress around structural and social determinants of health, including access to quality education and health services, provision of opportunities for employment, and achieving equitable gender norms.^1^ Our analysis highlights the need to address non-communicable diseases for all adolescents, and since mental disorders are a major contributor to non-communicable disease burden, a specific focus on mental disorders will be needed,^22^ and chronic and relapsing physical health problems that affect adolescents in all settings.^1^

Overweight and obesity emerged as pervasive health risks, with the highest prevalence in non-communicable disease-predominant countries, but with rates of change increasing rapidly globally. Given that recovery from adolescent overweight and obesity is rare once established, the consequences on health in later life and for the next generation will be great.^2^ Given the health implications, the omission of adolescent overweight and obesity from the SDGs is a major policy gap.

Some improvements probably reflect action under the Millennium Development Goals, including a lower sex disparity in educational attainment than in 1990 and decreases in the rate of adolescent births.^23^ Despite progress in education, 117 million young women in multi-burden countries are NEET, comprising almost half of all adolescents who are NEET. Although essential, promotion of education alone will be insufficient to achieve equity between the sexes in the social determinants of health and wellbeing during adolescence. In part, this high prevalence of NEET among young women in multi-burden countries could be explained by continuing high rates of adolescent livebirths, which disrupts education and in turn restricts prospects for employment.^24^ In many multi-burden countries, prevalences of child marriage also continue to be high, reflecting harmful gender norms. Achieving gender equity in determinants of adolescent health and wellbeing will require action on many fronts, including employment and economic empowerment, better access to essential health care including contraception, implementation of legislative frameworks to protect girls from early marriage, and changes in community norms.

Beyond education, HIV, and sexual and reproductive health, adolescents have been largely absent in global health investments.^25^ Perhaps reflecting this neglect, the overall annual decrease in global disease burden for adolescents of 0·8% for females and 0·6% for males was less than the decrease of 1·2% annual change in the age-standardised rate of total DALYs found by GBD 2016 between 1990 and 2016.^18^ Here we found 53% of adolescents in multi-burden countries had their demand for modern contraception satisfied in 2016, and Niamh Cahill and colleagues' found a prevalence of less than 68% among married women living in 69 of the world's poorest countries in 2017.^26^ These findings are likely to reflect lower health-care access among adolescents than among adults, and unmet needs are likely to be even greater for other health needs such as mental disorders.^8^

China and India are home to a third of the world's adolescents and present contrasting examples of adolescent health. China has progressed rapidly from an injury-excess country to a non-communicable disease-predominant country at a time when its adolescent population has decreased by 28%. Although improvements in adolescent health outcomes in China have been impressive, the prevalence of smoking and binge drinking is high (higher for males than for females), and prevalences of overweight and obesity are increasing. By contrast, India has remained a multi-burden country and the number of adolescents has increased by 40%. Health risks in India, although relatively low by global standards, are increasing rapidly, but perhaps more pressing is the large disease burden and gender inequality across the social determinants. The USA stands out for its poor adolescent health compared with other high-income or non-communicable disease-predominant countries. Although not as high as in injury-excess countries, injury-related DALYs in the USA were higher than in similar high-income countries. 17·4% of females and 15·6% of males aged 15–24 years in the USA were NEET in 2016. The USA's comparatively low prevalence of demand for modern contraception satisfied (70·9%) also raises questions about the responsiveness of the health system, while the high prevalence of overweight and obesity (44·3% for females and 44·9% for males) suggests a lack of public health responses.

The Commission's headline indicators were designed to capture shifts in adolescent health with progress through the epidemiological transition.^1^ Since mortality alone is a poor indicator of health for adolescents compared with other age groups, we used DALYs as a summary measure of health need. DALYs rely on good primary data to estimate their YLL and YLD components, which even for mortality are often lacking for the adolescent age groups.^22^, ^27^ Grouping countries by disease burden appears to be useful for policy and programming because both health risks and social determinants of health tended to cluster by these country groups. This observation suggests that this grouping by disease burden might be useful in indicating a package of actions relevant to countries within a group. In particular, injury-excess countries are a group that cannot be readily identified by their level of socioeconomic development or income grouping.^28^

To populate indicators of health risks and determinants, some modifications of the definitions were needed. The most notable change was for the indicator of iron deficiency anaemia as an indicator of poor nutritional status, which we modified to anaemia. Because most cases of anaemia globally have a primary nutritional origin, anaemia remains useful as an indicator of undernutrition and is particularly relevant for adolescents in the context of rapid growth and menstruation.^29^ We defined the indicator of smoking to specifically refer to tobacco smoking to measure smoked products and we did not include emerging behaviours such as vaping. Our measure of binge drinking adopted a commonly used timeframe of 12 months, but a shorter timeframe might have been more useful in defining a clearer risk group. Educational attainment data from Barro-Lee, which had good coverage, was only available up to 2010. We were also unable to stratify these indicators by urban or rural status and socioeconomic wealth as originally proposed. Tracking policy and practice at country and local level will undoubtedly require additional indicators and stratification to capture inequalities across disadvantaged groups including ethnic minorities, Indigenous populations, LGBT+ (lesbian, gay, bisexual, transgender, and other identities), young offenders, and migrants or displaced young people.

Serial estimates of child marriage were not available for many countries and were largely unavailable for non-communicable disease-predominant high-income countries. To present a global estimate, we assumed that in non-communicable disease-predominant countries without data the prevalence of child marriage would be similar to the lowest observed estimates within that disease group (1·6%). This assumption is likely to have resulted in an underestimation of total numbers—eg, in the USA, a country without recent comparable data, in 2002 an estimated 6% of females and 2% of males were married by the age of 18 years.^30^

To maximise data coverage, ten of 12 indicators were populated using modelled data, which enabled better data coverage and is an advance on earlier efforts in which, even with optimal indicator definitions, only seven of 25 indicators covered at least 50% of the world's adolescents.^7^ Modelled data provide more complete estimates, including trends over time, but wide uncertainty estimates show the poor quality and availabilty of underlying primary data for some measures. The uncertainty estimates for DALYs due to non-communicable diseases were both wide and similar across countries, suggesting poor primary data and a heavy reliance on modelling. For countries in conflict and war (eg, Syria and Iraq in 2016), large uncertainty estimates for injury also indicate a paucity of primary data. For estimates of binge drinking for all countries, large uncertainty estimates also indicate the paucity of primary data.^7^

A burgeoning adolescent population in many low-income and middle-income countries could provide an unprecedented demographic dividend through the entry into the workforce of an educated and healthy cohort with fewer dependants than previous generations. A triple burden of adolescent disease, together with continued high prevalences of early marriage and adolescent livebirths in many resource-limited settings, will diminish the likelihood of a demographic dividend. Health risks associated with nutrition and substance use affect adolescent growth and development and ultimately health trajectories in later life. Not addressing social and structural determinants of health, including a lack of quality education, few employment opportunities, continuing high prevalences of early pregnancy, and inequitable gender norms diminish this generation's prospects for health, wellbeing, and economic participation. Health, education, and legal systems have not kept pace with demographic shifts and growing geographical inequality in adolescent health needs. Despite improvements in many settings, the adolescent health challenge is greater today than it was 25 years ago. The case for comprehensive and integrated investments in adolescent health, growth, and development has never been stronger.

**Figure:**
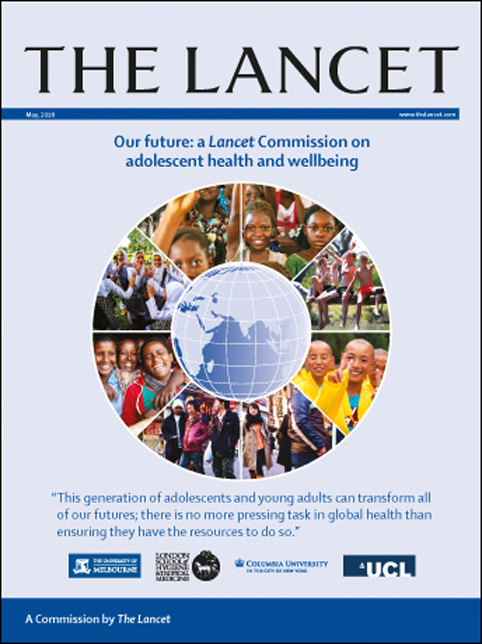

**This online publication has been corrected. The corrected version first appeared at thelancet.com on March 21, 2019**

## Data sharing

Data analysed in this paper are publicly accessible and details of how to access these data are in the appendix.
