# Department of Error

**DOI:** 10.1016/S0140-6736(19)30578-1

**Published:** 2019-03-23

*Azzopardi PS, Hearps SJC, Francis KL, et al. Progress in adolescent health and wellbeing: tracking 12 headline indicators for 195 countries and territories, 1990–2016.* Lancet *2019; **393:** 1101–18—*In figure 3 of this Article, under the Social determinants heading, the text should have read “Adolescent livebirths* per 1000 population”. This correction has been made to the online version as of March 21, 2019.

