# Supplementary material for: Progress in adolescent health and wellbeing: tracking 12 headline indicators for 195 countries and territories, 1990–2016
Source: Lancet. 2019 Mar 16;393(10176):1101–18. doi: 10.1016/S0140-6736(18)32427-9 (PMC6429986; doi:10.1016/S0140-6736(18)32427-9)
Supplement: Supplementary appendix [file mmc1.pdf]

THE LANCET

Supplementary appendix

This appendix formed part of the original submission and has been peer reviewed. We post it as supplied by the authors.

Supplement to: Azzopardi PS, Hearps SJC, Francis KL. Progress in adolescent health and wellbeing: tracking 12 headline indicators for 195 countries and territories, 1990–2016. *Lancet* 2019; published online March 12. http://dx.doi.org/10.1016/S0140-6736(18)32427-9.

Appendices

Appendix 1 – Extended definitions of indicators and data sources

Appendix 2 – Data availability for NEET and Child Marriage

Appendix 3 – Uncertainty bounds for indicators at a country level: health outcomes (DALYs per 100 000 population due to Group 1 condition, Injuries, NCDs, self-harm, total DALYs, total YLL and total YLD), health risks (prevalence of daily tobacco use, binge drinking, anaemia, obesity and overweight) and determinants (Demand for modern contraception satisfied).

Appendix 4 – Population counts for each country and population scenarios

Appendix 5 – Counts of the indicators for the three country groups for two time points (typically 1990 and 2016) and scenarios.

Appendix 6 – Distribution of indicator estimates by the three country groups

Appendix 7 – Rates of change for the indicators by country

Appendix 1 – Extended definitions of indicators and data sources

Indicators were organised into three main categories: health outcomes; health risks; and social determinants of health. The definition of each indicator and data sources are detailed here.

Health outcomes

Disease burden was measured using Disability Adjusted Life Years (DALYs, the sum of years of healthy life lost due to either death or disability) as estimated by GBD2016. Modelled estimates were available for 333 causes for males and females aged 10 - 14 years, 15 - 19 years and 20 - 24 years, for 195 countries between 1990–2015 in five-year intervals, and for 2016. Of the causes included in GBD2016, nine were not modelled to contribute DALYs amongst adolescents (including some neonatal conditions and conditions more typical of later adulthood such as macular degeneration, atrial fibrillation, and Alzheimer's disease). The remaining 324 conditions were distributed across three broad indicators as defined by the Commission: communicable, reproductive and nutritional conditions ('Group 1'' conditions); injuries including assault, homicide and unintentional injuries such as from road traffic injuries; and non-communicable diseases (NCDs) which included chronic physical illness, mental and substance use disorders, and self-harm.[1] These three groups largely reflected level 1 conditions in the GBD hierarchy of causes, with the exception of self-harm which was moved from the GBD injury group to the NCD group in our analyses, given its relationship to mental disorders (consistent with the approach used in the Commission analyses).[1] Note that uncertainty estimates were not available for these modified groups; Appendix 3 reports uncertainty around the GBD level 1 groups and self-harm separately. Indicators were reported as DALYs per 100,000 population, with population denominators sourced from IHME, based on United Nations population estimates. The sum of these three indicators (all-cause DALYs) was the total disease burden experienced by adolescents.

Health risks

Data relating to health risks were derived from GBD2016.[2] Tobacco smoking was defined as the population prevalence of using any type or quantity of smoked tobacco product on a daily basis.[3] Smoking prevalence data were obtained for males and females aged 10 – 24 years between 1990–2015 in five-year intervals and 2016.

Binge drinking was the indicator of risky alcohol use, defined as the proportion of 15 - 19-year olds who reported a binge (≥48 grams of alcohol for females and ≥60 grams for males on a single occasion) in the preceding 12 months. This indicator was defined for 15 – 19 year olds specifically in the Commission; while early initiation of alcohol use affects neurocognitive development and future patterns of substance use, binge drinking at 15 – 19 years more closely relates to acute harm and is also harmonised with data availability.[1] The available data differed from the originally proposed indicator in the sex-specific definition of a binge and the time period it referred to (12 months vs.

30 days). Prevalence was estimated for males and females aged 15 - 19 years between 1990–2015 in five-year intervals and for 2016.

Data relating to overweight and obesity were sourced from GBD2016.[4] Overweight was defined using International Obesity Task Force (IOTF) age and sex-specific thresholds, equivalent to BMI ≥ 25kg/m^2 at age 18 years.[5] Prevalence estimates of overweight for adolescents derived from IOTF thresholds are typically lower than those derived using WHO thresholds.[5] The indicator of overweight included individuals who were obese. Prevalence of overweight was obtained for males and females aged 10 – 24 years between 1990–2015 in five-year intervals and 2016.

The Commission recommended iron deficiency anaemia (IDA) as an indicator of undernutrition. Although estimates of IDA were available from GBD2016, we have chosen to report the prevalence of all-cause anaemia due to the limited quality of primary data for IDA. GBD estimates the burden of IDA by first estimating the burden of anaemia. Known causes of anaemia are then assigned (including hookworm, schistosomiasis, HIV, malaria, other infections and neglected tropical diseases, maternal haemorrhage, gynaecologic disorders, gastrointestinal bleeding, chronic kidney disease, and haemoglobinopathies, and malaria). The remaining unassigned burden of anaemia is then redistributed to other unaccounted causes, including IDA.[6] As such, the IDA estimates in GBD have high uncertainty. The definition of anaemia in GBD2016 is similar to WHO thresholds. For 10 - 14 year olds, anaemia was defined as haemoglobin (g/L) <115 for males and females (WHO <120).[7] For 15 – 24 year olds, anaemia was defined as haemoglobin <130 for males, <120 for non-pregnant females, and <110 for pregnant females.[6] The prevalence of anaemia was obtained for males and females aged 10 – 24 years between 1990–2015 in five-year intervals and 2016.

Social and structural determinants

We selected education data from the Barro-Lee dataset which provides educational attainment data for 146 countries from 1950 to 2010.[8] This dataset includes attainment data, measured in censuses and compiled by UNESCO, and estimates missing data using enrolment and population data. This dataset provided estimates of the prevalence of secondary school completion. The definition of secondary school completion was consistent with that of UNESCO: completion of secondary education most typically signified 12 or more years of education and prepared students for employment and/or tertiary education.[9] We reported this indicator for 20 – 24 year olds only as younger adolescents may not have completed secondary schooling. The Barro-Lee dataset is based on census data and modelled to provide complete estimates for 146 countries and territories. Three locations in the Barro-Lee dataset (Reunion, Hong Kong and Macao) were not otherwise included as countries in this analysis and were therefore excluded. Data were obtained for males and females aged 20 – 24 years for 143 countries from 1990 to 2010 (estimates more recent than 2010 were not available).

Data on the rate of adolescents not engaged in education, employment or training (NEET) were available from the International Labour Organization (ILO), compiled from labour force and other nationally representative surveys.[10] Data were available for 15 – 24 year olds rather than 20 – 24 year olds as defined in the 2016 Commission report (data for 10 – 14 year olds globally are limited). Data were available for 134 locations, however 10 of these were not

otherwise included in this analysis: Aruba, Falklands, Guernsey, Hong Kong , Kosovo, Macau, Netherlands Antilles, New Caledonia, Palau, and Reunion. Data for Kazakhstan were not sex-disaggregated and were also excluded. The 123 included countries had an estimate between 2007–2016, with some countries also having time-series data available (Appendix 2).

Data on adolescent live births were sourced from GBD2016. The Commission indicator was defined for 10 – 19 year olds, a definition that aligns with indicator 3.7.2. of the SDGs (adolescent birth rate for 10 – 14 year olds and 15 – 19 year olds, reported separately).[11] However, data quality differed significantly by age. GBD estimates for adolescent birth rates for 15 – 19 year olds are modelled from United Nations World Population Prospects, while estimates for 10 – 14 year olds are extrapolated based on observed ratios and adjusted for a pooled average age of menarche. In most countries, fertility amongst 10 – 14 year olds is very low and, given that estimates are extrapolated, the inclusion of 10 – 14 year olds in the definition largely dilutes all estimates by a similar factor and introduces measurement error. We therefore report the rate of adolescent live births per 1000 for 15 - 19 year olds, a more widely recognized indicator with better quality data. Data were obtained for 15 – 19 year old females for 195 countries from 1990 to 2016.

Child marriage data were generally limited in availability to those countries with household surveys and where child marriage tends to be more prevalent. Data on child marriage were drawn from a country-level dataset compiled by UNICEF, primarily drawing from available Demographic Health Surveys (DHS) and Multiple Indicator Cluster Surveys (MICS).[12] We included recent estimates for Paraguay (2016) and Nigeria (2017) that were not in the UNICEF dataset. Child marriage was defined as the proportion of 20 – 24 year old females reporting having been married or in union before the age of 18 years. Only a single most recent estimate was accessed for each country and we only included data from surveys between 2003 – 2016 (122 countries); 102 of these were sampled between 2010 – 2017. Child marriage data for two Pacific islands (Nauru and Tuvalu) were not otherwise included in the 195 countries of this analysis and excluded, resulting in data for 120 countries.

The Commission recommended that the indicator of unmet need for contraception function as a headline indicator of responsive health services. We reported on 'demand for modern contraception satisfied' (demand satisfied) in preference to unmet need, given that it is an SDG indicator, that it emphasises the importance of satisfying individuals' own choices around family planning, and that it prioritises the use of modern methods of contraception.[13] Demand satisfied is the proportion who use modern contraceptives divided by the total demand for contraception. Total demand for contraception is defined as the proportion of women using any contraceptive (modern or traditional) plus the proportion of women with an unmet need for contraception (want to space or limit births but are not using any contraceptive method).[13] Modern contraception was defined as male or female condoms, oral contraceptive, spermicide foam/jelly, diaphragms, implants, injections, use of an intrauterine device, or male or female sterilization. Traditional methods included withdrawal, period abstinence, rhythm method, and lactational amenorrhea method. Data for demand satisfied have previously been modelled by IHME for 15 – 49 year

olds, and are provided here for 15-24 using the same methods.[14] Data were obtained for 15 – 24 year old females for 195 countries, from 1990 to 2016.

Sociodemographic index

We included the IHME socio-demographic index (SDI) as an additional country-level indicator to provide context around the level of socio-economic development (reported in appendix 4).[15] The SDI is derived from three subscales: income per capita, average educational attainment for ages 15 or older, and the total fertility rate. Individual countries are scaled for each component of the SDI from 0 (worst value observed between 1980 - 2016) to 1 (best), with the SDI calculated as the geometric mean of the three rescaled components.

References

1. Patton GC, Sawyer SM, Santelli JS, et al. Our future: a Lancet commission on adolescent health and wellbeing. *Lancet* 2016; **387**(10036): 2423-78.
2. G. B. D. Risk Factors Collaborators. Global, regional, and national comparative risk assessment of 84 behavioural, environmental and occupational, and metabolic risks or clusters of risks, 1990-2016: a systematic analysis for the Global Burden of Disease Study 2016. *Lancet* 2017; **390**(10100): 1345-422.
3. GBD Tobacco Collaborators. Smoking prevalence and attributable disease burden in 195 countries and territories, 1990-2015: a systematic analysis from the Global Burden of Disease Study 2015. *Lancet* 2017.
4. GBD 2015 Risk Factor Collaborators. Global, regional, and national comparative risk assessment of 79 behavioural, environmental and occupational, and metabolic risks or clusters of risks, 1990–2015: a systematic analysis for the Global Burden of Disease Study 2015. *Lancet* 2016; **388**: 1659-724.
5. Cole TJ, Lobstein T. Extended international (IOTF) body mass index cut-offs for thinness, overweight and obesity. *Pediatric obesity* 2012; **7**(4): 284-94.
6. Kassebaum NJ, G. B. D. Anemia Collaborators. The Global Burden of Anemia. *Hematol Oncol Clin North Am* 2016; **30**(2): 247-308.
7. World Health Organization. Haemoglobin concentrations for the diagnosis of anaemia and assessment of severity. Geneva: WHO, 2011.
8. Barro RJ, Lee JW. A new data set of educational attainment in the world, 1950–2010. *Journal of development economics* 2013; **104**: 184-98.
9. United Nations Educational Scientific and Cultural Organization. International Standard Classification of Education (ISCED 2011). Montreal, Canada: UNESCO INstitute for Statistics, 2012.
10. International Labour Organization. Youth NEET rate. 2017. http://www.ilo.org/ilostat/faces/oracle/webcenter/portalapp/pagehierarchy/Page3.jspx?MBI_ID=20 (accessed January 2018.
11. Inter-Agency and Expert Group on SDG Indicators. Revised list of global Sustainable Development Goal Indicators, 2017.
12. UNICEF. Child marriage global database. 2017.
13. Fabic MS, Choi Y, Bongaarts J, et al. Meeting demand for family planning within a generation: the post-2015 agenda. *The Lancet* 2015; **385**(9981): 1928-31.
14. GBD SDG Collaborators. Measuring progress and projecting attainment on the basis of past trends of the health-related Sustainable Development Goals in 188 countries: an analysis from the Global Burden of Disease Study 2016. *Lancet* 2017; **390**(10100): 1423-59.
15. GBD SDG Collaborators. Measuring the health-related Sustainable Development Goals in 188 countries: a baseline analysis from the Global Burden of Disease Study 2015. *Lancet* 2016.

Appendix 2 – Data availability for NEET and Child Marriage

The data on Child Marriage is compiled by and available from the UNICEF database: https://data.unicef.org/wp-content/uploads/2015/12/Child-marriage-database_Nov-2017.xlsx
We updated estimates for child marriage for Paraguay and Nigeria using more recently available survey data: http://mics.unicef.org/surveys
Data for Not engaged in education, employment or training (NEET) was drawn from the International Labour Organization (ILO):
http://www.ilo.org/ilostat/faces/oracle/webcenter/portalapp/pagehierarchy/Page3.jspx?MBI_ID=20

	Most recent year of data		
**Country**	**Child Marriage**	**NEET**	Other years of data: **NEET**
Afghanistan	2015	-	
Albania	2008	2013	2008;2011;2012
Algeria	2012	2015	2009;2010;2011;2012;2013;2014
American Samoa	-	-	
Andorra	-	-	
Angola	2015	2011	2004
Antigua and Barbuda	-	-	
Argentina	-	2014	2004;2005;2006;2008;2009;2010;2011;2012;2013
Armenia	2015	2015	2011;2012;2013;2014
Australia	-	2015	2009;2010;2011;2012;2013;2014
Austria	-	2016	2004;2005;2006;2007;2008;2009;2010;2011;2012;2013;2014;2015
Azerbaijan	2011	-	
Bahrain	-	-	
Bangladesh	2014	2015	2005;2010;2013
Barbados	2012	-	
Belarus	2012	2009	
Belgium	-	2016	2004;2005;2006;2007;2008;2009;2010;2011;2012;2013;2014;2015
Belize	2015	2016	2010;2012;2015
Benin	2014	2012	2010;2011
Bermuda	-	2010	
Bhutan	2010	-	
Bolivia	2008	2015	2005;2006;2007;2008;2009;2011;2012;2013;2014
Bosnia and Herzegovina	2012	2016	2014;2015
Botswana	-	2009	
Brazil	2006	2016	2009;2011;2012;2013;2014;2015
Brunei	-	2014	
Bulgaria	-	2016	2003;2004;2005;2006;2007;2008;2009;2010;2011;2012;2013;2014;2015
Burkina Faso	2010	-	
Burundi	2010	2014	
Cambodia	2014	2012	2008;2009
Cameroon	2014	2010	2007

Canada	-	2016	2000;2001;2002;2003;2004;2005;2006;2007;2008;2009;2010;2011;2012;2013;2014;2015
Cape Verde	2005	-	
Central African Republic	2010	-	
Chad	2014	-	
Chile	-	2014	2010;2011;2012;2013
China	-	-	
Colombia	2015	2016	2003;2005;2009;2010;2011;2012;2013;2014;2015
Comoros	2012	2004	
Congo	2011	2005	
Costa Rica	2011	2016	2010;2011;2012;2013;2014;2015
Cote d'Ivoire	2011	-	
Croatia	-	2016	2005;2006;2007;2008;2009;2010;2011;2012;2013;2014;2015
Cuba	2014	-	
Cyprus	-	2016	2003;2004;2005;2006;2007;2008;2009;2010;2011;2012;2013;2014;2015
Czech Republic	-	2016	2003;2004;2005;2006;2007;2008;2009;2010;2011;2012;2013;2014;2015
Democratic Republic of the Congo	2013	-	
Denmark	-	2016	2003;2004;2005;2006;2007;2008;2009;2010;2011;2012;2013;2014;2015
Djibouti	2006	-	
Dominica	-	-	
Dominican Republic	2014	2015	2008;2009;2010;2011;2012;2013;2014
Ecuador	2004	2016	2009;2010;2011;2012;2013;2014;2015
Egypt	2014	2015	2011;2012;2013
El Salvador	2014	2015	2009;2010;2012;2013
Equatorial Guinea	2011	-	
Eritrea	2010	-	
Estonia	-	2016	2003;2004;2005;2006;2007;2008;2009;2010;2011;2012;2013;2014;2015
Ethiopia	2016	2012	2011
Federated States of Micronesia	-	-	
Fiji	-	2014	
Finland	-	2016	2003;2004;2005;2006;2007;2008;2009;2010;2011;2012;2013;2014;2015
France	-	2016	2003;2004;2005;2006;2007;2008;2009;2010;2011;2012;2013;2014;2015
Gabon	2012	-	
Georgia	2010	-	
Germany	-	2016	2003;2004;2005;2006;2007;2008;2009;2010;2011;2012;2013;2014;2015
Ghana	2014	2013	

Greece	-	2016	2003;2004;2005;2006;2007;2008;2009;2010;2011;2012;2013;2014;2015
Greenland	-	-	
Grenada	-	-	
Guam	-	-	
Guatemala	2014	2016	2011;2012;2013;2014;2015
Guinea	2012	2002	
Guinea-Bissau	2014	-	
Guyana	2014	-	
Haiti	2012	-	
Honduras	2012	2016	2007;2009;2010;2012;2013;2014;2015
Hungary	-	2016	2004;2005;2006;2007;2008;2009;2010;2011;2012;2013;2014;2015
Iceland	-	2016	2003;2004;2005;2006;2007;2008;2009;2010;2011;2012;2013;2014;2015
India	2005	2010	2004
Indonesia	2013	2016	2008;2009;2010;2013;2015
Iran	2010	2010	2009
Iraq	2011	-	
Ireland	-	2016	2003;2004;2005;2006;2007;2008;2009;2010;2011;2012;2013;2014;2015
Israel	-	2016	2009;2010;2011;2012;2013;2014;2015
Italy	-	2016	2004;2005;2006;2007;2008;2009;2010;2011;2012;2013;2014;2015
Jamaica	2011	-	
Japan	-	2016	2009;2010;2012;2013;2014;2015
Jordan	2012	-	
Kazakhstan	2015	-	
Kenya	2014	-	
Kiribati	2009	-	
Kuwait	-	-	
Kyrgyzstan	2014	2016	2009;2010;2011;2012;2013;2014;2015
Laos	2011	2010	
Latvia	-	2016	2003;2004;2005;2006;2007;2008;2009;2010;2011;2012;2013;2014;2015
Lebanon	2009	2007	
Lesotho	2014	-	
Liberia	2013	2010	
Libya	-	-	
Lithuania	-	2016	2004;2005;2006;2007;2008;2009;2010;2011;2012;2013;2014;2015
Luxembourg	-	2016	2003;2004;2005;2006;2007;2008;2009;2010;2011;2012;2013;2014;2015
Macedonia	2011	2016	2006;2007;2008;2009;2010;2011;2012;2013;2014;2015
Madagascar	2012	2012	
Malawi	2015	-	

Malaysia	-	2015	2013;2014
Maldives	2009	2014	2010
Mali	2015	2015	2010;2014
Malta	-	2016	2004;2005;2006;2007;2008;2009;2010;2011;2012;2013;2014;2015
Marshall Islands	2007	-	
Mauritania	2011	-	
Mauritius	-	-	
Mexico	2015	2016	2005;2006;2007;2008;2009;2010;2011;2012;2013;2014;2015
Moldova	2012	2015	2009;2010;2011;2012;2013;2014
Mongolia	2013	2016	2006;2008;2009;2010;2011;2012;2013;2014;2015
Montenegro	2013	2015	
Morocco	2003	-	
Mozambique	2011	2012	
Myanmar	2015	2015	
Namibia	2013	2016	2014
Nepal	2014	2008	
Netherlands	-	2016	2003;2004;2005;2006;2007;2008;2009;2010;2011;2012;2013;2014;2015
New Zealand	-	2016	2007;2008;2009;2010;2011;2012;2013;2014;2015
Nicaragua	2006	2014	
Niger	2012	2011	
Nigeria	2017	2013	
North Korea	-	-	
Northern Mariana Islands	-	-	
Norway	-	2016	2003;2004;2005;2006;2007;2008;2009;2010;2011;2012;2013;2014;2015
Oman	-	-	
Pakistan	2012	2015	2010;2011;2013;2014
Palestine	2014	2015	2011;2012;2013
Panama	2013	2016	2009;2010;2011;2012;2014
Papua New Guinea	2006	2010	
Paraguay	2016	2015	2009;2010;2011;2012;2013;2014
Peru	2015	2015	2004;2005;2006;2007;2008;2009;2010;2011;2012;2013;2014
Philippines	2013	2016	2009;2010;2011;2012;2014;2015
Poland	-	2016	2004;2005;2006;2007;2008;2009;2010;2011;2012;2013;2014;2015
Portugal	-	2016	2004;2005;2006;2007;2008;2009;2010;2011;2012;2013;2014;2015
Puerto Rico	-	-	
Qatar	2012	2016	2009

Romania	-	2016	2003;2004;2005;2006;2007;2008;2009;2010;<br>2011;2012;2013;2014;2015
Russia	-	2015	2009;2010;2011;2012;2013;2014
Rwanda	2014	-	
Saint Lucia	2012	-	
Saint Vincent and the Grenadines	-	-	
Samoa	2014	2012	
Sao Tome and Principe	2014	-	
Saudi Arabia	-	2015	2009;2011;2012;2013;2014
Senegal	2015	-	
Serbia	2014	2016	2009;2010;2011;2012;2013;2014;2015
Seychelles	-	-	
Sierra Leone	2013	2014	
Singapore	-	2014	
Slovakia	-	2016	2003;2004;2005;2006;2007;2008;2009;2010;<br>2011;2012;2013;2014;2015
Slovenia	-	2016	2003;2004;2005;2006;2007;2008;2009;2010;<br>2011;2012;2013;2014;2015
Solomon Islands	2015	-	
Somalia	2006	-	
South Africa	2003	2016	2013;2014;2015
South Korea	-	-	
South Sudan	2010	-	
Spain	-	2016	2003;2004;2005;2006;2007;2008;2009;2010;<br>2011;2012;2013;2014;2015
Sri Lanka	2006	2014	2010;2013
Sudan	2014	-	
Suriname	2010	-	
Swaziland	2014	-	
Sweden	-	2016	2003;2004;2005;2006;2007;2008;2009;2010;<br>2011;2012;2013;2014;2015
Switzerland	-	2016	2003;2004;2005;2006;2007;2008;2009;2010;<br>2011;2012;2013;2014;2015
Syria	2006	-	
Taiwan	-	-	
Tajikistan	2012	-	
Tanzania	2015	2014	2005
Thailand	2015	2016	2009;2010;2011;2012;2013;2014;2015
The Bahamas	-	-	
The Gambia	2013	2012	
Timor-Leste	2009	2013	2010
Togo	2013	2011	
Tonga	2012	-	
Trinidad and Tobago	2006	2013	

Tunisia	2011	-	
Turkey	2013	2016	2006;2007;2008;2009;2010;2011;2012;2013;2014;2015
Turkmenistan	2015	-	
Uganda	2011	2013	2005;2009;2012
Ukraine	2012	2015	2014
United Arab Emirates	-	-	
United Kingdom	-	2016	2004;2005;2006;2007;2008;2009;2010;2011;2012;2013;2014;2015
United States	-	2012	2009;2010;2011
Uruguay	2013	2016	2009;2010;2011;2012;2013;2014;2015
Uzbekistan	2006	-	
Vanuatu	2013	-	
Venezuela	-	2010	2009
Vietnam	2014	2016	2012;2013;2014;2015
Virgin Islands, U.S.	-	-	
Yemen	2013	2014	
Zambia	2013	2008	
Zimbabwe	2015	-	

Appendix 3 – Uncertainty bounds for indicators:

Appendix 2.1 DALYs per 100 000 population due to group one conditions in 10 - 24 year olds

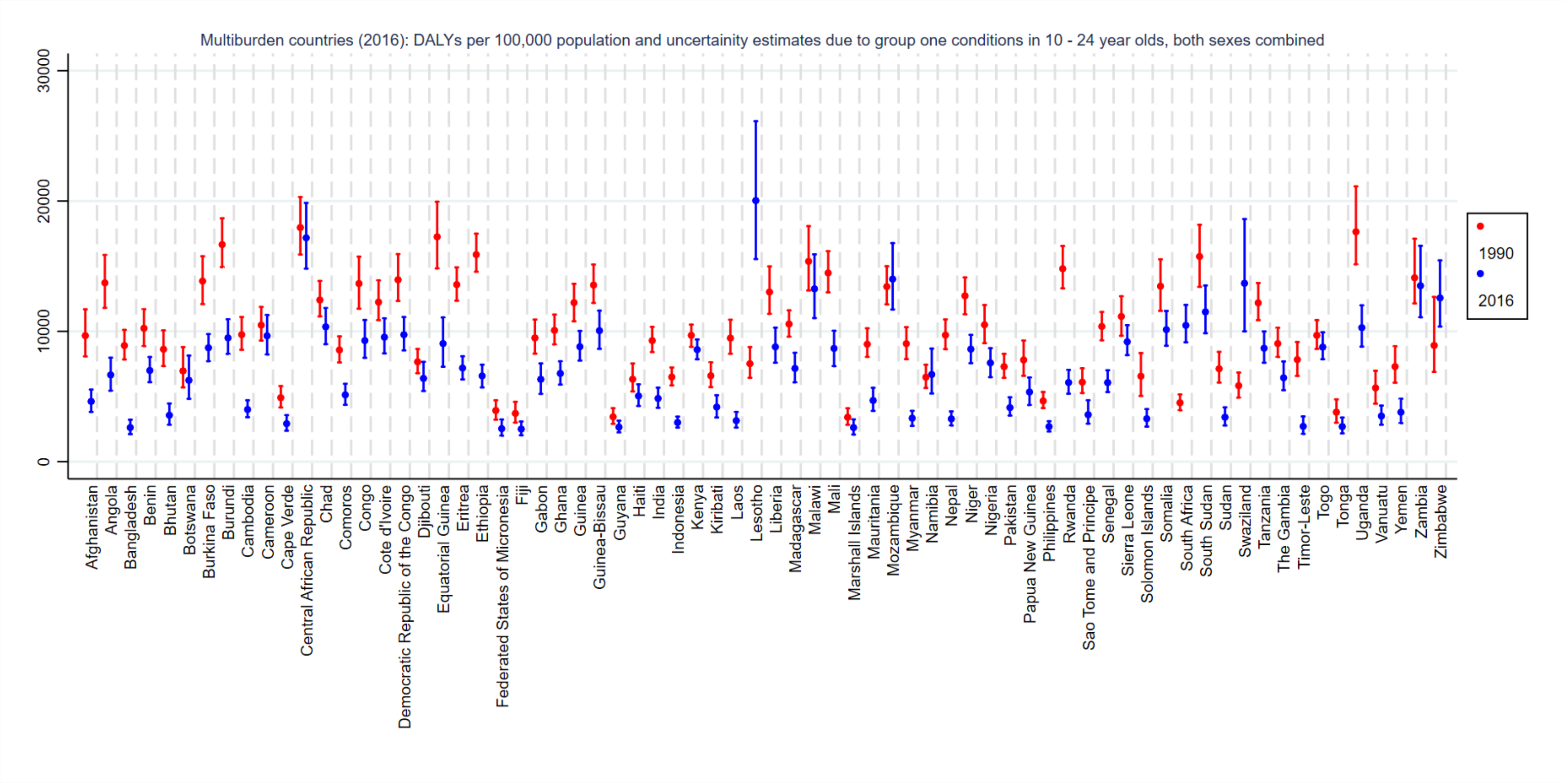

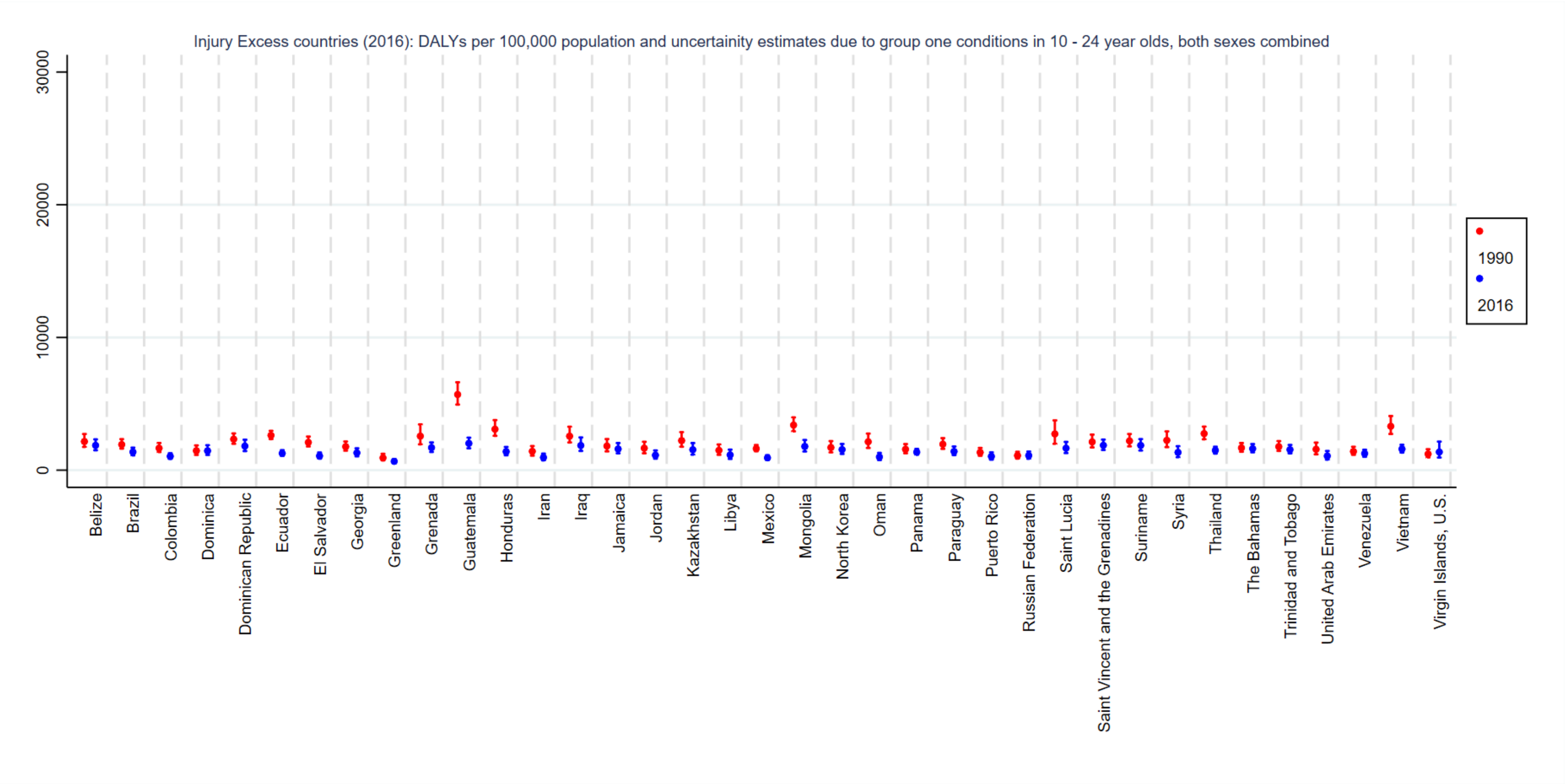
Injury Excess countries (2016): DALYs per 100,000 population and uncertainity estimates due to group one conditions in 10 - 24 year olds, both sexes combined
0
10000
20000
30000
1990
2016
Belize
Brazil
Colombia
Dominica
Dominican Republic
Ecuador
El Salvador
Georgia
Greenland
Grenada
Guatemala
Honduras
Iran
Iraq
Jamaica
Jordan
Kazakhstan
Libya
Mexico
Mongolia
North Korea
Oman
Panama
Paraguay
Puerto Rico
Russian Federation
Saint Lucia
Saint Vincent and the Grenadines
Suriname
Syria
Thailand
The Bahamas
Trinidad and Tobago
United Arab Emirates
Venezuela
Vietnam
Virgin Islands, U.S.

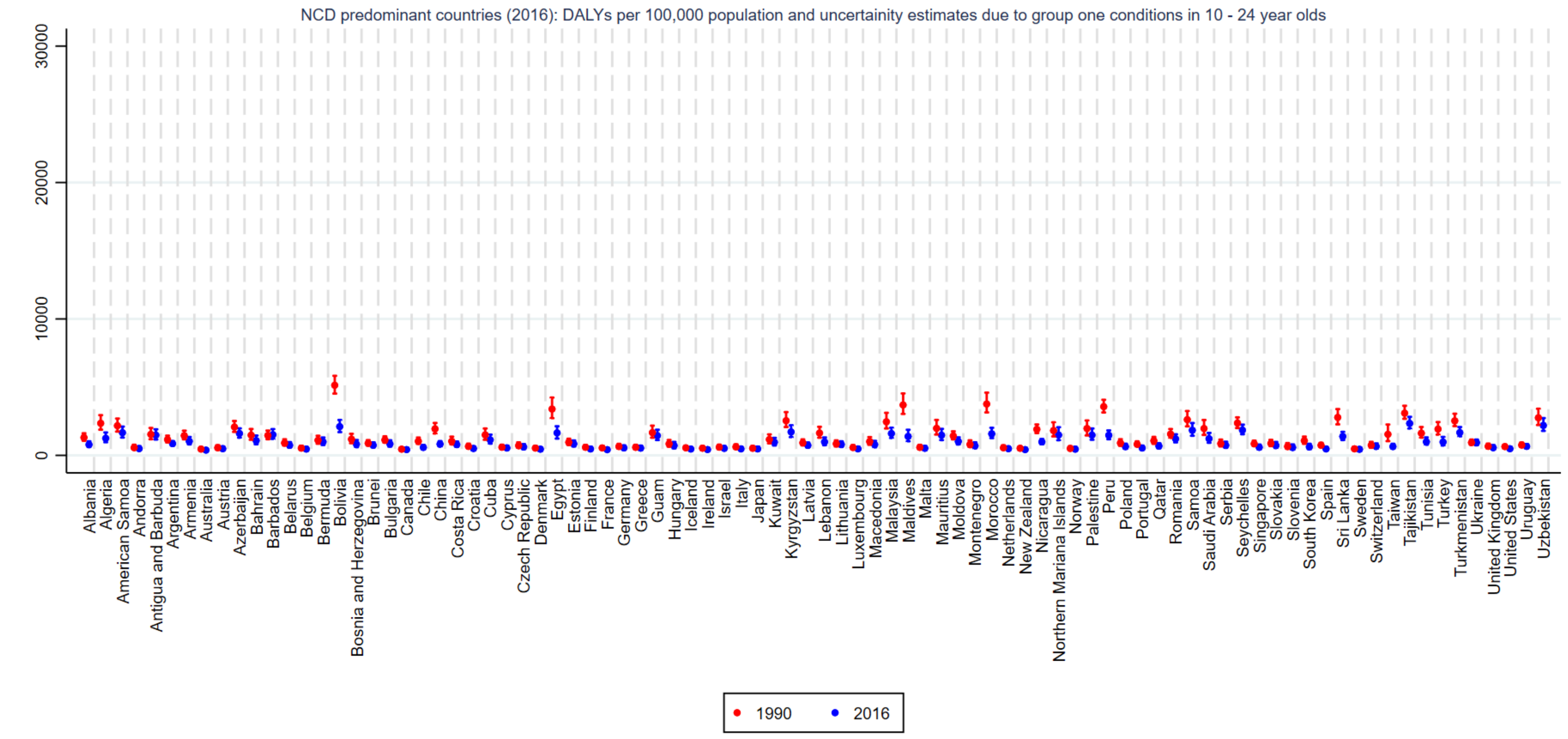
NCD predominant countries (2016): DALYs per 100,000 population and uncertainity estimates due to group one conditions in 10 - 24 year olds
30000
20000
10000
0
Albania
Algeria
American Samoa
Andorra
Antigua and Barbuda
Argentina
Armenia
Australia
Austria
Azerbaijan
Bahrain
Barbados
Belarus
Belgium
Bermuda
Bolivia
Bosnia and Herzegovina
Brunei
Bulgaria
Canada
Chile
China
Costa Rica
Croatia
Cuba
Cyprus
Czech Republic
Denmark
Egypt
Estonia
Finland
France
Germany
Greece
Guam
Hungary
Iceland
Ireland
Israel
Italy
Japan
Kuwait
Kyrgyzstan
Latvia
Lebanon
Lithuania
Luxembourg
Macedonia
Malaysia
Maldives
Malta
Mauritius
Moldova
Montenegro
Morocco
Netherlands
New Zealand
Nicaragua
Northern Mariana Islands
Norway
Palestine
Peru
Poland
Portugal
Qatar
Romania
Samoa
Saudi Arabia
Serbia
Seychelles
Singapore
Slovakia
Slovenia
South Korea
Spain
Sri Lanka
Sweden
Switzerland
Taiwan
Tajikistan
Tunisia
Turkey
Turkmenistan
Ukraine
United Kingdom
United States
Uruguay
Uzbekistan
1990
2016

Appendix 2.2 DALYs per 100 000 population due to injury and violence in 10 - 24 year olds

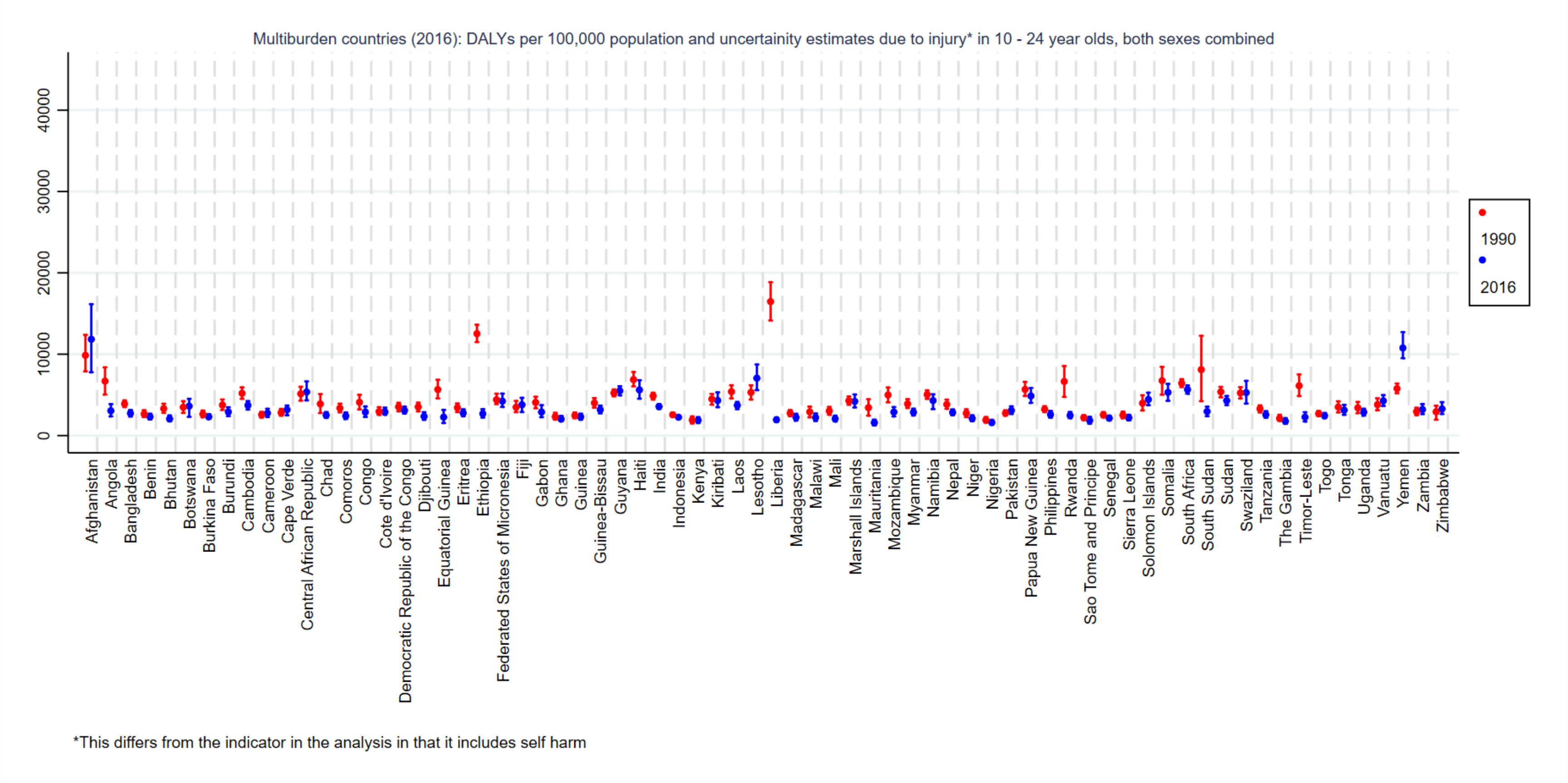

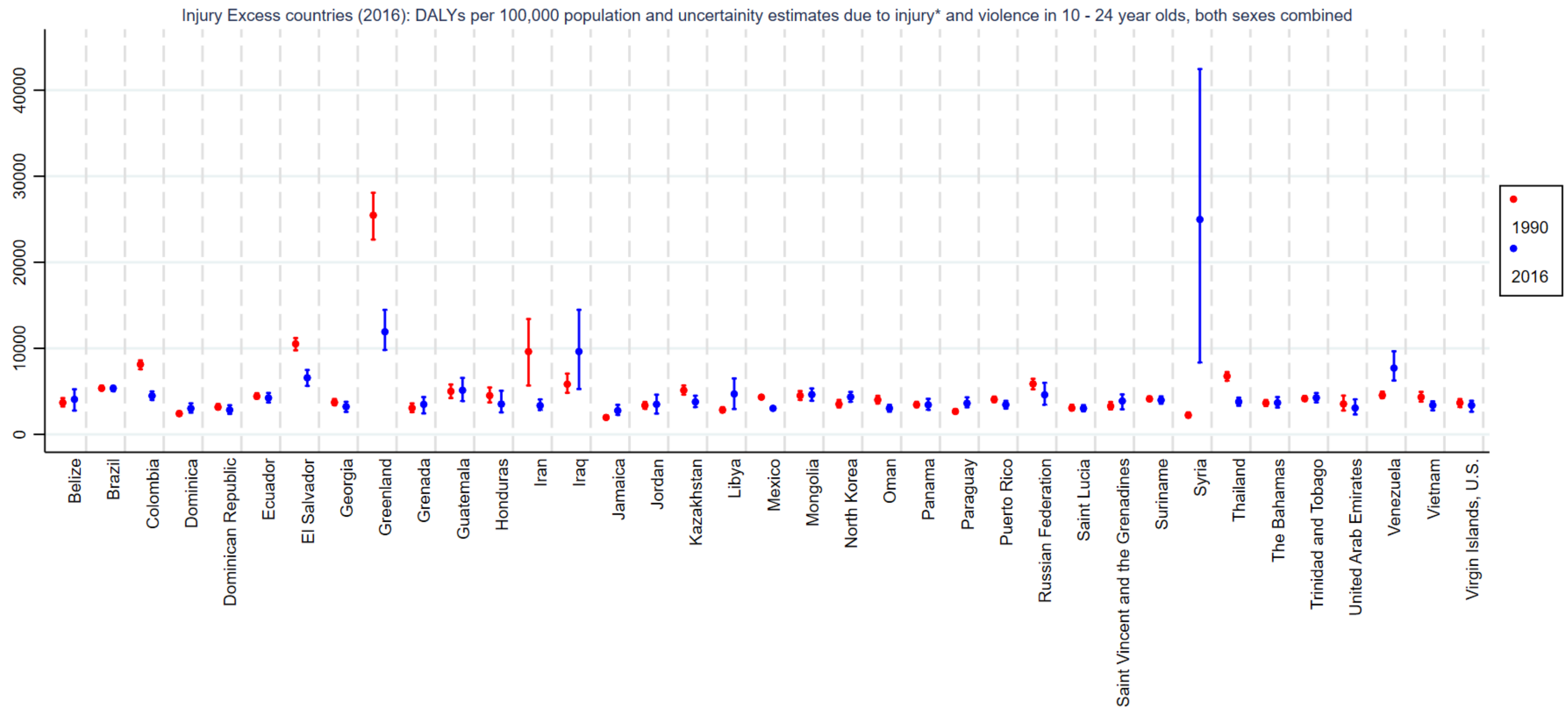

*This differs from the indicator in the paper as it includes self harm

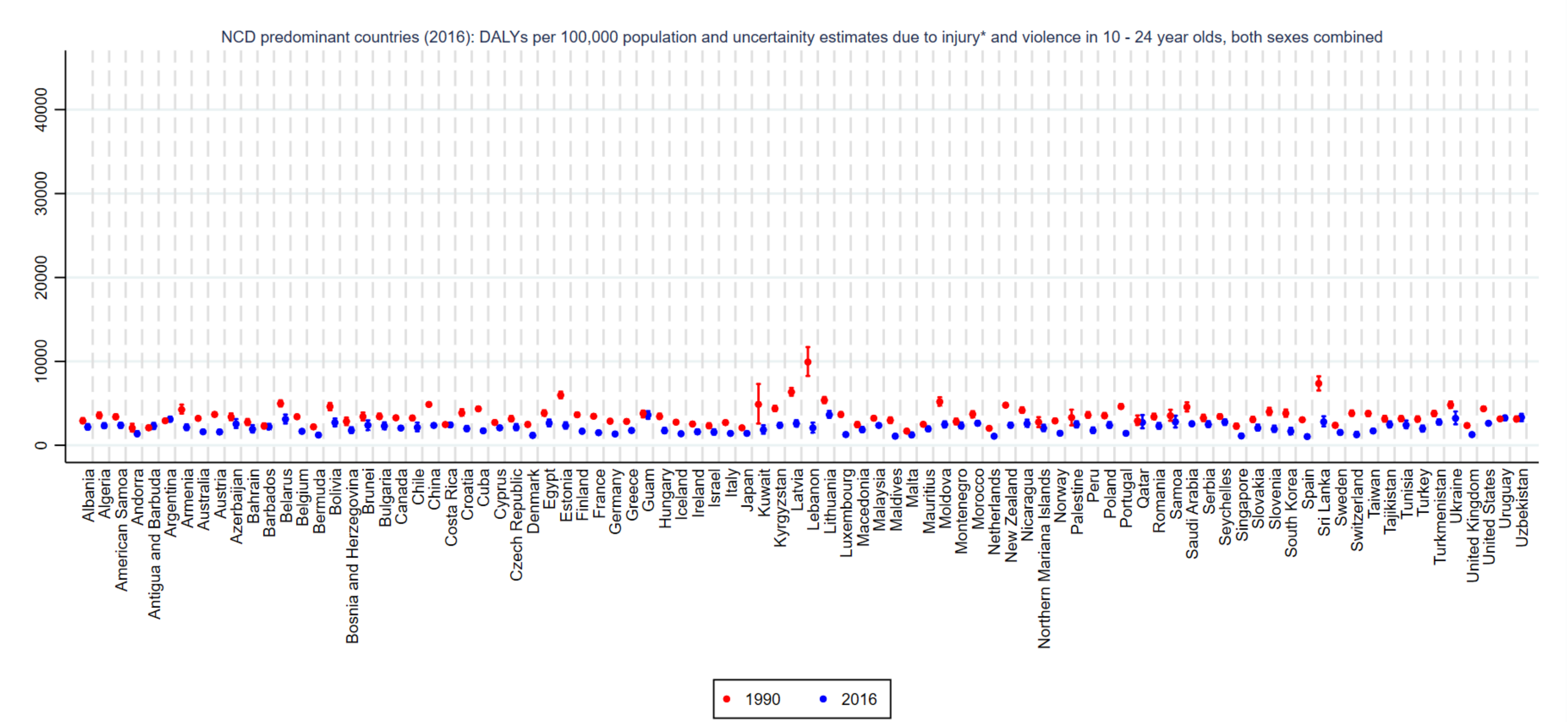

*This differs from the indicator in the paper as it includes self harm

Appendix 2.3 DALYs per 100 000 population due to non-communicable diseases in 10 - 24 year olds

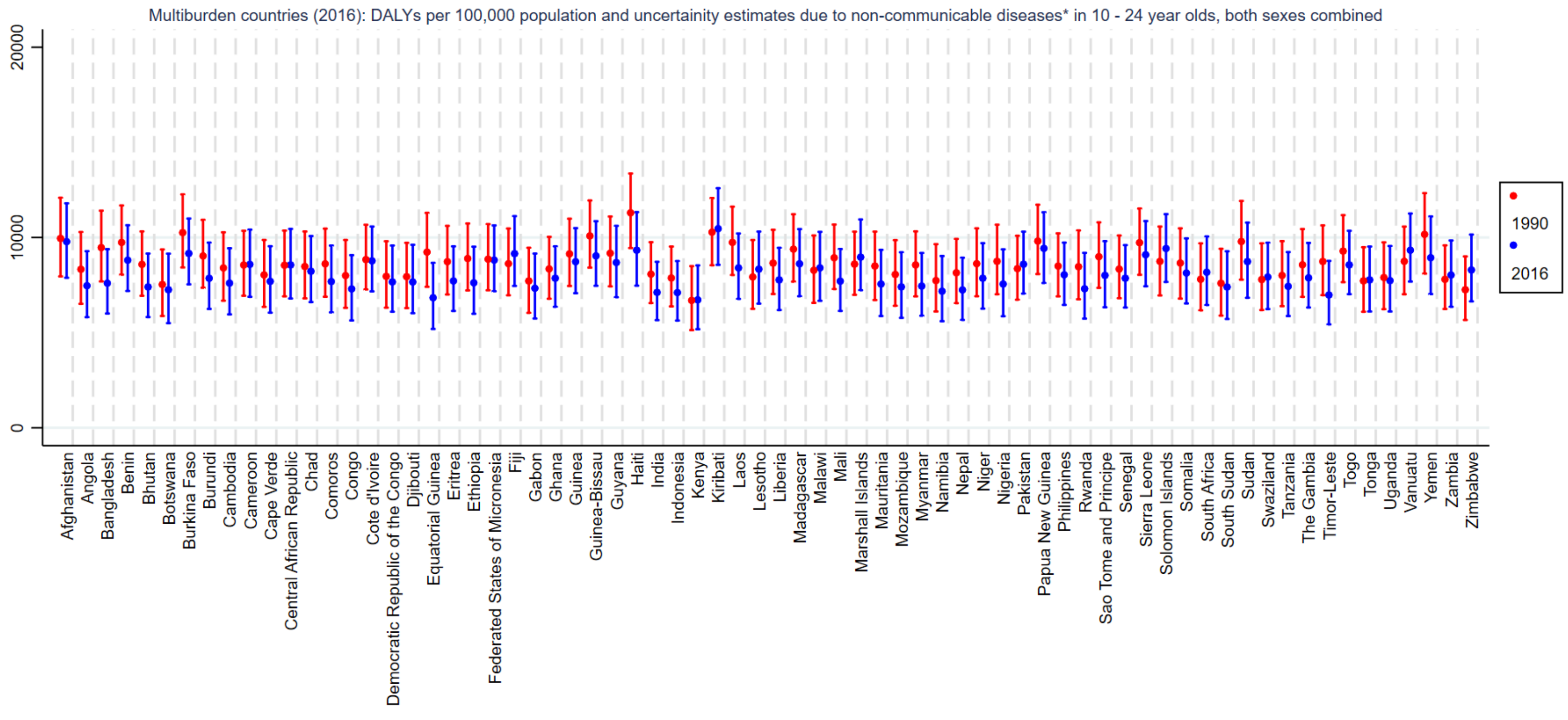

*This differs from the indicator in the analysis in that it does not include self harm

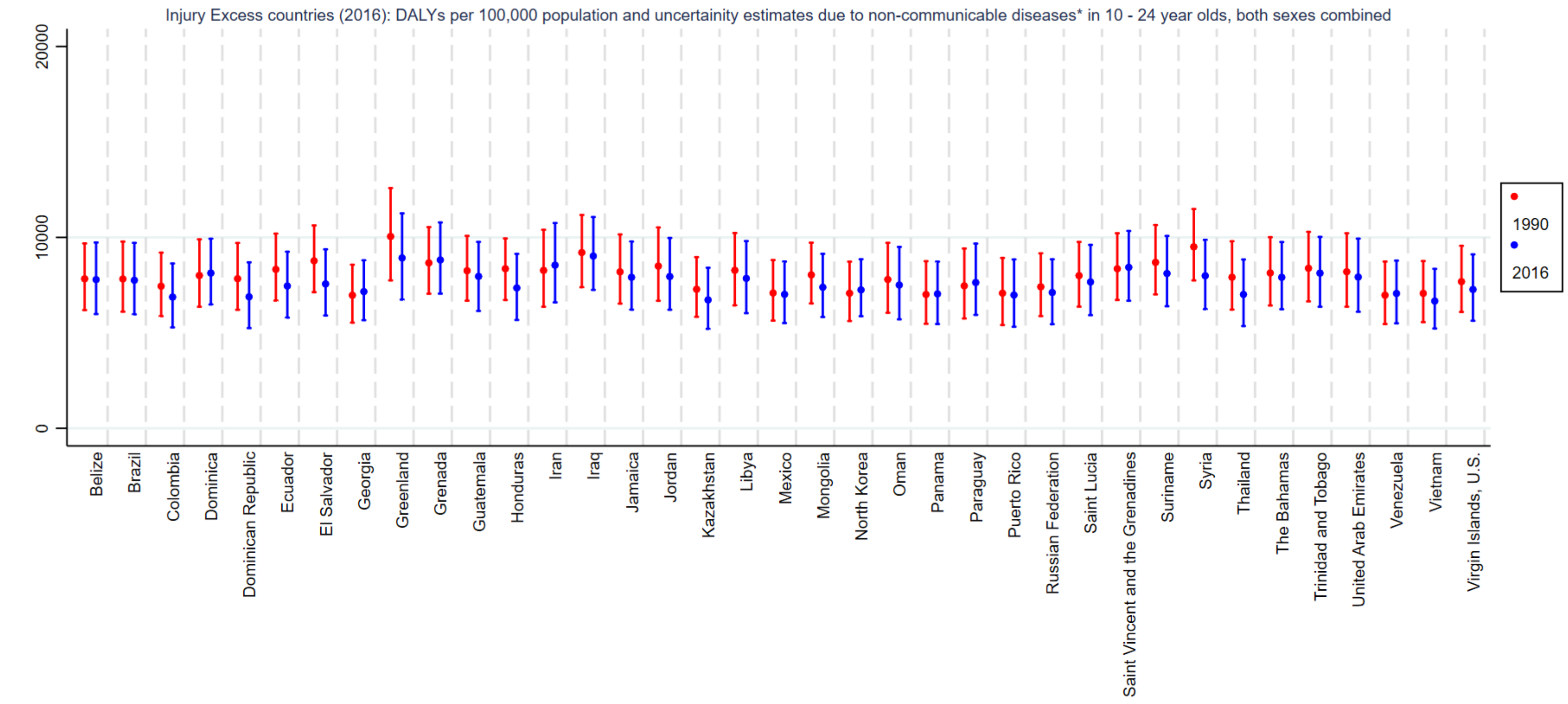

*This differs from the indicator in the paper as it does not include self harm

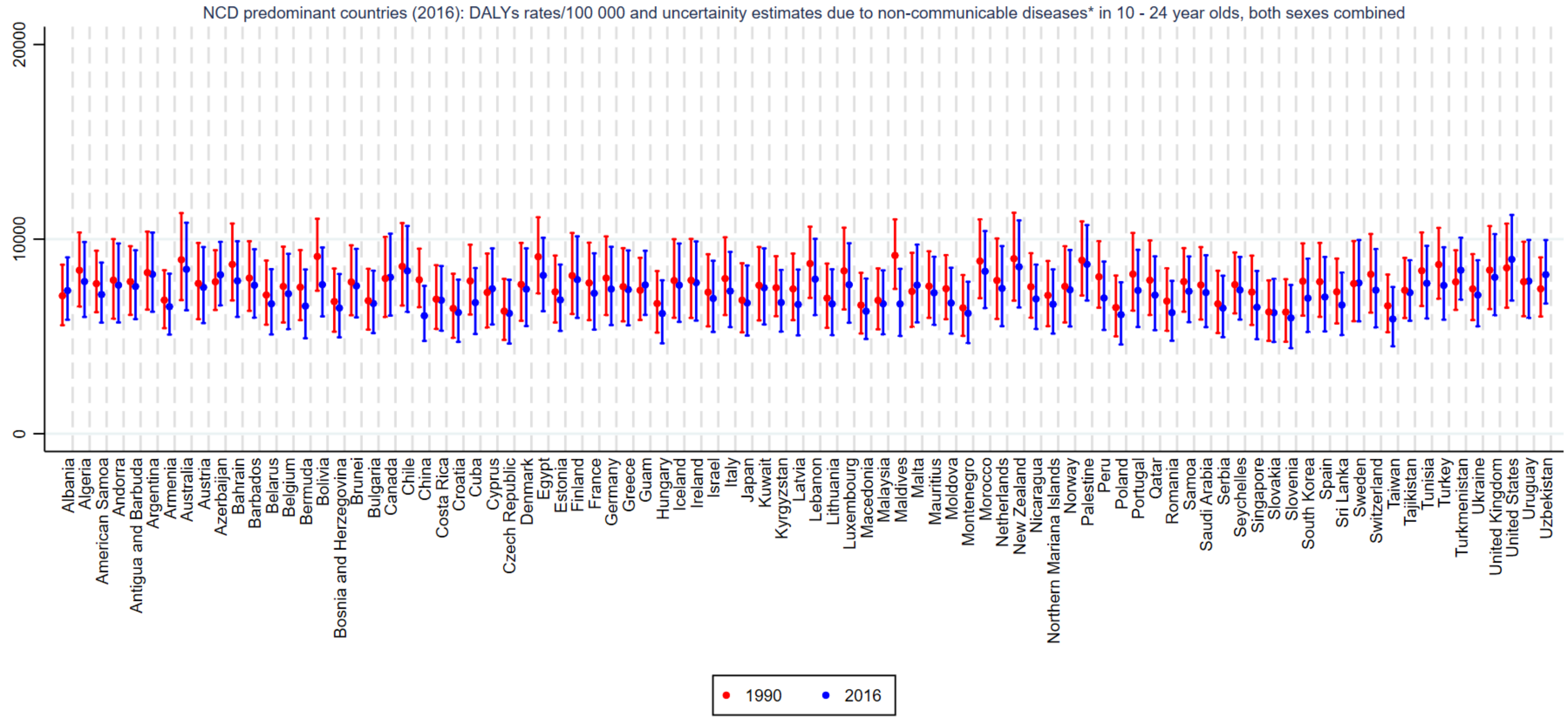

*This differs from the indicator in the paper as it does not include self harm

Appendix 2.4 DALYs per 100 000 population due to self harm in 10 - 24 year olds

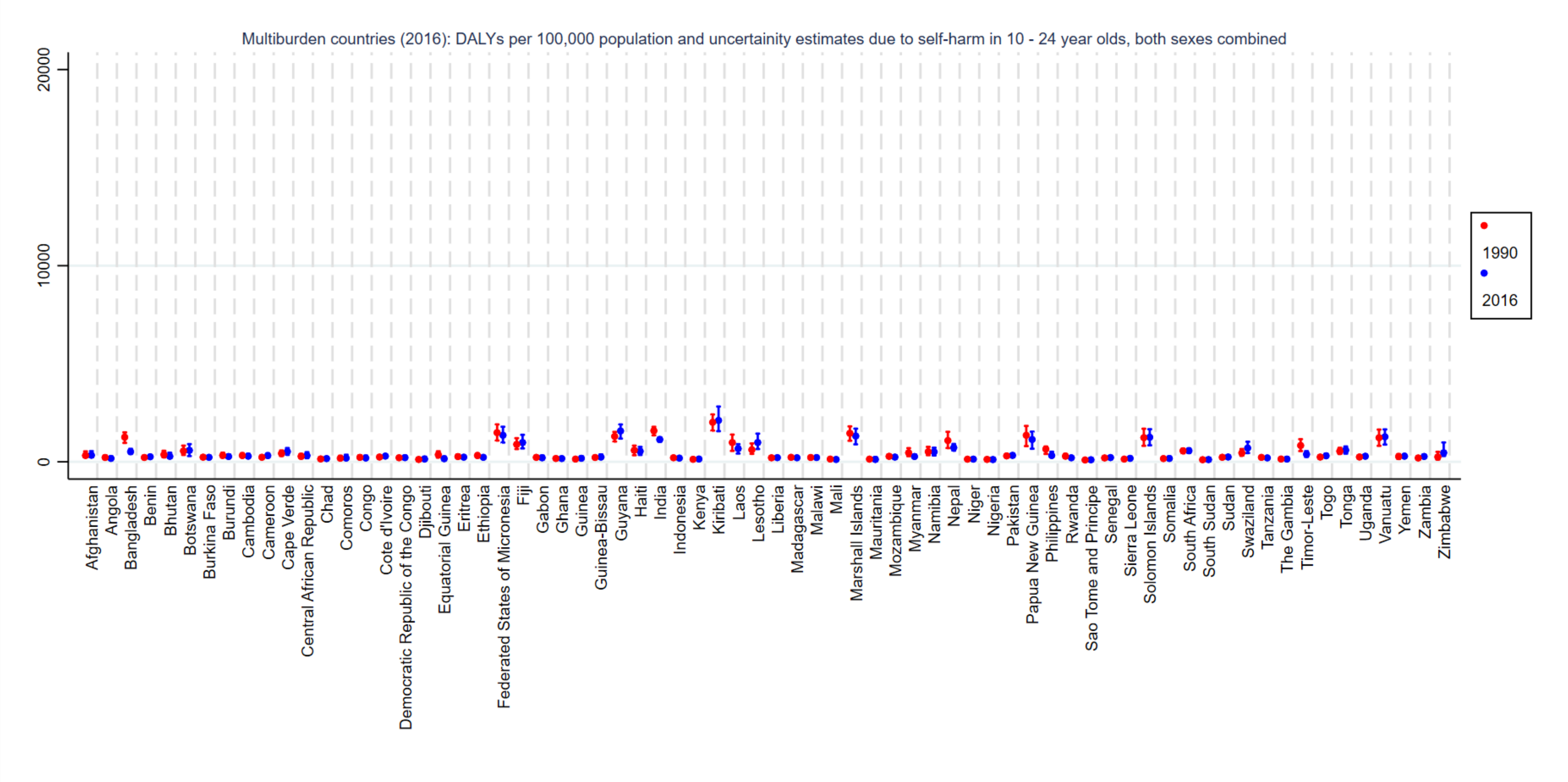

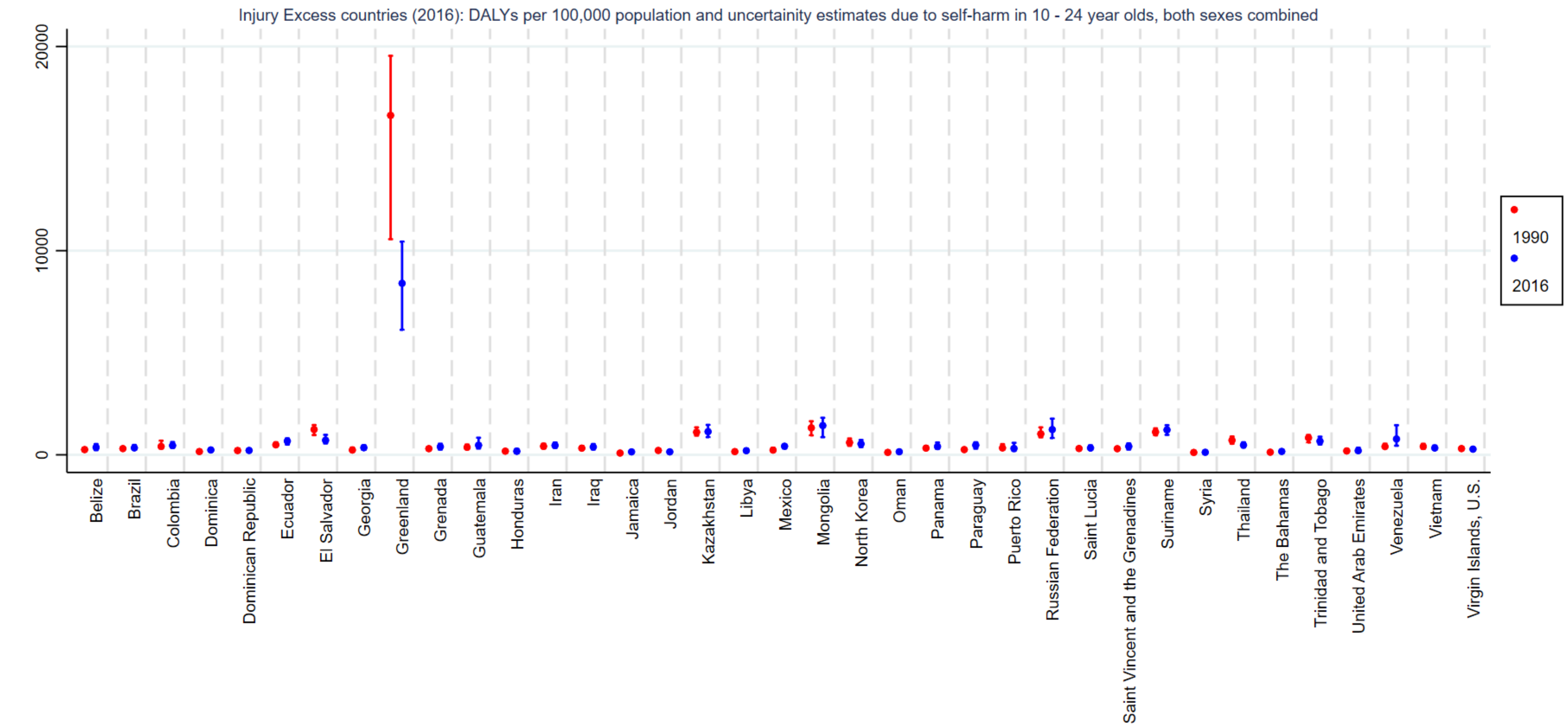
Injury Excess countries (2016): DALYs per 100,000 population and uncertainity estimates due to self-harm in 10 - 24 year olds, both sexes combined
20000
10000
0
Belize
Brazil
Colombia
Dominica
Dominican Republic
Ecuador
El Salvador
Georgia
Greenland
Grenada
Guatemala
Honduras
Iran
Iraq
Jamaica
Jordan
Kazakhstan
Libya
Mexico
Mongolia
North Korea
Oman
Panama
Paraguay
Puerto Rico
Russian Federation
Saint Lucia
Saint Vincent and the Grenadines
Suriname
Syria
Thailand
The Bahamas
Trinidad and Tobago
United Arab Emirates
Venezuela
Vietnam
Virgin Islands, U.S.
1990
2016

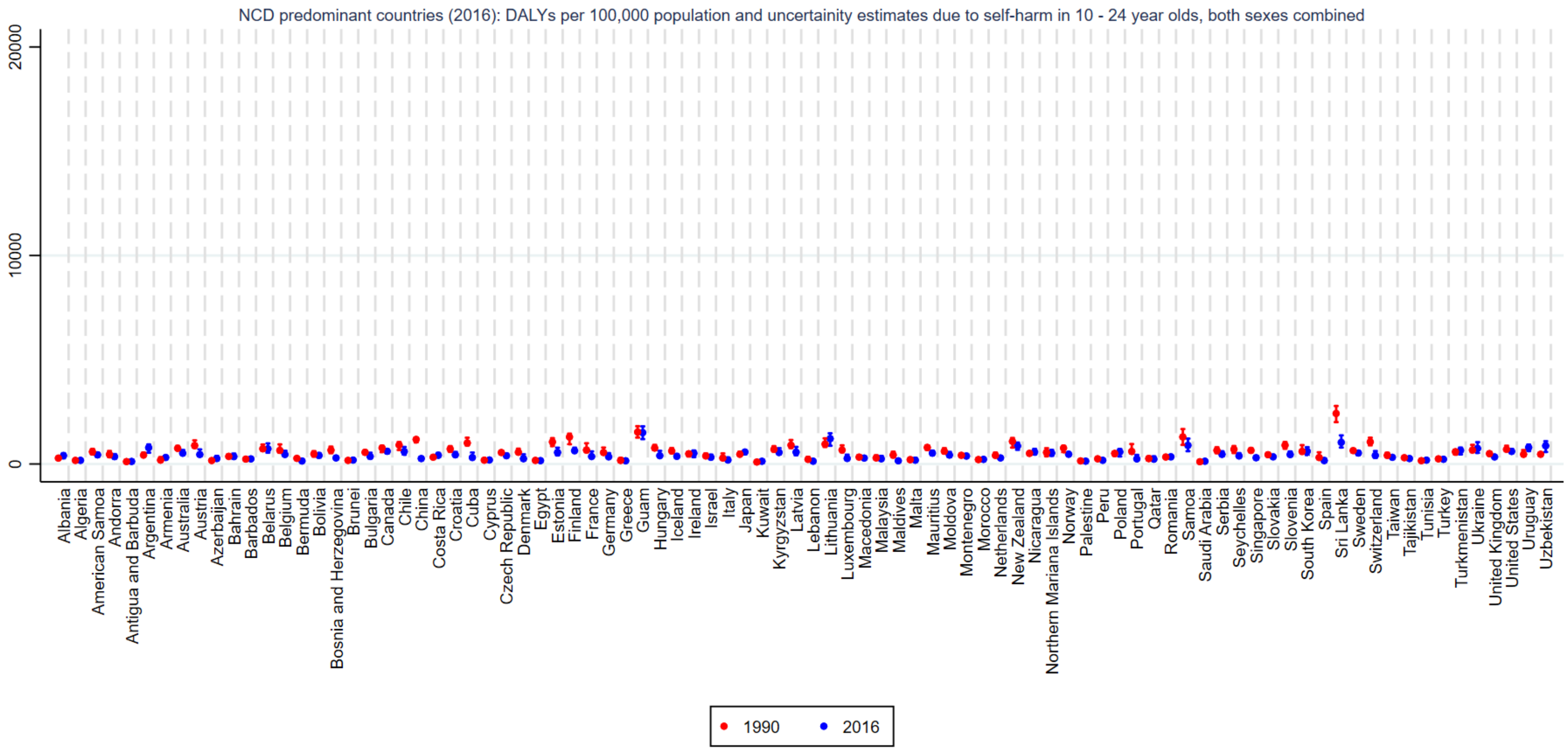

NCD predominant countries (2016): DALYs per 100,000 population and uncertainity estimates due to self-harm in 10 - 24 year olds, both sexes combined
20000
10000
0
Albania
Algeria
American Samoa
Andorra
Antigua and Barbuda
Argentina
Armenia
Australia
Austria
Azerbaijan
Bahrain
Barbados
Belarus
Belgium
Bermuda
Bolivia
Bosnia and Herzegovina
Brunei
Bulgaria
Canada
Chile
China
Costa Rica
Croatia
Cuba
Cyprus
Czech Republic
Denmark
Egypt
Estonia
Finland
France
Germany
Greece
Guam
Hungary
Iceland
Ireland
Israel
Italy
Japan
Kuwait
Kyrgyzstan
Latvia
Lebanon
Lithuania
Luxembourg
Macedonia
Malaysia
Maldives
Malta
Mauritius
Moldova
Montenegro
Morocco
Netherlands
New Zealand
Nicaragua
Northern Mariana Islands
Norway
Palestine
Peru
Poland
Portugal
Qatar
Romania
Samoa
Saudi Arabia
Serbia
Seychelles
Singapore
Slovakia
Slovenia
South Korea
Spain
Sri Lanka
Sweden
Switzerland
Taiwan
Tajikistan
Tunisia
Turkey
Turkmenistan
Ukraine
United Kingdom
United States
Uruguay
Uzbekistan
1990
2016

Appendix 2.5 Total DALYs per 100 000 population due to all causes in 10 - 24 year olds

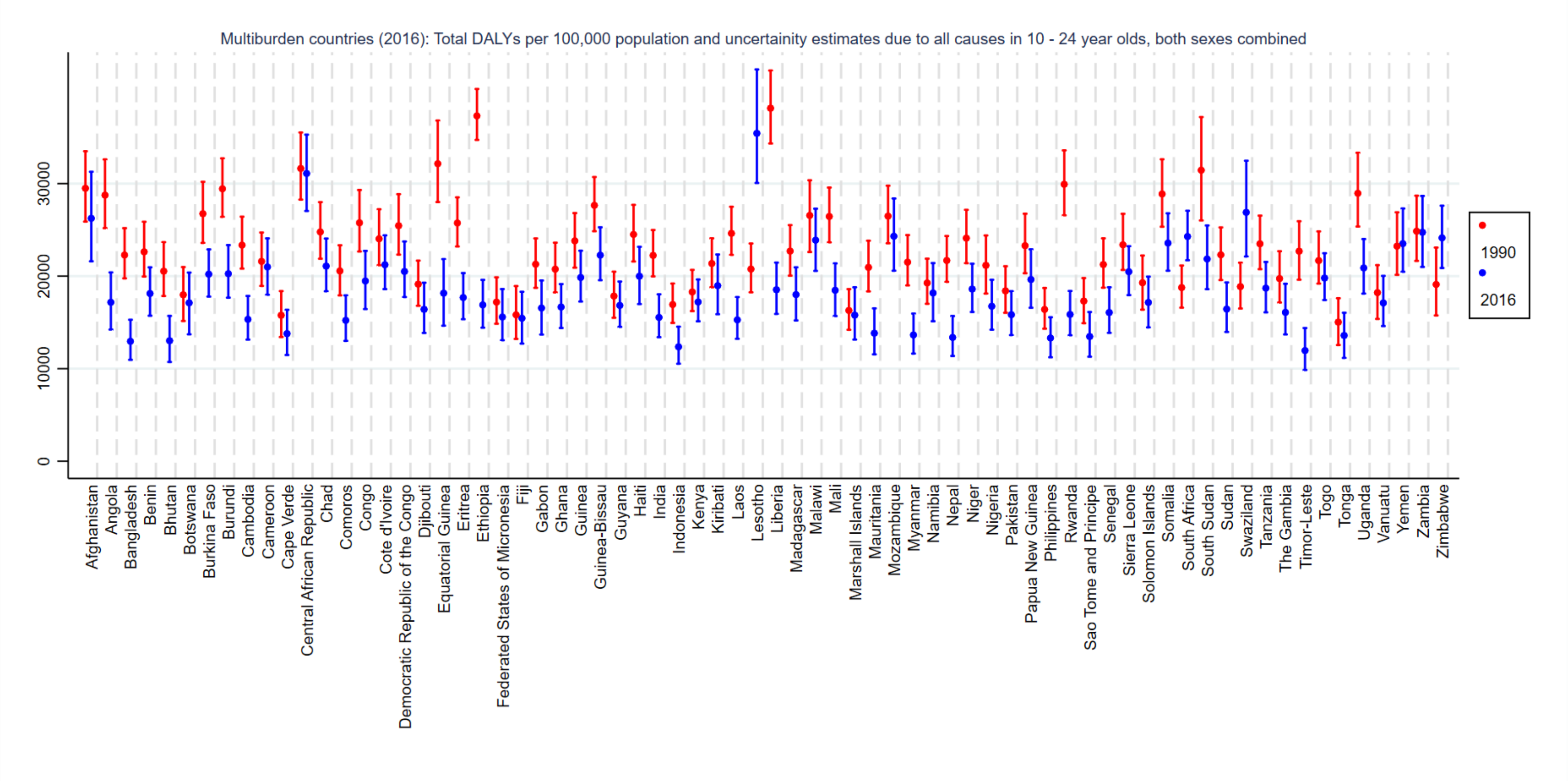

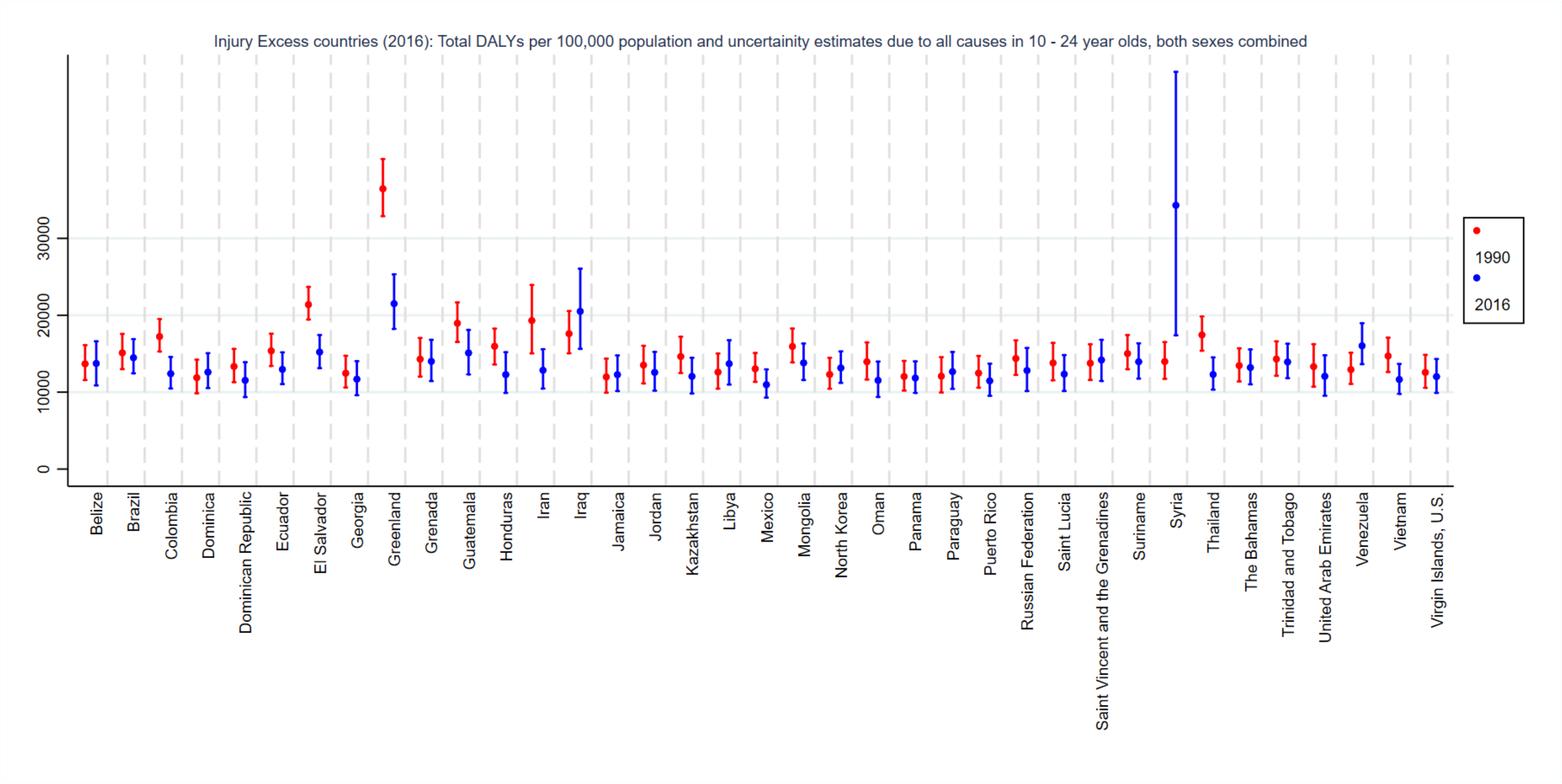

Injury Excess countries (2016): Total DALYs per 100,000 population and uncertainity estimates due to all causes in 10 - 24 year olds, both sexes combined
0
10000
20000
30000
Belize
Brazil
Colombia
Dominica
Dominican Republic
Ecuador
El Salvador
Georgia
Greenland
Grenada
Guatemala
Honduras
Iran
Iraq
Jamaica
Jordan
Kazakhstan
Libya
Mexico
Mongolia
North Korea
Oman
Panama
Paraguay
Puerto Rico
Russian Federation
Saint Lucia
Saint Vincent and the Grenadines
Suriname
Syria
Thailand
The Bahamas
Trinidad and Tobago
United Arab Emirates
Venezuela
Vietnam
Virgin Islands, U.S.
1990
2016

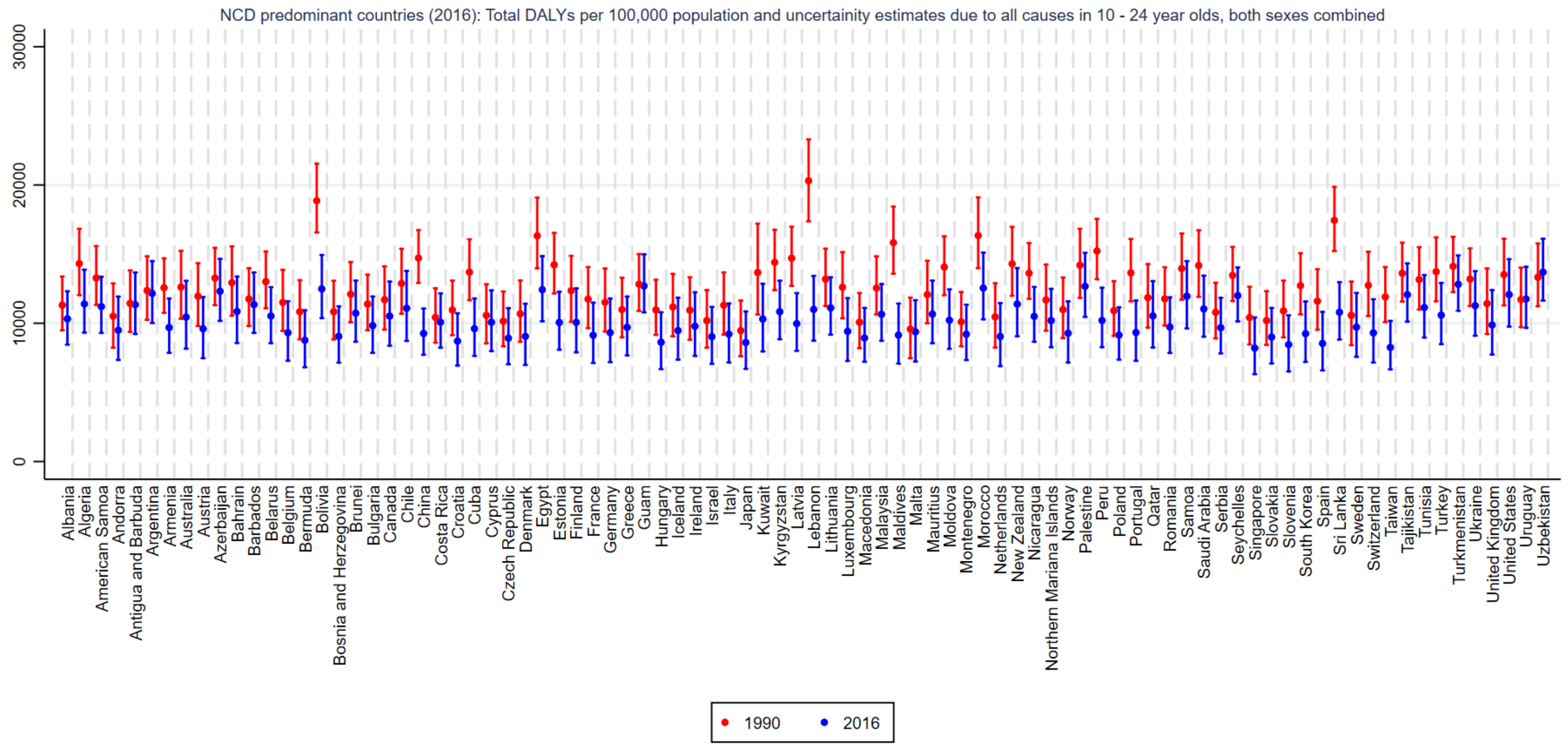
NCD predominant countries (2016): Total DALYs per 100,000 population and uncertainity estimates due to all causes in 10 - 24 year olds, both sexes combined
0
10000
20000
30000
Albania
Algeria
American Samoa
Andorra
Antigua and Barbuda
Argentina
Armenia
Australia
Austria
Azerbaijan
Bahrain
Barbados
Belarus
Belgium
Bermuda
Bolivia
Bosnia and Herzegovina
Brunei
Bulgaria
Canada
Chile
China
Costa Rica
Croatia
Cuba
Cyprus
Czech Republic
Denmark
Egypt
Estonia
Finland
France
Germany
Greece
Guam
Hungary
Iceland
Ireland
Israel
Italy
Japan
Kuwait
Kyrgyzstan
Latvia
Lebanon
Lithuania
Luxembourg
Macedonia
Malaysia
Maldives
Malta
Mauritius
Moldova
Montenegro
Morocco
Netherlands
New Zealand
Nicaragua
Northern Mariana Islands
Norway
Palestine
Peru
Poland
Portugal
Qatar
Romania
Samoa
Saudi Arabia
Serbia
Seychelles
Singapore
Slovakia
Slovenia
South Korea
Spain
Sri Lanka
Sweden
Switzerland
Taiwan
Tajikistan
Tunisia
Turkey
Turkmenistan
Ukraine
United Kingdom
United States
Uruguay
Uzbekistan
1990
2016

Appendix 2.6 Years of life lost (YLL) for all causes in 10 - 24 year olds

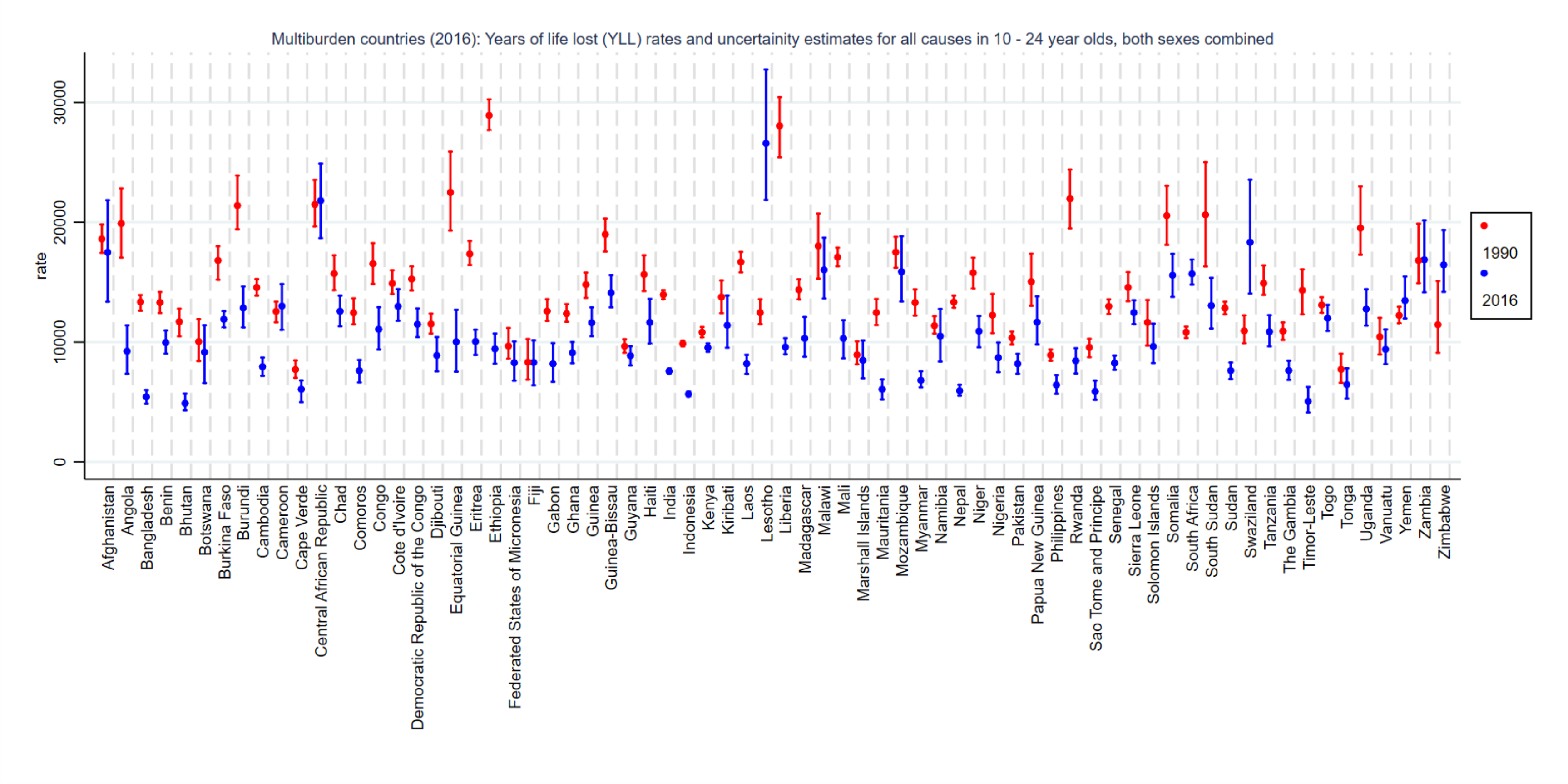

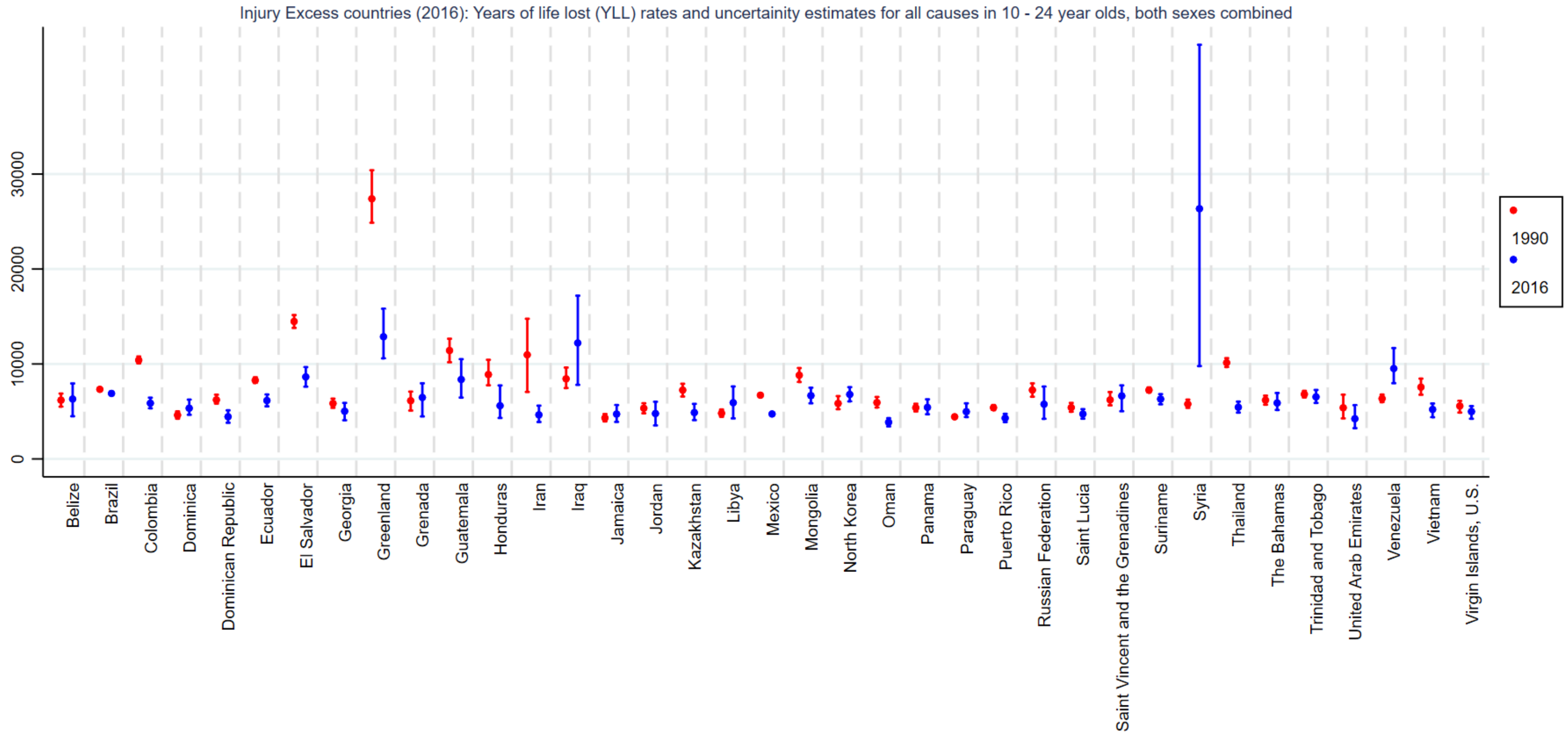
Injury Excess countries (2016): Years of life lost (YLL) rates and uncertainity estimates for all causes in 10 - 24 year olds, both sexes combined
0
10000
20000
30000
Belize
Brazil
Colombia
Dominica
Dominican Republic
Ecuador
El Salvador
Georgia
Greenland
Grenada
Guatemala
Honduras
Iran
Iraq
Jamaica
Jordan
Kazakhstan
Libya
Mexico
Mongolia
North Korea
Oman
Panama
Paraguay
Puerto Rico
Russian Federation
Saint Lucia
Saint Vincent and the Grenadines
Suriname
Syria
Thailand
The Bahamas
Trinidad and Tobago
United Arab Emirates
Venezuela
Vietnam
Virgin Islands, U.S.
1990
2016

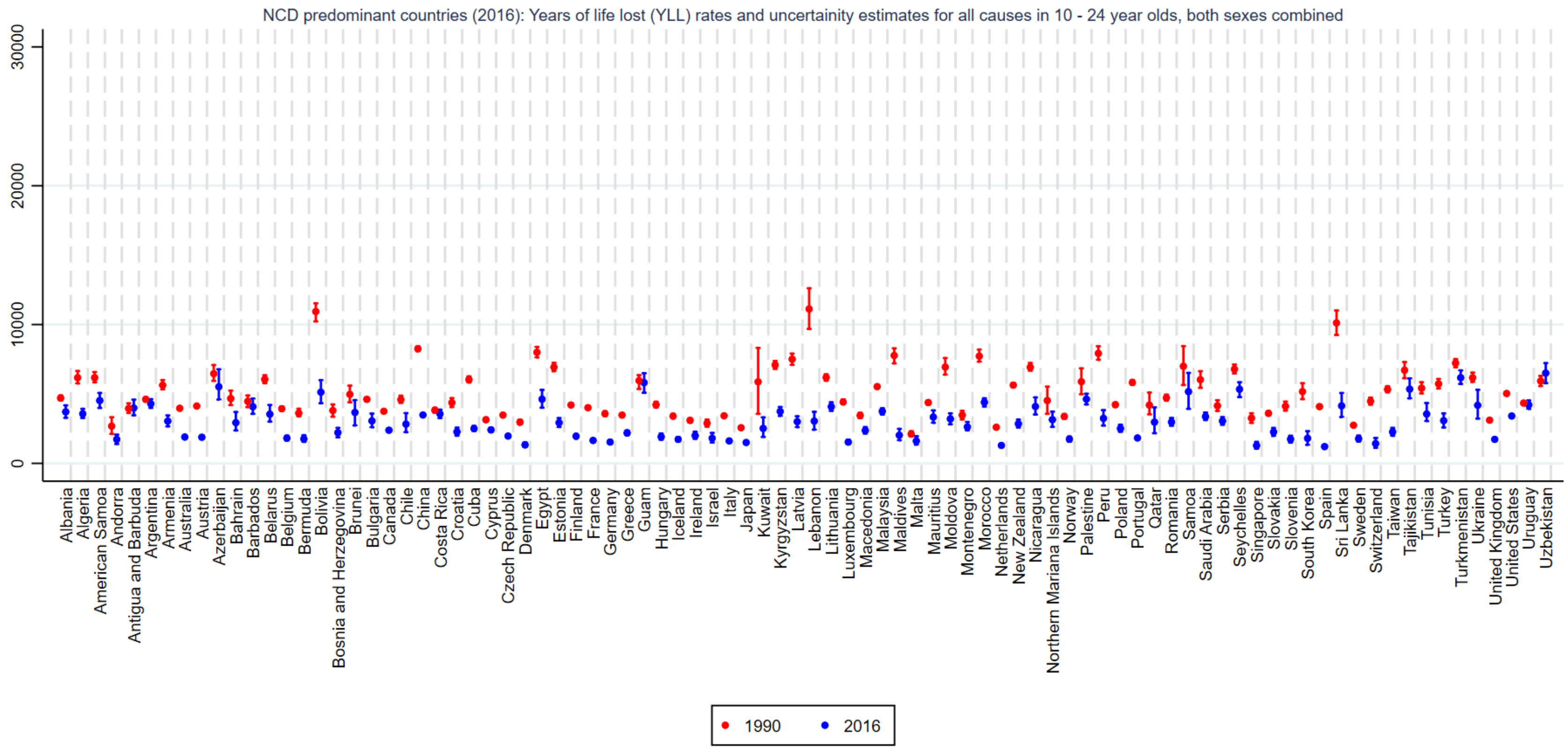

NCD predominant countries (2016): Years of life lost (YLL) rates and uncertainity estimates for all causes in 10 - 24 year olds, both sexes combined
0
10000
20000
30000
Albania
Algeria
American Samoa
Andorra
Antigua and Barbuda
Argentina
Armenia
Australia
Austria
Azerbaijan
Bahrain
Barbados
Belarus
Belgium
Bermuda
Bolivia
Bosnia and Herzegovina
Brunei
Bulgaria
Canada
Chile
China
Costa Rica
Croatia
Cuba
Cyprus
Czech Republic
Denmark
Egypt
Estonia
Finland
France
Germany
Greece
Guam
Hungary
Iceland
Ireland
Israel
Italy
Japan
Kuwait
Kyrgyzstan
Latvia
Lebanon
Lithuania
Luxembourg
Macedonia
Malaysia
Maldives
Malta
Mauritius
Moldova
Montenegro
Morocco
Netherlands
New Zealand
Nicaragua
Northern Mariana Islands
Norway
Palestine
Peru
Poland
Portugal
Qatar
Romania
Samoa
Saudi Arabia
Serbia
Seychelles
Singapore
Slovakia
Slovenia
South Korea
Spain
Sri Lanka
Sweden
Switzerland
Taiwan
Tajikistan
Tunisia
Turkey
Turkmenistan
Ukraine
United Kingdom
United States
Uruguay
Uzbekistan
1990
2016

Appendix 2.7 Years Lived with Disability (YLD) for all causes in 10 - 24 year olds

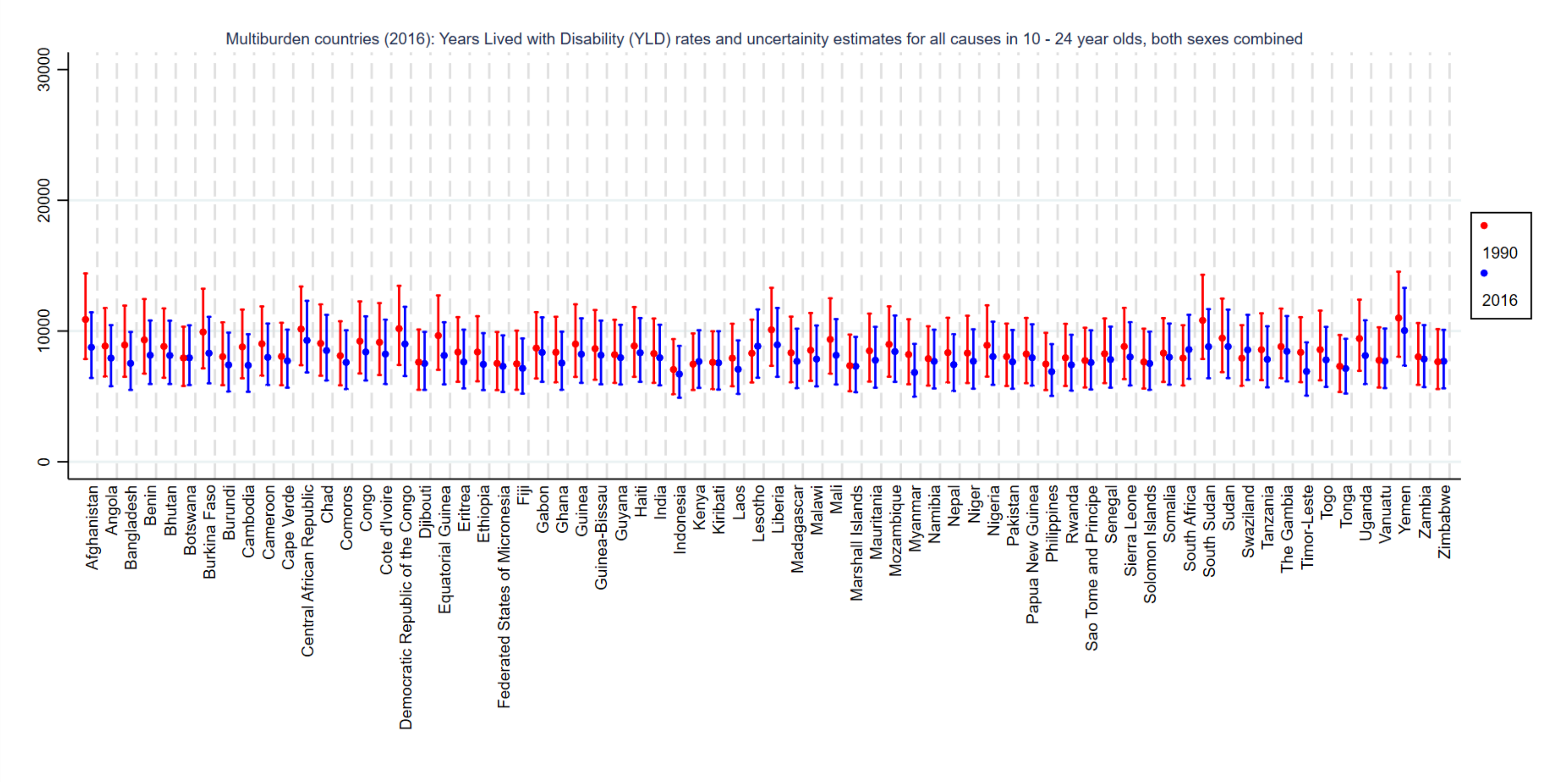

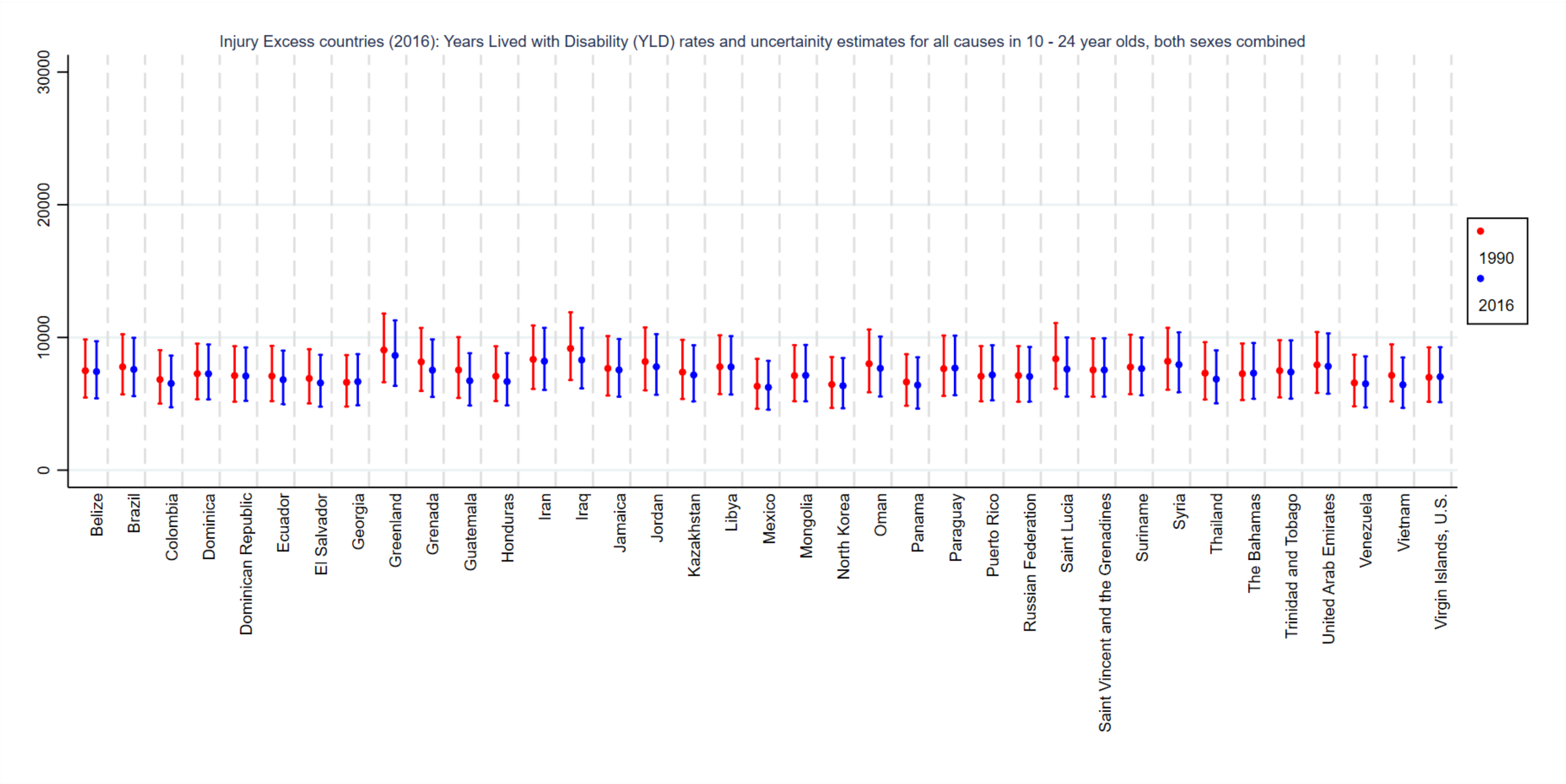

Injury Excess countries (2016): Years Lived with Disability (YLD) rates and uncertainity estimates for all causes in 10 - 24 year olds, both sexes combined
0
10000
20000
30000
1990
2016
Belize
Brazil
Colombia
Dominica
Dominican Republic
Ecuador
El Salvador
Georgia
Greenland
Grenada
Guatemala
Honduras
Iran
Iraq
Jamaica
Jordan
Kazakhstan
Libya
Mexico
Mongolia
North Korea
Oman
Panama
Paraguay
Puerto Rico
Russian Federation
Saint Lucia
Saint Vincent and the Grenadines
Suriname
Syria
Thailand
The Bahamas
Trinidad and Tobago
United Arab Emirates
Venezuela
Vietnam
Virgin Islands, U.S.

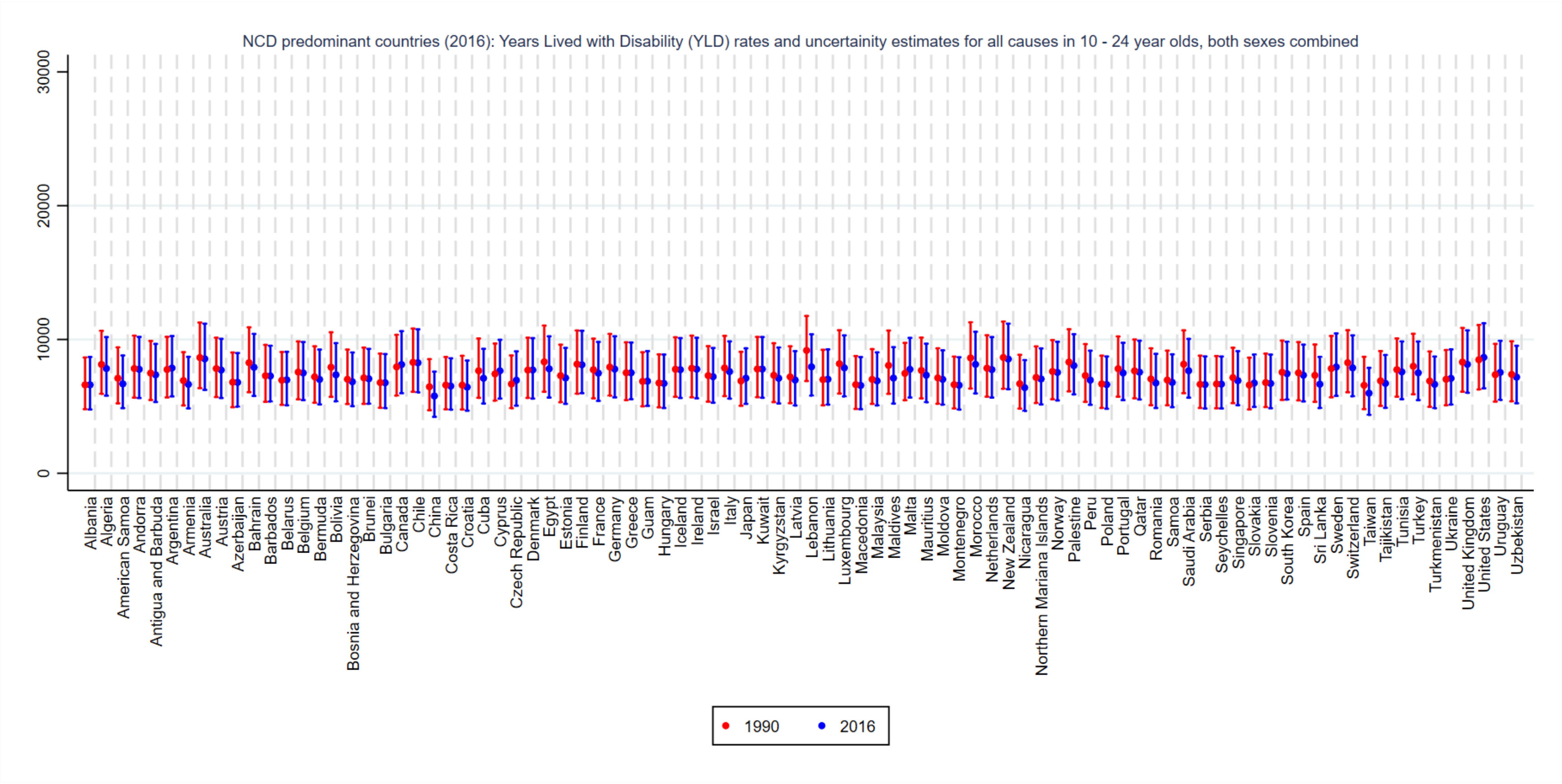
NCD predominant countries (2016): Years Lived with Disability (YLD) rates and uncertainity estimates for all causes in 10 - 24 year olds, both sexes combined
0
10000
20000
30000
Albania
Algeria
American Samoa
Andorra
Antigua and Barbuda
Argentina
Armenia
Australia
Austria
Azerbaijan
Bahrain
Barbados
Belarus
Belgium
Bermuda
Bolivia
Bosnia and Herzegovina
Brunei
Bulgaria
Canada
Chile
China
Costa Rica
Croatia
Cuba
Cyprus
Czech Republic
Denmark
Egypt
Estonia
Finland
France
Germany
Greece
Guam
Hungary
Iceland
Ireland
Israel
Italy
Japan
Kuwait
Kyrgyzstan
Latvia
Lebanon
Lithuania
Luxembourg
Macedonia
Malaysia
Maldives
Malta
Mauritius
Moldova
Montenegro
Morocco
Netherlands
New Zealand
Nicaragua
Northern Mariana Islands
Norway
Palestine
Peru
Poland
Portugal
Qatar
Romania
Samoa
Saudi Arabia
Serbia
Seychelles
Singapore
Slovakia
Slovenia
South Korea
Spain
Sri Lanka
Sweden
Switzerland
Taiwan
Tajikistan
Tunisia
Turkey
Turkmenistan
Ukraine
United Kingdom
United States
Uruguay
Uzbekistan
1990
2016

Appendix 2.8 Prevalence of daily use of any smoked tobacco product in 10 - 24 year olds

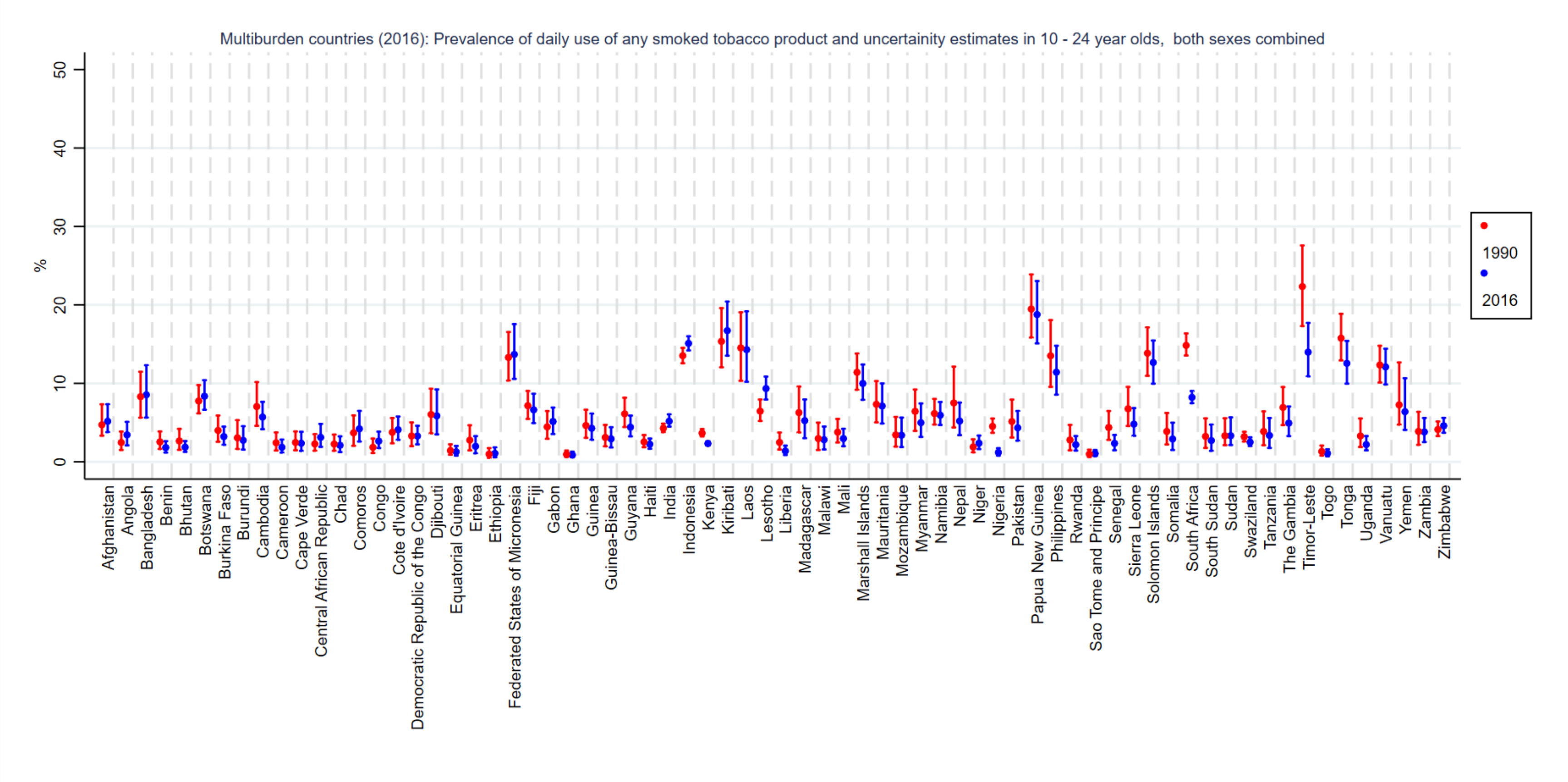

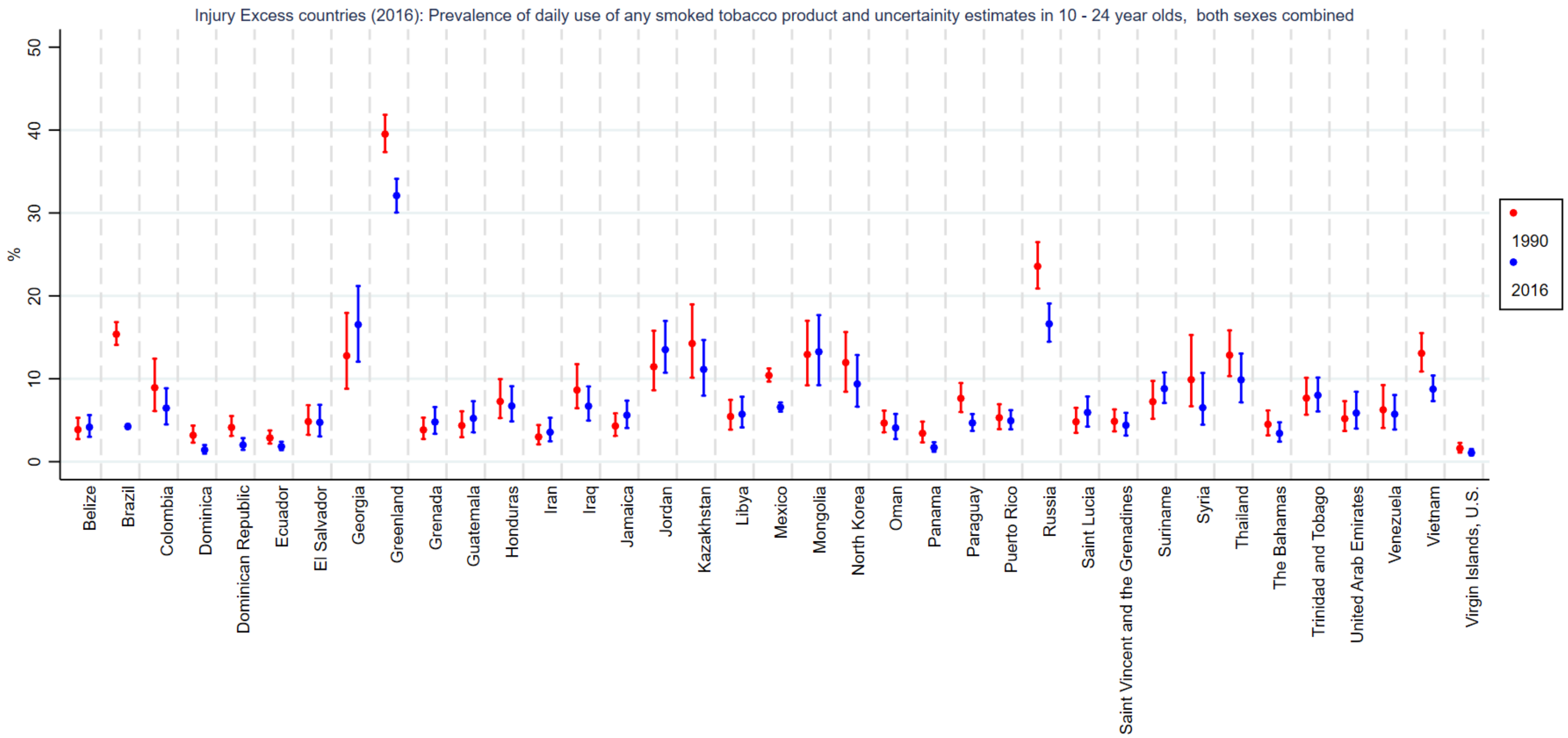
Injury Excess countries (2016): Prevalence of daily use of any smoked tobacco product and uncertainity estimates in 10 - 24 year olds, both sexes combined
%
0
10
20
30
40
50
Belize
Brazil
Colombia
Dominica
Dominican Republic
Ecuador
El Salvador
Georgia
Greenland
Grenada
Guatemala
Honduras
Iran
Iraq
Jamaica
Jordan
Kazakhstan
Libya
Mexico
Mongolia
North Korea
Oman
Panama
Paraguay
Puerto Rico
Russia
Saint Lucia
Saint Vincent and the Grenadines
Suriname
Syria
Thailand
The Bahamas
Trinidad and Tobago
United Arab Emirates
Venezuela
Vietnam
Virgin Islands, U.S.
1990
2016

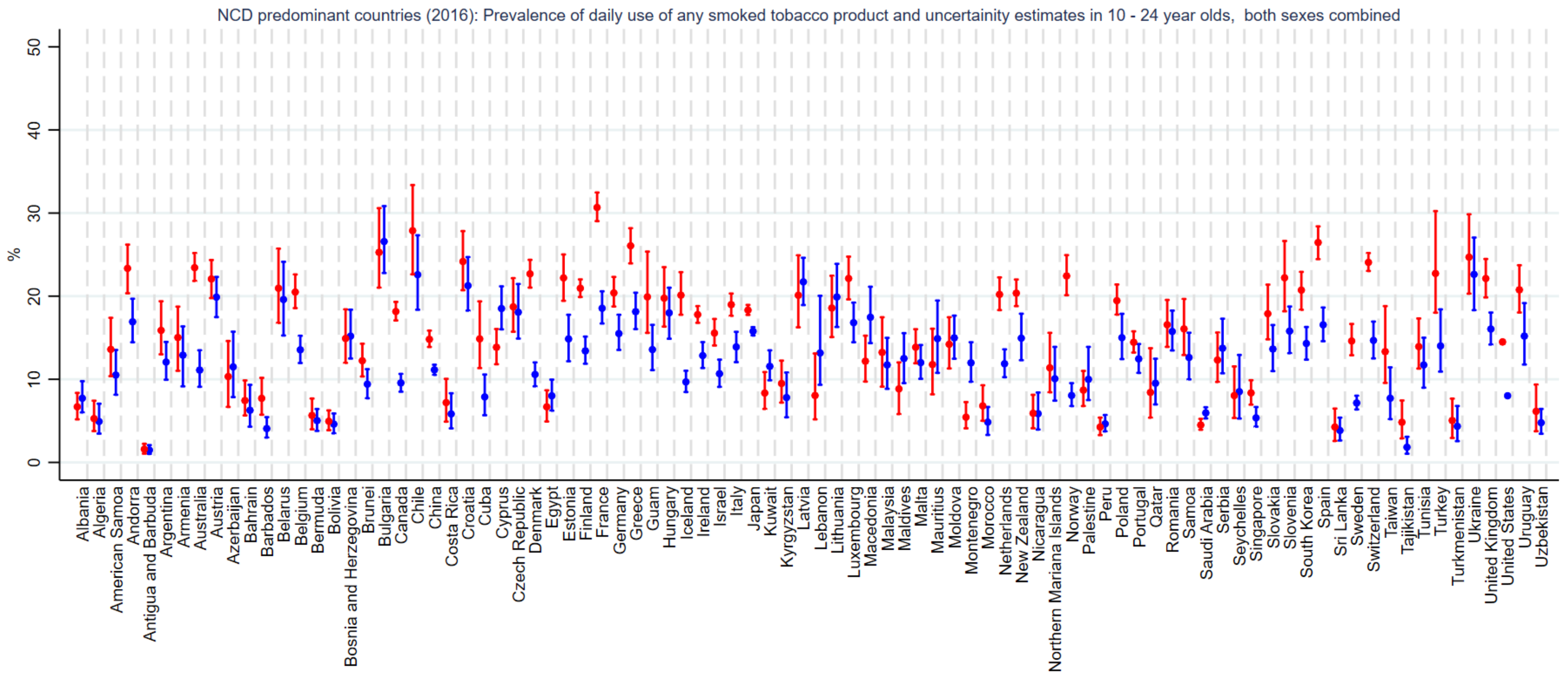
NCD predominant countries (2016): Prevalence of daily use of any smoked tobacco product and uncertainity estimates in 10 - 24 year olds, both sexes combined
%
0
10
20
30
40
50
Albania
Algeria
American Samoa
Andorra
Antigua and Barbuda
Argentina
Armenia
Australia
Austria
Azerbaijan
Bahrain
Barbados
Belarus
Belgium
Bermuda
Bolivia
Bosnia and Herzegovina
Brunei
Bulgaria
Canada
Chile
China
Costa Rica
Croatia
Cuba
Cyprus
Czech Republic
Denmark
Egypt
Estonia
Finland
France
Germany
Greece
Guam
Hungary
Iceland
Ireland
Israel
Italy
Japan
Kuwait
Kyrgyzstan
Latvia
Lebanon
Lithuania
Luxembourg
Macedonia
Malaysia
Maldives
Malta
Mauritius
Moldova
Montenegro
Morocco
Netherlands
New Zealand
Nicaragua
Northern Mariana Islands
Norway
Palestine
Peru
Poland
Portugal
Qatar
Romania
Samoa
Saudi Arabia
Serbia
Seychelles
Singapore
Slovakia
Slovenia
South Korea
Spain
Sri Lanka
Sweden
Switzerland
Taiwan
Tajikistan
Tunisia
Turkey
Turkmenistan
Ukraine
United Kingdom
United States
Uruguay
Uzbekistan
1990
2016

Appendix 2.9 Prevalence of episode of binge alcohol use (>48 grams of alcohol for females, >60g for males) in the past 12 months for 15 - 19 year olds

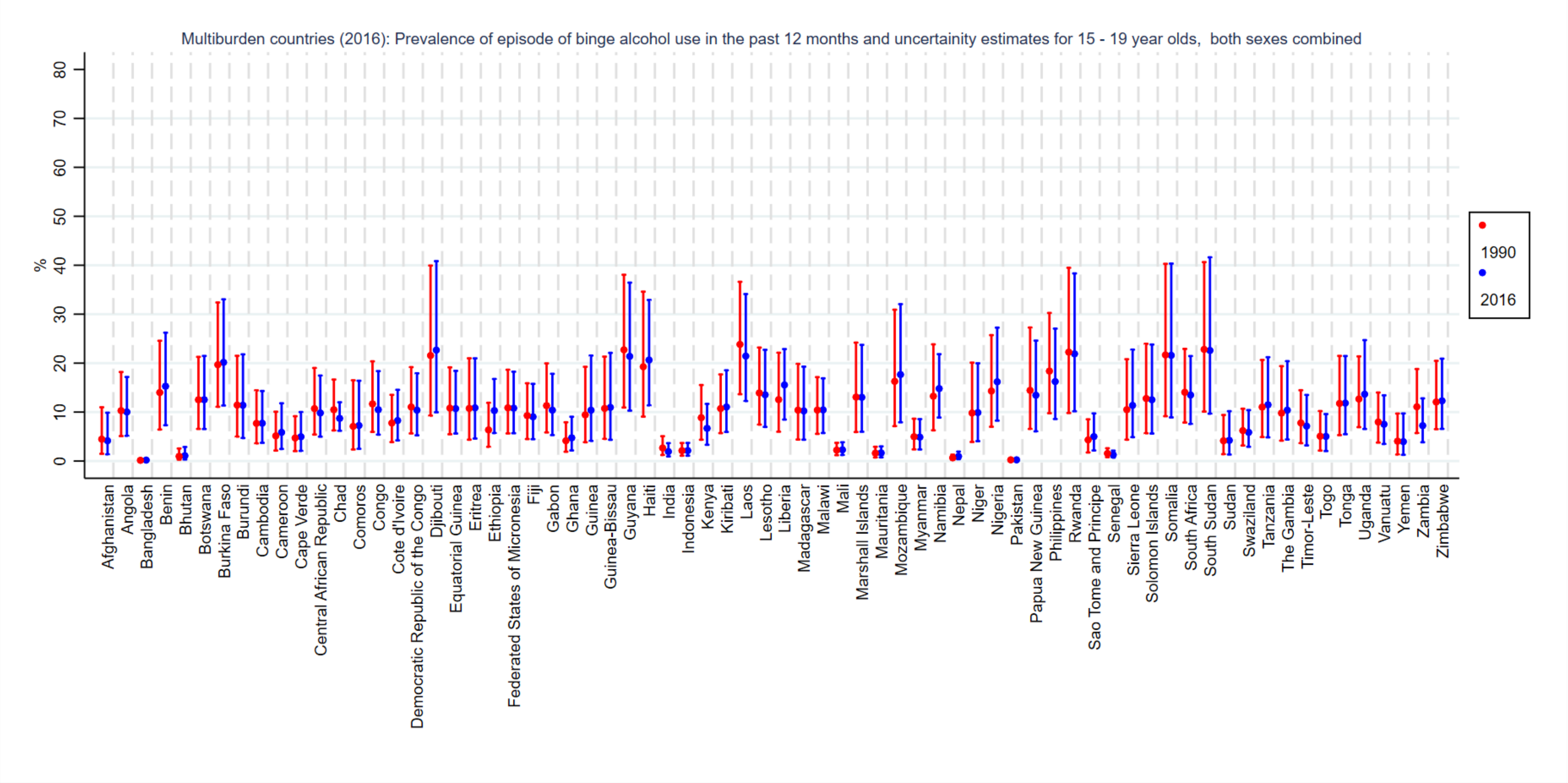

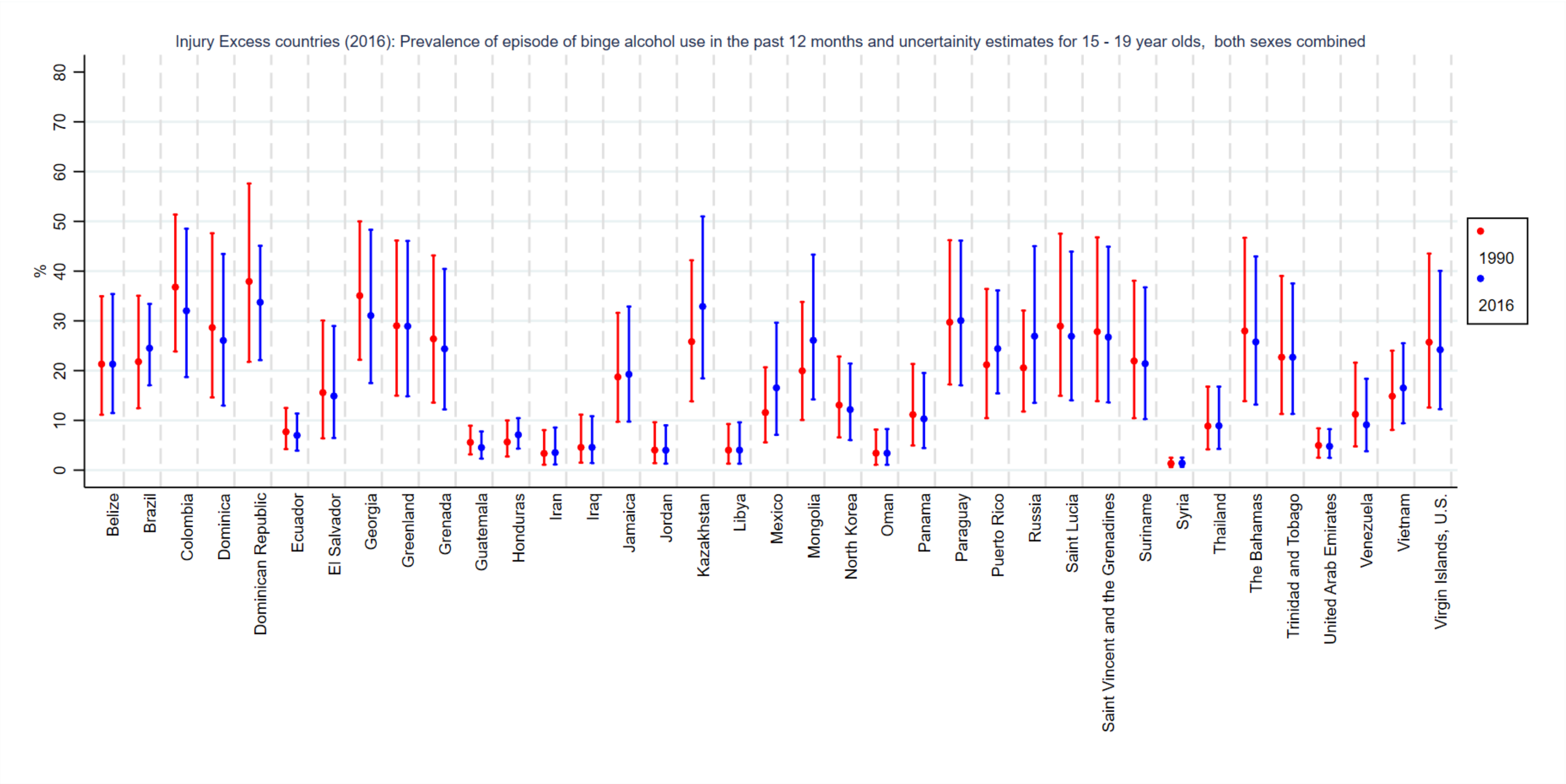
Injury Excess countries (2016): Prevalence of episode of binge alcohol use in the past 12 months and uncertainity estimates for 15 - 19 year olds, both sexes combined
%
0
10
20
30
40
50
60
70
80
1990
2016
Belize
Brazil
Colombia
Dominica
Dominican Republic
Ecuador
El Salvador
Georgia
Greenland
Grenada
Guatemala
Honduras
Iran
Iraq
Jamaica
Jordan
Kazakhstan
Libya
Mexico
Mongolia
North Korea
Oman
Panama
Paraguay
Puerto Rico
Russia
Saint Lucia
Saint Vincent and the Grenadines
Suriname
Syria
Thailand
The Bahamas
Trinidad and Tobago
United Arab Emirates
Venezuela
Vietnam
Virgin Islands, U.S.

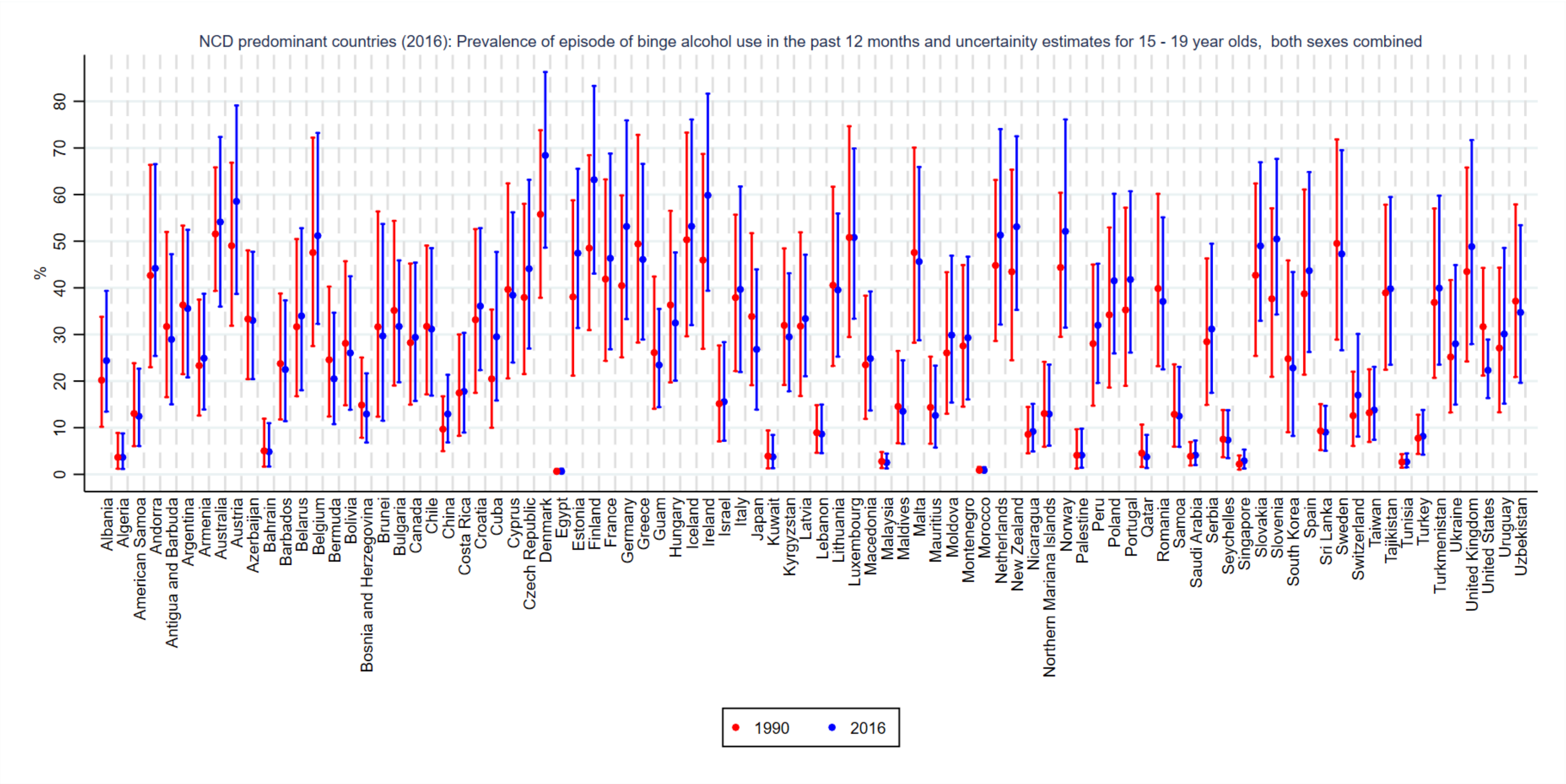

NCD predominant countries (2016): Prevalence of episode of binge alcohol use in the past 12 months and uncertainity estimates for 15 - 19 year olds, both sexes combined
%
0
10
20
30
40
50
60
70
80
Albania
Algeria
American Samoa
Andorra
Antigua and Barbuda
Argentina
Armenia
Australia
Austria
Azerbaijan
Bahrain
Barbados
Belarus
Belgium
Bermuda
Bolivia
Bosnia and Herzegovina
Brunei
Bulgaria
Canada
Chile
China
Costa Rica
Croatia
Cuba
Cyprus
Czech Republic
Denmark
Egypt
Estonia
Finland
France
Germany
Greece
Guam
Hungary
Iceland
Ireland
Israel
Italy
Japan
Kuwait
Kyrgyzstan
Latvia
Lebanon
Lithuania
Luxembourg
Macedonia
Malaysia
Maldives
Malta
Mauritius
Moldova
Montenegro
Morocco
Netherlands
New Zealand
Nicaragua
Northern Mariana Islands
Norway
Palestine
Peru
Poland
Portugal
Qatar
Romania
Samoa
Saudi Arabia
Serbia
Seychelles
Singapore
Slovakia
Slovenia
South Korea
Spain
Sri Lanka
Sweden
Switzerland
Taiwan
Tajikistan
Tunisia
Turkey
Turkmenistan
Ukraine
United Kingdom
United States
Uruguay
Uzbekistan
1990
2016

Appendix 2.10 Prevalence of overweight and obesity (IOTF thresholds, age and gender specific thresholds, equivalent to a BMI ≥25kg/m2 at age 18 years) in 10 - 24 year olds

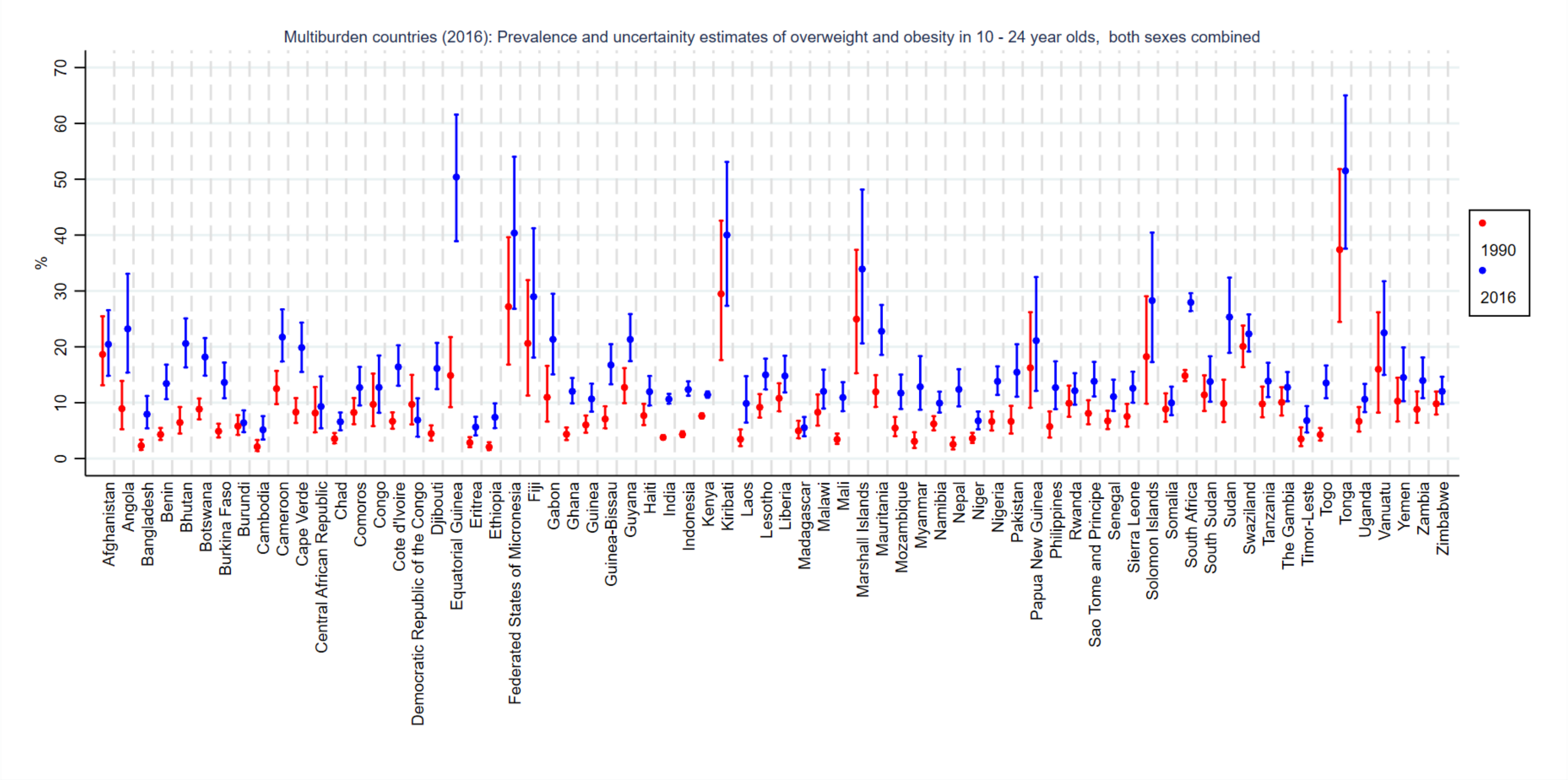

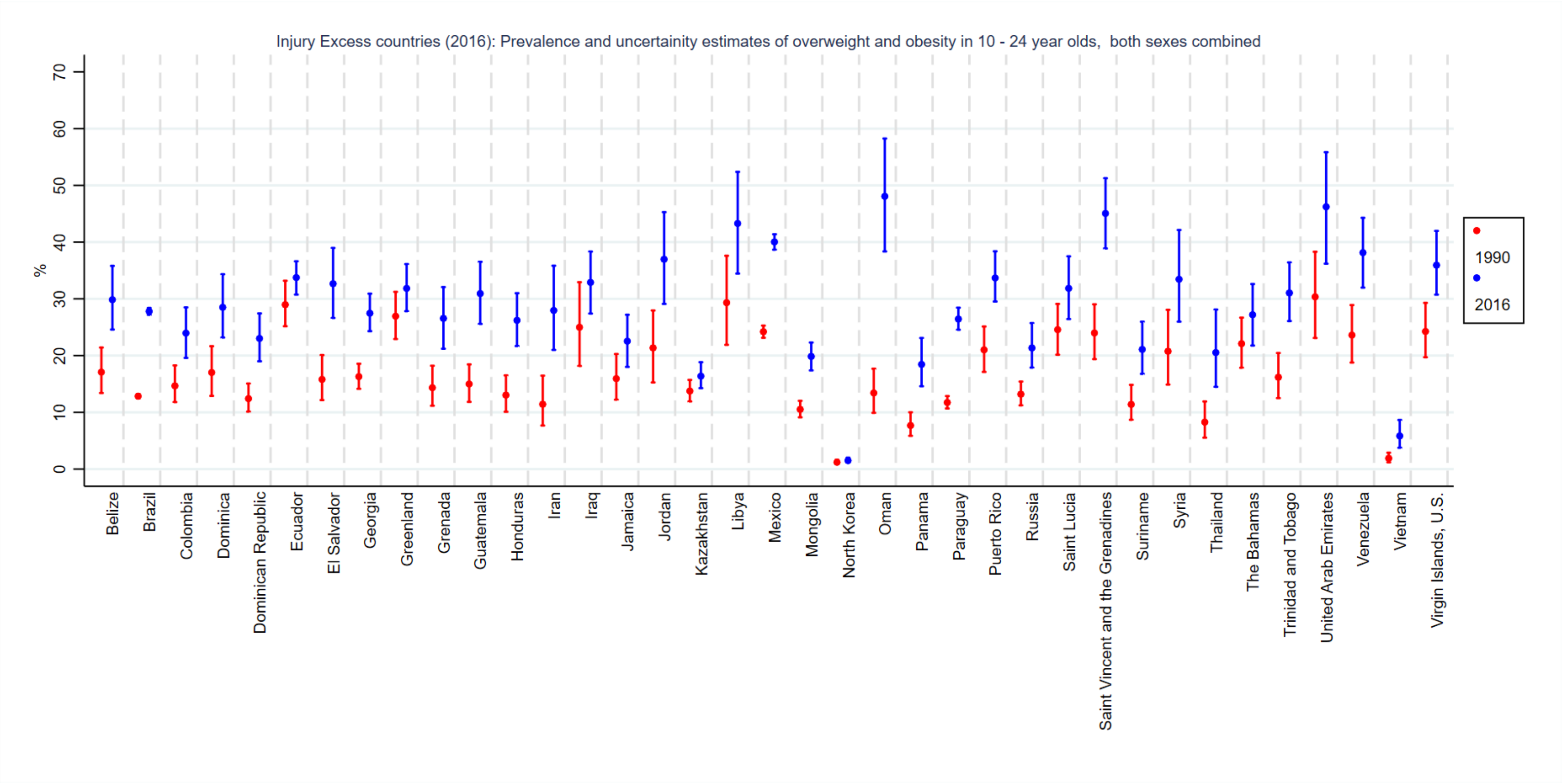
Injury Excess countries (2016): Prevalence and uncertainity estimates of overweight and obesity in 10 - 24 year olds, both sexes combined
%
0
10
20
30
40
50
60
70
Belize
Brazil
Colombia
Dominica
Dominican Republic
Ecuador
El Salvador
Georgia
Greenland
Grenada
Guatemala
Honduras
Iran
Iraq
Jamaica
Jordan
Kazakhstan
Libya
Mexico
Mongolia
North Korea
Oman
Panama
Paraguay
Puerto Rico
Russia
Saint Lucia
Saint Vincent and the Grenadines
Suriname
Syria
Thailand
The Bahamas
Trinidad and Tobago
United Arab Emirates
Venezuela
Vietnam
Virgin Islands, U.S.
1990
2016

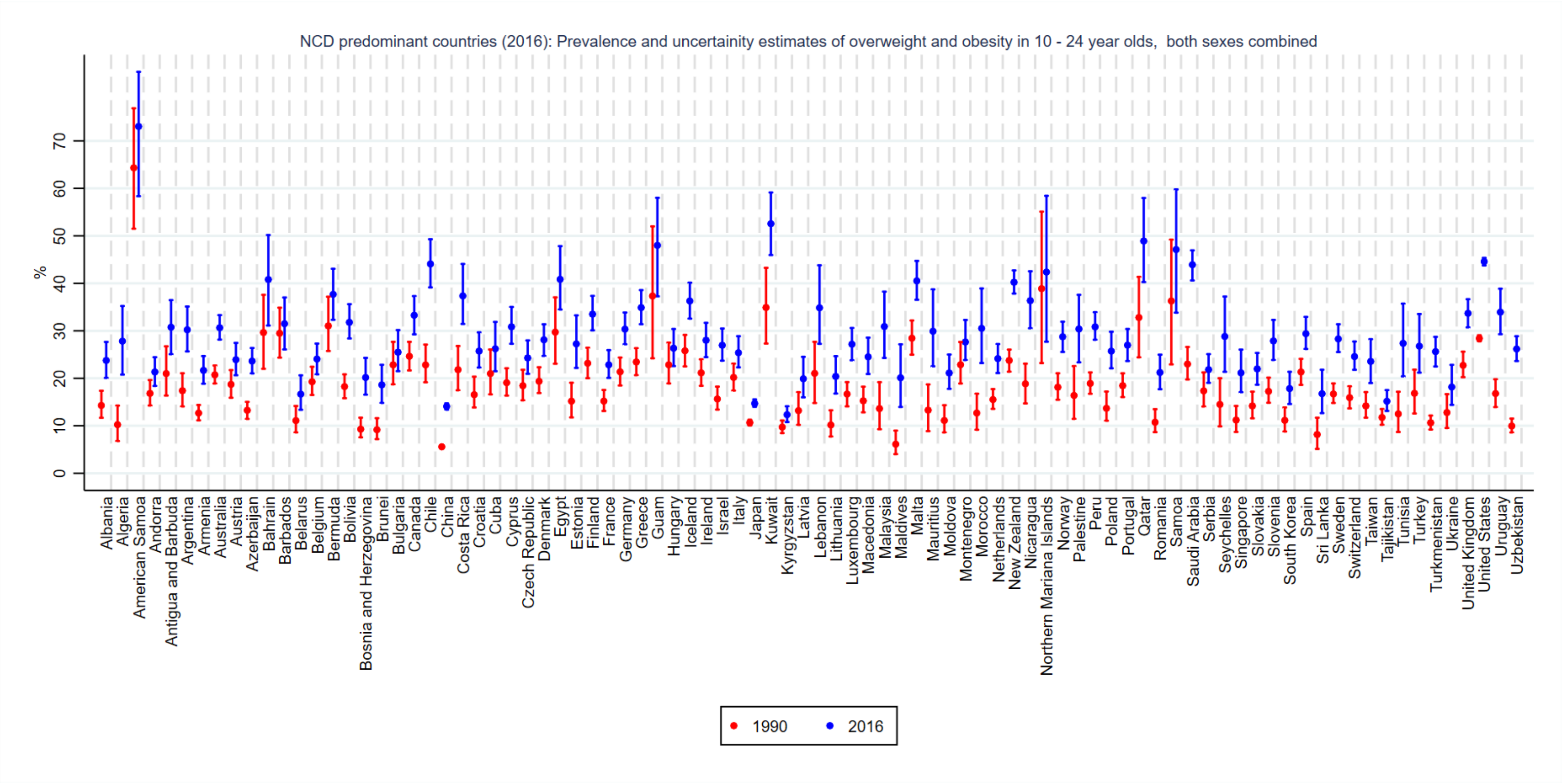

NCD predominant countries (2016): Prevalence and uncertainity estimates of overweight and obesity in 10 - 24 year olds, both sexes combined
%
0
10
20
30
40
50
60
70
Albania
Algeria
American Samoa
Andorra
Antigua and Barbuda
Argentina
Armenia
Australia
Austria
Azerbaijan
Bahrain
Barbados
Belarus
Belgium
Bermuda
Bolivia
Bosnia and Herzegovina
Brunei
Bulgaria
Canada
Chile
China
Costa Rica
Croatia
Cuba
Cyprus
Czech Republic
Denmark
Egypt
Estonia
Finland
France
Germany
Greece
Guam
Hungary
Iceland
Ireland
Israel
Italy
Japan
Kuwait
Kyrgyzstan
Latvia
Lebanon
Lithuania
Luxembourg
Macedonia
Malaysia
Maldives
Malta
Mauritius
Moldova
Montenegro
Morocco
Netherlands
New Zealand
Nicaragua
Northern Mariana Islands
Norway
Palestine
Peru
Poland
Portugal
Qatar
Romania
Samoa
Saudi Arabia
Serbia
Seychelles
Singapore
Slovakia
Slovenia
South Korea
Spain
Sri Lanka
Sweden
Switzerland
Taiwan
Tajikistan
Tunisia
Turkey
Turkmenistan
Ukraine
United Kingdom
United States
Uruguay
Uzbekistan
1990
2016

Appendix 2.11 Prevalence of anaemia (haemoglobin in g/L: 10 - 14 y <115; 15 - 24 y <130 males, <120 non-pregnant female, <110 pregnant) in 10 - 24 year olds

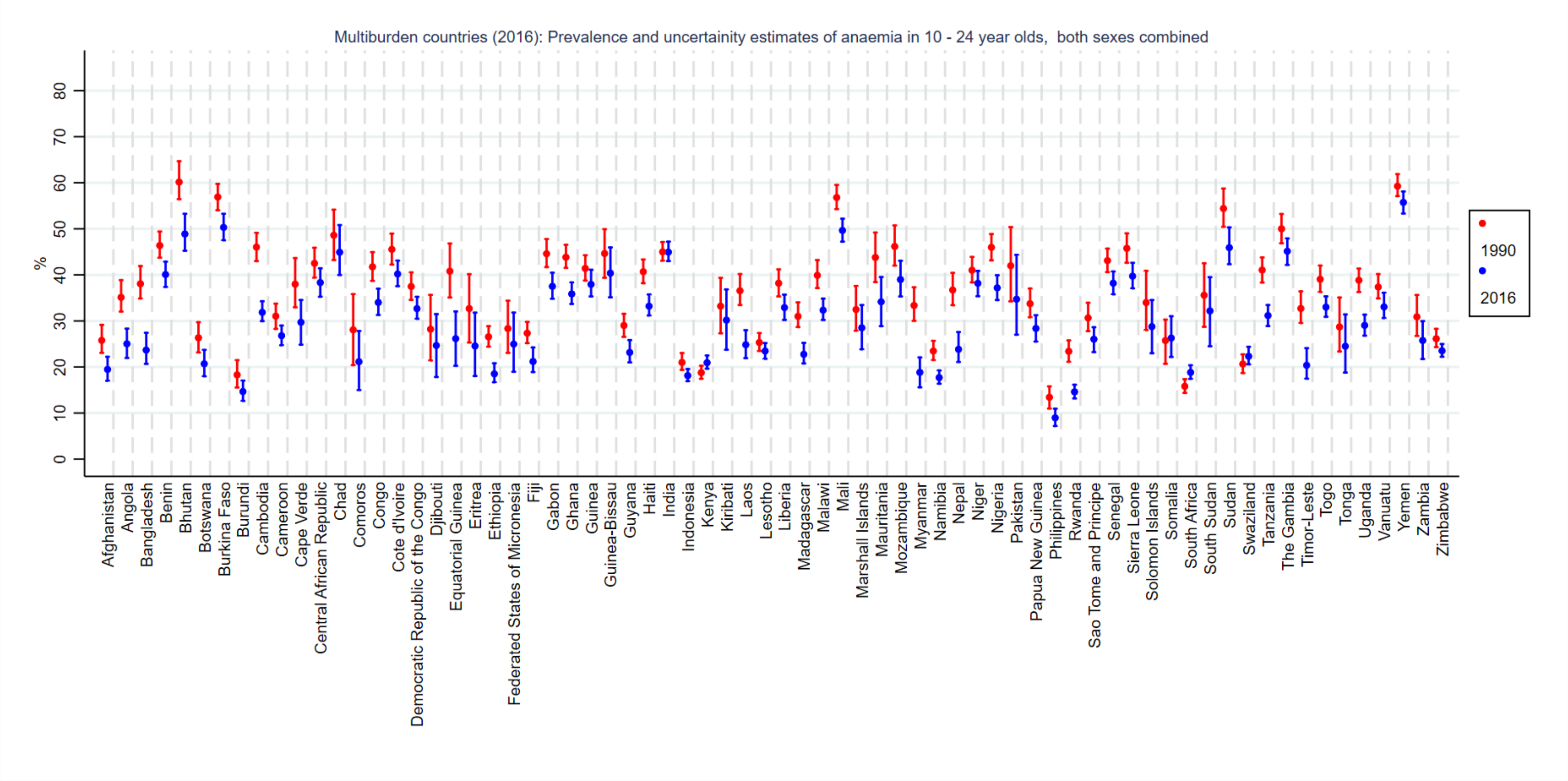

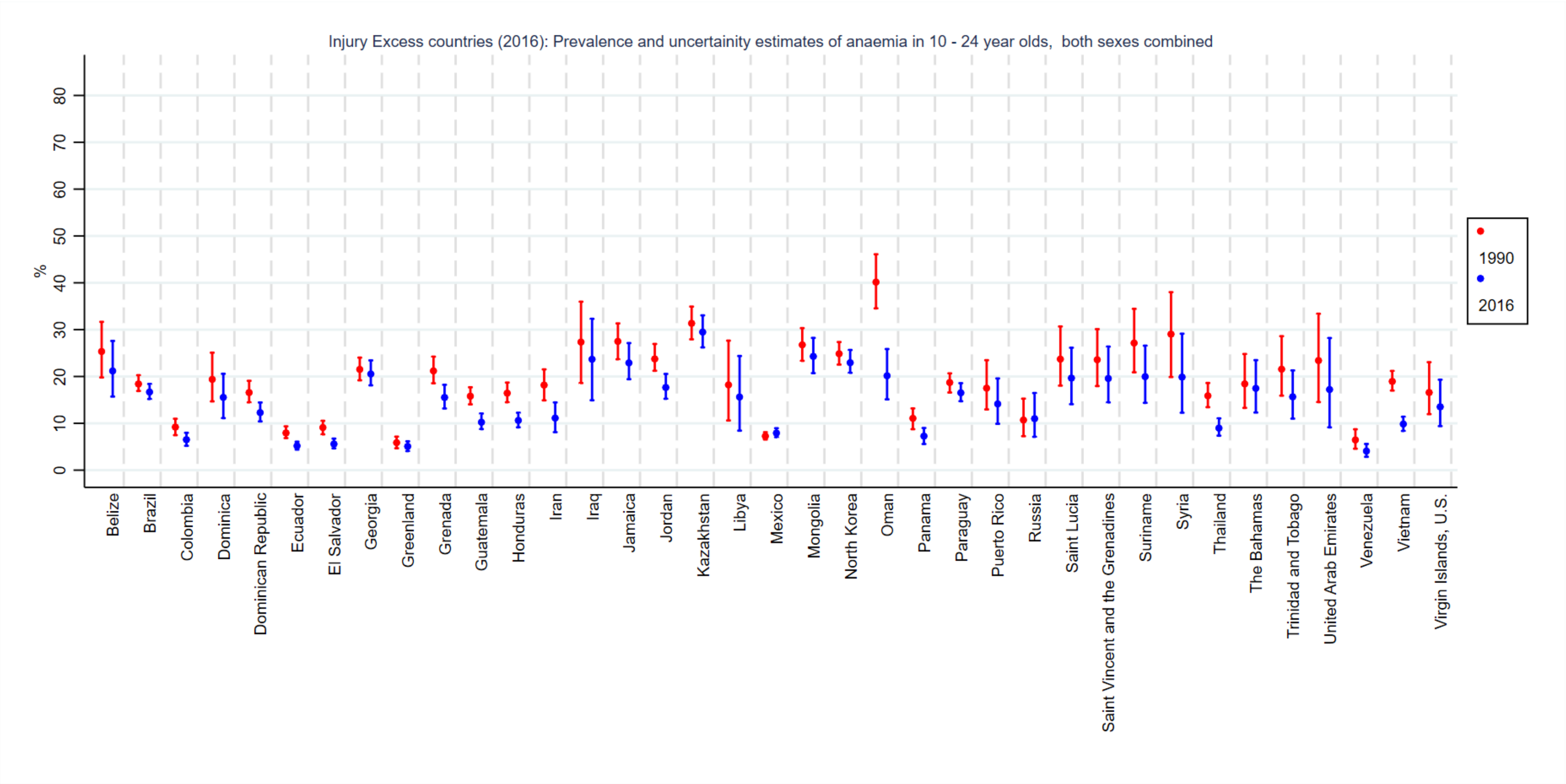
Injury Excess countries (2016): Prevalence and uncertainity estimates of anaemia in 10 - 24 year olds, both sexes combined
%
0
10
20
30
40
50
60
70
80
Belize
Brazil
Colombia
Dominica
Dominican Republic
Ecuador
El Salvador
Georgia
Greenland
Grenada
Guatemala
Honduras
Iran
Iraq
Jamaica
Jordan
Kazakhstan
Libya
Mexico
Mongolia
North Korea
Oman
Panama
Paraguay
Puerto Rico
Russia
Saint Lucia
Saint Vincent and the Grenadines
Suriname
Syria
Thailand
The Bahamas
Trinidad and Tobago
United Arab Emirates
Venezuela
Vietnam
Virgin Islands, U.S.
1990
2016

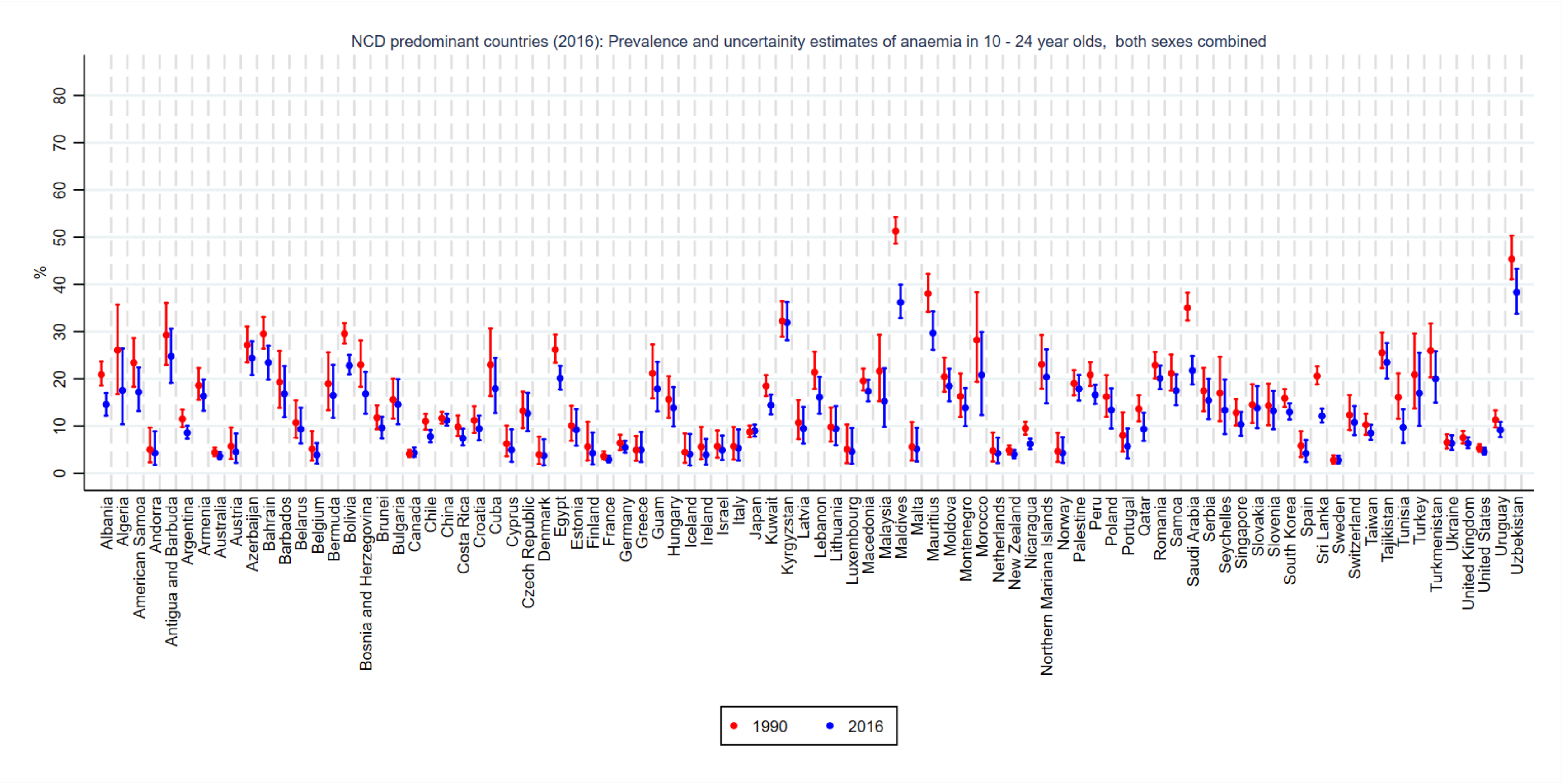
NCD predominant countries (2016): Prevalence and uncertainity estimates of anaemia in 10 - 24 year olds, both sexes combined
%
1990
2016

Appendix 2.12 Proportion of 15 - 24 year old females whose demand for contraceptive is satisfied with a modern method

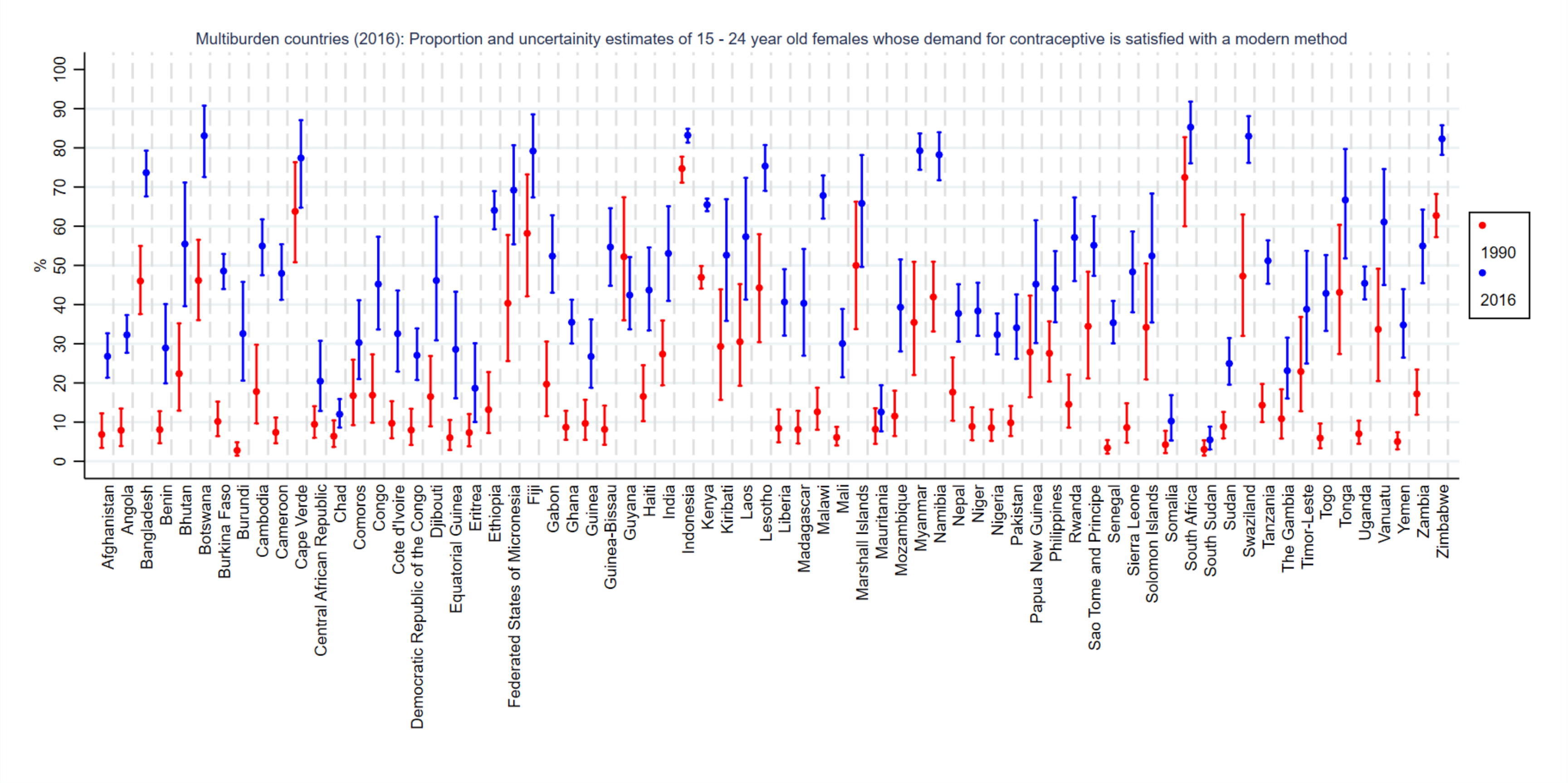

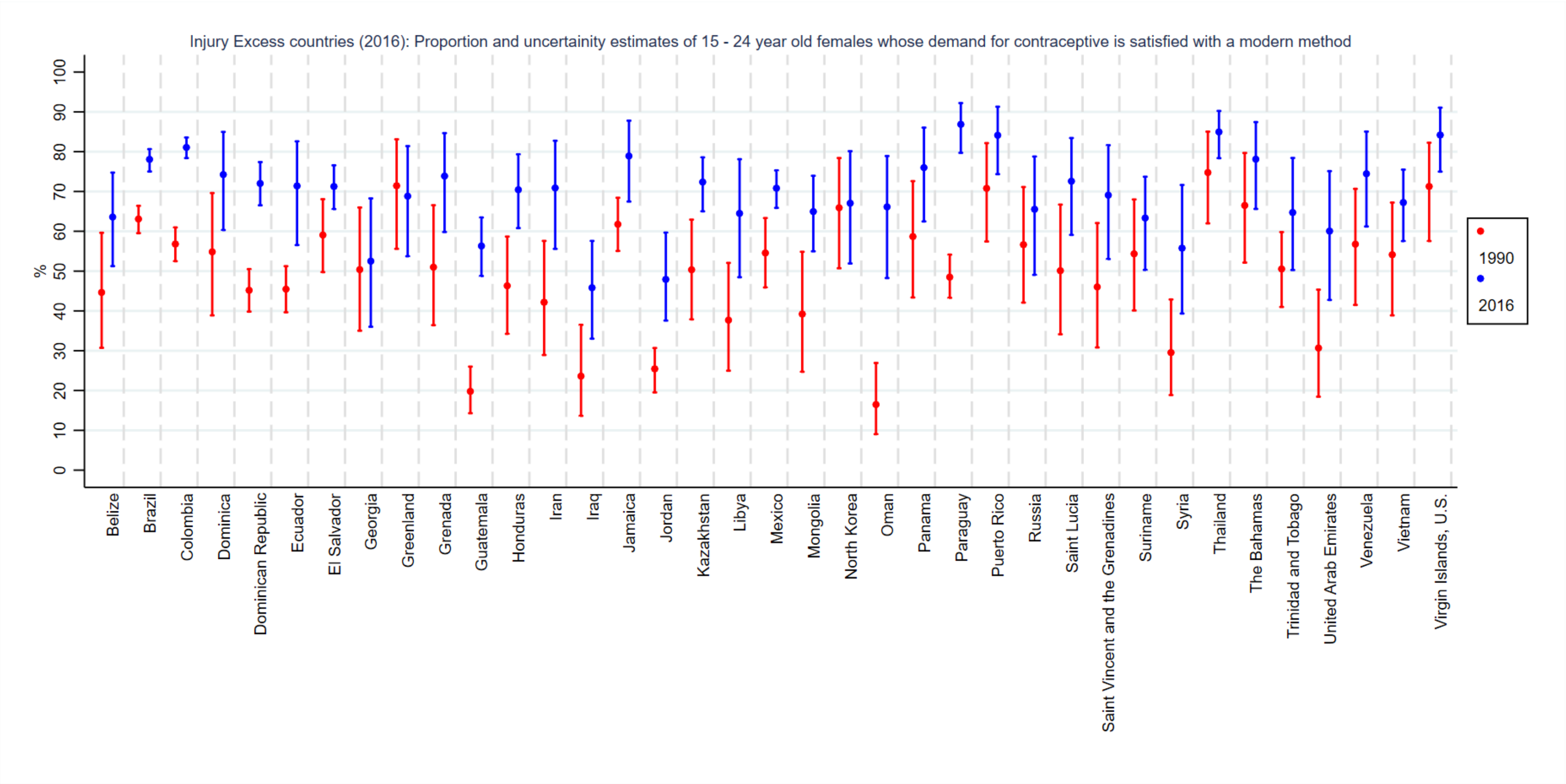
Injury Excess countries (2016): Proportion and uncertainity estimates of 15 - 24 year old females whose demand for contraceptive is satisfied with a modern method
%
0
10
20
30
40
50
60
70
80
90
100
Belize
Brazil
Colombia
Dominica
Dominican Republic
Ecuador
El Salvador
Georgia
Greenland
Grenada
Guatemala
Honduras
Iran
Iraq
Jamaica
Jordan
Kazakhstan
Libya
Mexico
Mongolia
North Korea
Oman
Panama
Paraguay
Puerto Rico
Russia
Saint Lucia
Saint Vincent and the Grenadines
Suriname
Syria
Thailand
The Bahamas
Trinidad and Tobago
United Arab Emirates
Venezuela
Vietnam
Virgin Islands, U.S.
1990
2016

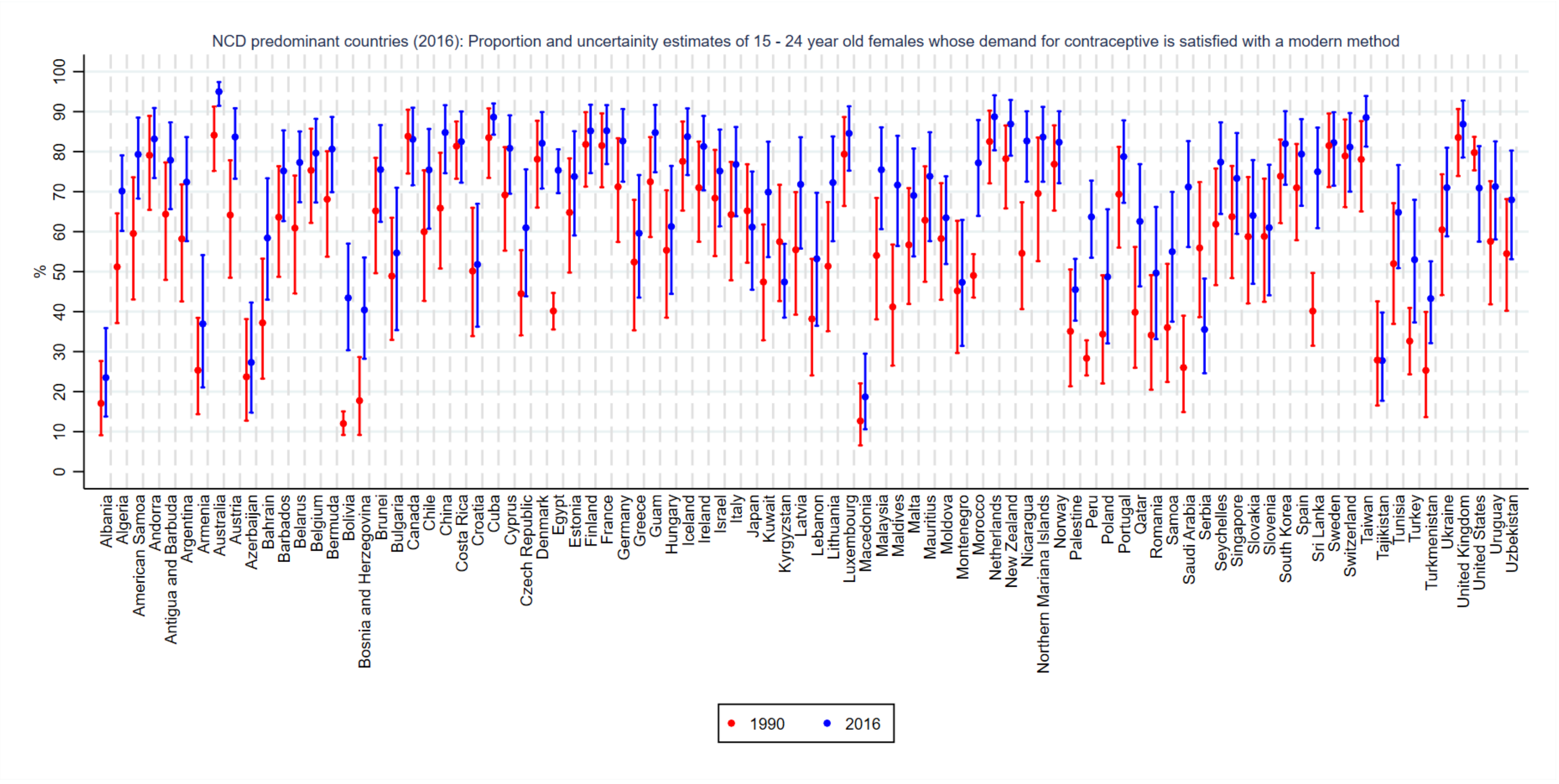
NCD predominant countries (2016): Proportion and uncertainity estimates of 15 - 24 year old females whose demand for contraceptive is satisfied with a modern method
%
0
10
20
30
40
50
60
70
80
90
100
Albania
Algeria
American Samoa
Andorra
Antigua and Barbuda
Argentina
Armenia
Australia
Austria
Azerbaijan
Bahrain
Barbados
Belarus
Belgium
Bermuda
Bolivia
Bosnia and Herzegovina
Brunei
Bulgaria
Canada
Chile
China
Costa Rica
Croatia
Cuba
Cyprus
Czech Republic
Denmark
Egypt
Estonia
Finland
France
Germany
Greece
Guam
Hungary
Iceland
Ireland
Israel
Italy
Japan
Kuwait
Kyrgyzstan
Latvia
Lebanon
Lithuania
Luxembourg
Macedonia
Malaysia
Maldives
Malta
Mauritius
Moldova
Montenegro
Morocco
Netherlands
New Zealand
Nicaragua
Northern Mariana Islands
Norway
Palestine
Peru
Poland
Portugal
Qatar
Romania
Samoa
Saudi Arabia
Serbia
Seychelles
Singapore
Slovakia
Slovenia
South Korea
Spain
Sri Lanka
Sweden
Switzerland
Taiwan
Tajikistan
Tunisia
Turkey
Turkmenistan
Ukraine
United Kingdom
United States
Uruguay
Uzbekistan
1990
2016

Appendix 4 – Population counts for each country and population scenarios

Table 4a. This table reports the population, SDI score and country grouping for each of the 195 countries in 1990 and 2016, as well as the World Bank (WB) Income group in 2016

	1990			2016				
Country	Population	SDI	Disease category	Population	SDI	Disease category	2016 WB income group	Population change (%)
Afghanistan	3,861,415	0.16	Multiburden	11,735,080	0.28	Multiburden	Low	203.9
Albania	956,107	0.58	Excess injury	701,146	0.72	NCD	Upper middle	-26.7
Algeria	8,739,186	0.46	Excess injury	9,431,689	0.68	NCD	Upper middle	7.9
American Samoa	14,611	0.62	Excess injury	20,029	0.74	NCD	Upper middle	37.1
Andorra	10,784	0.87	NCD	13,029	0.91	NCD	High	20.8
Angola	3,499,758	0.25	Multiburden	8,434,139	0.43	Multiburden	Upper middle	141.0
Antigua and Barbuda	17,232	0.68	NCD	23,297	0.78	NCD	High	35.2
Argentina	8,620,512	0.64	Excess injury	10,512,025	0.76	NCD	Upper middle	21.9
Armenia	879,484	0.64	Excess injury	571,828	0.75	NCD	Lower middle	-35.0
Australia	3,995,612	0.82	NCD	4,611,449	0.89	NCD	High	15.4
Austria	1,594,166	0.83	Excess injury	1,421,089	0.89	NCD	High	-10.9
Azerbaijan	2,091,201	0.67	Excess injury	2,116,573	0.77	NCD	Upper middle	1.2
Bahrain	124,628	0.59	NCD	301,718	0.74	NCD	High	142.1
Bangladesh	35,226,440	0.31	Multiburden	47,801,000	0.51	Multiburden	Lower middle	35.7
Barbados	71,412	0.73	NCD	55,566	0.80	NCD	High	-22.2
Belarus	2,145,329	0.74	Excess injury	1,470,221	0.83	NCD	Upper middle	-31.5
Belgium	2,002,439	0.84	Excess injury	1,954,487	0.90	NCD	High	-2.4
Belize	62,653	0.45	Excess injury	114,638	0.62	Excess injury	Upper middle	83.0
Benin	1,574,976	0.22	Multiburden	3,622,750	0.35	Multiburden	Low	130.0
Bermuda	16,714	0.73	NCD	18,088	0.82	NCD	High	8.2
Bhutan	165,628	0.30	Multiburden	220,576	0.59	Multiburden	Lower middle	33.2
Bolivia	2,169,246	0.42	Multiburden	3,230,908	0.61	NCD	Lower middle	48.9
Bosnia and Herzegovina	1,135,286	0.54	NCD	597,764	0.73	NCD	Upper middle	-47.3
Botswana	455,429	0.51	Multiburden	662,734	0.70	Multiburden	Upper middle	45.5
Brazil	46,287,744	0.54	Excess injury	51,353,976	0.71	Excess injury	Upper middle	10.9
Brunei	73,957	0.74	Excess injury	103,499	0.87	NCD	High	39.9
Bulgaria	1,879,226	0.76	Excess injury	1,031,074	0.85	NCD	Upper middle	-45.1
Burkina Faso	2,853,236	0.13	Multiburden	6,148,457	0.27	Multiburden	Low	115.5
Burundi	1,639,849	0.19	Multiburden	3,529,890	0.28	Multiburden	Low	115.3
Cambodia	2,509,609	0.30	Multiburden	4,703,125	0.50	Multiburden	Lower middle	87.4
Cameroon	3,727,117	0.34	Multiburden	7,799,218	0.45	Multiburden	Lower middle	109.3
Canada	5,940,649	0.85	Excess injury	6,500,305	0.91	NCD	High	9.4
Cape Verde	109,406	0.32	Multiburden	159,176	0.57	Multiburden	Lower middle	45.5
Central African Republic	894,939	0.25	Multiburden	1,612,402	0.32	Multiburden	Low	80.2
Chad	1,822,073	0.18	Multiburden	4,865,529	0.30	Multiburden	Low	167.0
Chile	3,704,563	0.64	NCD	3,957,753	0.80	NCD	High	6.8
China	351,669,472	0.51	Excess injury	252,732,944	0.73	NCD	Upper middle	-28.1
Colombia	10,956,756	0.55	Excess injury	12,269,012	0.71	Excess injury	Upper middle	12.0

Comoros	131,087	0.27	Multiburden	253,566	0.45	Multiburden	Low	93.4
Congo	776,579	0.38	Multiburden	1,452,999	0.53	Multiburden	Lower middle	87.1
Costa Rica	887,837	0.57	NCD	1,161,784	0.73	NCD	Upper middle	30.9
Cote d'Ivoire	3,720,824	0.28	Multiburden	7,592,306	0.41	Multiburden	Lower middle	104.0
Croatia	985,074	0.79	Excess injury	675,294	0.85	NCD	High	-31.4
Cuba	3,018,786	0.68	Excess injury	2,041,335	0.78	NCD	Upper middle	-32.4
Cyprus	171,965	0.75	Excess injury	183,498	0.87	NCD	High	6.7
Czech Republic	2,393,332	0.81	Excess injury	1,518,625	0.88	NCD	High	-36.5
DRC*	10,753,135	0.28	Multiburden	25,802,172	0.31	Multiburden	Low	140.0
Denmark	1,080,125	0.88	NCD	1,078,151	0.93	NCD	High	-0.2
Djibouti	191,808	0.20	Multiburden	270,004	0.42	Multiburden	Lower middle	40.8
Dominica	20,251	0.60	NCD	18,913	0.73	Excess injury	Upper middle	-6.6
Dominican Republic	2,334,103	0.53	Excess injury	2,959,746	0.70	Excess injury	Upper middle	26.8
Ecuador	3,279,478	0.53	Multiburden	4,467,885	0.67	Excess injury	Upper middle	36.2
Egypt	16,432,880	0.46	Multiburden	24,538,546	0.65	NCD	Lower middle	49.3
El Salvador	1,685,252	0.44	Excess injury	1,808,529	0.64	Excess injury	Lower middle	7.3
Equatorial Guinea	100,733	0.29	Multiburden	260,584	0.67	Multiburden	Upper middle	158.7
Eritrea	1,024,661	0.19	Multiburden	1,697,888	0.39	Multiburden	Low	65.7
Estonia	327,250	0.80	Excess injury	194,203	0.89	NCD	High	-40.7
Ethiopia	14,745,905	0.15	Multiburden	35,234,792	0.33	Multiburden	Low	138.9
FSM[!]	32,431	0.43	Multiburden	36,914	0.61	Multiburden	Lower middle	13.8
Fiji	223,679	0.60	Multiburden	233,620	0.74	Multiburden	Upper middle	4.4
Finland	976,178	0.84	NCD	934,547	0.91	NCD	High	-4.3
France	12,287,715	0.80	Excess injury	11,524,168	0.87	NCD	High	-6.2
Gabon	277,197	0.46	Multiburden	536,084	0.62	Multiburden	Upper middle	93.4
Georgia	1,246,700	0.72	Excess injury	706,166	0.75	Excess injury	Upper middle	-43.4
Germany	14,958,426	0.83	NCD	12,282,011	0.89	NCD	High	-17.9
Ghana	4,790,710	0.36	Multiburden	8,556,693	0.50	Multiburden	Lower middle	78.6
Greece	2,279,103	0.77	Excess injury	1,631,056	0.85	NCD	High	-28.4
Greenland	12,409	0.75	Excess injury	10,924	0.82	Excess injury	High	-12.0
Grenada	27,345	0.50	Multiburden	28,265	0.70	Excess injury	Upper middle	3.4
Guam	37,187	0.76	NCD	43,902	0.82	NCD	High	18.1
Guatemala	2,976,287	0.36	Multiburden	5,379,367	0.56	Excess injury	Lower middle	80.7
Guinea	1,791,189	0.21	Multiburden	4,156,846	0.32	Multiburden	Low	132.1
Guinea-Bissau	313,110	0.21	Multiburden	593,421	0.33	Multiburden	Low	89.5
Guyana	243,274	0.52	Multiburden	256,360	0.65	Multiburden	Upper middle	5.4
Haiti	2,154,447	0.30	Multiburden	3,340,203	0.43	Multiburden	Low	55.0
Honduras	1,622,820	0.38	Multiburden	2,642,154	0.57	Excess injury	Lower middle	62.8
Hungary	2,312,052	0.75	Excess injury	1,606,054	0.85	NCD	High	-30.5
Iceland	63,074	0.85	NCD	68,574	0.92	NCD	High	8.7
India	264,006,208	0.36	Multiburden	370,100,480	0.58	Multiburden	Lower middle	40.2
Indonesia	59,361,032	0.50	Multiburden	67,794,184	0.68	Multiburden	Lower middle	14.2
Iran	17,709,884	0.51	Excess injury	17,566,804	0.77	Excess injury	Upper middle	-0.8
Iraq	6,060,515	0.39	Multiburden	11,661,292	0.47	Excess injury	Upper middle	92.4
Ireland	946,662	0.77	NCD	823,314	0.89	NCD	High	-13.0
Israel	1,247,064	0.74	NCD	1,897,656	0.80	NCD	High	52.2

Italy	12,520,734	0.80	NCD	8,767,832	0.87	NCD	High	-30.0
Jamaica	759,042	0.60	NCD	760,812	0.70	Excess injury	Upper middle	0.2
Japan	27,262,858	0.82	NCD	17,385,084	0.88	NCD	High	-36.2
Jordan	1,193,971	0.48	Excess injury	2,294,129	0.69	Excess injury	Upper middle	92.1
Kazakhstan	4,362,833	0.68	Excess injury	3,734,792	0.76	Excess injury	Upper middle	-14.4
Kenya	7,839,933	0.33	Multiburden	14,969,302	0.52	Multiburden	Lower middle	90.9
Kiribati	21,774	0.31	Multiburden	33,397	0.45	Multiburden	Lower middle	53.4
Kuwait	610,571	0.65	Excess injury	771,004	0.83	NCD	High	26.3
Kyrgyzstan	1,268,816	0.60	Multiburden	1,548,143	0.63	NCD	Lower middle	22.0
Laos	1,371,560	0.30	Multiburden	2,241,457	0.51	Multiburden	Lower middle	63.4
Latvia	544,022	0.78	Excess injury	286,021	0.85	NCD	High	-47.4
Lebanon	819,825	0.63	Excess injury	1,632,587	0.81	NCD	Upper middle	99.1
Lesotho	534,316	0.37	Multiburden	727,450	0.55	Multiburden	Lower middle	36.1
Liberia	664,418	0.22	Multiburden	1,477,320	0.33	Multiburden	Low	122.3
Libya	1,524,059	0.61	Excess injury	1,588,949	0.81	Excess injury	Upper middle	4.3
Lithuania	817,432	0.79	Excess injury	494,746	0.88	NCD	High	-39.5
Luxembourg	71,667	0.88	Excess injury	100,765	0.94	NCD	High	40.6
Macedonia	511,841	0.70	NCD	403,856	0.79	NCD	Upper middle	-21.1
Madagascar	3,779,694	0.25	Multiburden	8,187,105	0.37	Multiburden	Low	116.6
Malawi	3,016,248	0.20	Multiburden	5,921,904	0.35	Multiburden	Low	96.3
Malaysia	5,525,700	0.56	Excess injury	8,218,086	0.77	NCD	Upper middle	48.7
Maldives	69,146	0.43	Multiburden	98,633	0.69	NCD	Upper middle	42.6
Mali	2,728,958	0.14	Multiburden	5,877,705	0.27	Multiburden	Low	115.4
Malta	78,312	0.75	NCD	75,523	0.86	NCD	High	-3.6
Marshall Islands	14,816	0.47	Multiburden	22,726	0.58	Multiburden	Upper middle	53.4
Mauritania	660,969	0.32	Multiburden	1,308,509	0.48	Multiburden	Lower middle	98.0
Mauritius	330,099	0.61	NCD	294,298	0.76	NCD	Upper middle	-10.8
Mexico	29,429,604	0.56	Excess injury	35,169,720	0.73	Excess injury	Upper middle	19.5
Moldova	1,008,754	0.65	Excess injury	739,980	0.70	NCD	Lower middle	-26.6
Mongolia	718,179	0.56	Multiburden	708,160	0.68	Excess injury	Lower middle	-1.4
Montenegro	153,182	0.75	NCD	125,265	0.82	NCD	Upper middle	-18.2
Morocco	8,200,806	0.38	Multiburden	8,862,646	0.62	NCD	Lower middle	8.1
Mozambique	4,410,417	0.14	Multiburden	9,547,762	0.31	Multiburden	Low	116.5
Myanmar	13,645,516	0.35	Multiburden	15,009,832	0.59	Multiburden	Lower middle	10.0
Namibia	474,797	0.43	Multiburden	796,112	0.62	Multiburden	Upper middle	67.7
Nepal	5,798,511	0.24	Multiburden	9,442,337	0.45	Multiburden	Low	62.8
Netherlands	3,244,878	0.85	NCD	3,141,794	0.92	NCD	High	-3.2
New Zealand	821,952	0.80	Excess injury	937,232	0.87	NCD	High	14.0
Nicaragua	1,374,691	0.43	Excess injury	1,791,930	0.59	NCD	Lower middle	30.4
Niger	2,372,583	0.14	Multiburden	6,457,155	0.20	Multiburden	Low	172.2
Nigeria	29,996,162	0.32	Multiburden	58,859,016	0.48	Multiburden	Lower middle	96.2
North Korea	6,226,704	0.50	Excess injury	5,778,666	0.49	Excess injury	Low	-7.2
Northern Mariana Islands	11,298	0.71	NCD	26,768	0.80	NCD	High	136.9
Norway	918,586	0.85	NCD	989,156	0.92	NCD	High	7.7
Oman	506,558	0.23	Excess injury	949,691	0.72	Excess injury	High	87.5
Pakistan	32,855,126	0.30	Multiburden	57,587,744	0.52	Multiburden	Lower middle	75.3

Palestine	699,874	0.41	Excess injury	1,594,819	0.45	NCD	Lower middle	127.9
Panama	793,910	0.60	Excess injury	1,016,448	0.75	Excess injury	Upper middle	28.0
Papua New Guinea	1,414,601	0.30	Multiburden	2,443,235	0.42	Multiburden	Lower middle	72.7
Paraguay	1,304,900	0.47	NCD	1,993,741	0.67	Excess injury	Upper middle	52.8
Peru	7,098,725	0.55	Multiburden	8,407,486	0.68	NCD	Upper middle	18.4
Philippines	19,974,190	0.52	Multiburden	30,160,568	0.64	Multiburden	Lower middle	51.0
Poland	8,544,622	0.73	Excess injury	6,092,748	0.87	NCD	High	-28.7
Portugal	2,411,011	0.69	Excess injury	1,649,366	0.80	NCD	High	-31.6
Puerto Rico	951,583	0.77	Excess injury	788,811	0.87	Excess injury	High	-17.1
Qatar	100,570	0.62	Excess injury	449,692	0.79	NCD	High	347.1
Romania	5,938,974	0.73	Excess injury	3,060,972	0.84	NCD	Upper middle	-48.5
Russia	30,491,032	0.77	Excess injury	21,959,644	0.83	Excess injury	Upper middle	-28.0
Rwanda	2,304,423	0.18	Multiburden	3,765,757	0.39	Multiburden	Low	63.4
Saint Lucia	43,456	0.55	Multiburden	46,981	0.71	Excess injury	Upper middle	8.1
Saint Vincent and the Grenadines	36,077	0.52	Excess injury	28,211	0.67	Excess injury	Upper middle	-21.8
Samoa	58,507	0.45	Multiburden	61,037	0.56	NCD	Lower middle	4.3
Sao Tome and Principe	39,372	0.29	Multiburden	62,804	0.43	Multiburden	Lower middle	59.5
Saudi Arabia	4,800,404	0.37	Excess injury	7,839,462	0.80	NCD	High	63.3
Senegal	2,444,043	0.24	Multiburden	4,941,166	0.35	Multiburden	Low	102.2
Serbia	2,181,640	0.70	Excess injury	1,617,168	0.77	NCD	Upper middle	-25.9
Seychelles	22,458	0.60	Excess injury	20,340	0.74	NCD	High	-9.4
Sierra Leone	1,186,556	0.22	Multiburden	2,152,170	0.35	Multiburden	Low	81.4
Singapore	660,800	0.74	NCD	765,330	0.89	NCD	High	15.8
Slovakia	1,278,537	0.78	Excess injury	890,128	0.88	NCD	High	-30.4
Slovenia	447,741	0.81	Excess injury	286,644	0.88	NCD	High	-36.0
Solomon Islands	105,612	0.32	Multiburden	190,228	0.44	Multiburden	Lower middle	80.1
Somalia	1,930,469	0.19	Multiburden	3,653,360	0.27	Multiburden	Low	89.2
South Africa	12,321,688	0.60	Multiburden	14,922,184	0.73	Multiburden	Upper middle	21.1
South Korea	12,767,286	0.74	Excess injury	9,057,533	0.88	NCD	High	-29.1
South Sudan	1,809,939	0.08	Multiburden	4,154,082	0.19	Multiburden	Low	129.5
Spain	9,715,801	0.75	Excess injury	6,728,578	0.85	NCD	High	-30.7
Sri Lanka	5,226,844	0.51	Multiburden	4,831,243	0.70	NCD	Lower middle	-7.6
Sudan	6,582,336	0.29	Multiburden	13,247,967	0.52	Multiburden	Lower middle	101.3
Suriname	122,473	0.57	Excess injury	139,605	0.70	Excess injury	Upper middle	14.0
Swaziland	280,927	0.45	Multiburden	443,862	0.59	Multiburden	Lower middle	58.0
Sweden	1,668,271	0.81	NCD	1,737,525	0.87	NCD	High	4.2
Switzerland	1,312,241	0.87	Excess injury	1,341,980	0.90	NCD	High	2.3
Syria	4,270,410	0.38	NCD	6,099,035	0.64	Excess injury	Lower middle	42.8
Taiwan	5,717,931	0.73	Excess injury	4,327,055	0.88	NCD	High	-24.3
Tajikistan	1,610,983	0.52	Multiburden	2,543,344	0.58	NCD	Lower middle	57.9
Tanzania	8,220,052	0.25	Multiburden	17,560,448	0.43	Multiburden	Low	113.6
Thailand	18,043,852	0.54	Multiburden	12,993,524	0.73	Excess injury	Upper middle	-28.0
The Bahamas	77,727	0.72	Excess injury	88,639	0.78	Excess injury	High	14.0
The Gambia	293,811	0.25	Multiburden	663,185	0.35	Multiburden	Low	125.7
Timor-Leste	230,034	0.32	Multiburden	385,945	0.53	Multiburden	Lower middle	67.8

Togo	1,222,669	0.25	Multiburden	2,377,746	0.40	Multiburden	Low	94.5
Tonga	33,078	0.45	Multiburden	34,409	0.59	Multiburden	Lower middle	4.0
Trinidad and Tobago	346,778	0.65	Excess injury	269,724	0.77	Excess injury	High	-22.2
Tunisia	2,577,675	0.48	Excess injury	2,510,657	0.70	NCD	Lower middle	-2.6
Turkey	16,825,298	0.58	Excess injury	19,870,272	0.76	NCD	Upper middle	18.1
Turkmenistan	1,135,154	0.58	Multiburden	1,495,568	0.76	NCD	Upper middle	31.8
Uganda	5,594,884	0.18	Multiburden	13,636,716	0.38	Multiburden	Low	143.7
Ukraine	10,583,102	0.75	Excess injury	6,559,694	0.79	NCD	Lower middle	-38.0
United Arab Emirates	427,960	0.56	Excess injury	1,556,415	0.77	Excess injury	High	263.7
United Kingdom	11,843,343	0.76	NCD	11,846,610	0.86	NCD	High	0.0
United States	54,099,292	0.85	Excess injury	64,615,120	0.90	NCD	High	19.4
Uruguay	766,964	0.64	Excess injury	765,600	0.75	NCD	High	-0.2
Uzbekistan	6,273,705	0.54	Multiburden	8,020,136	0.70	NCD	Lower middle	27.8
Vanuatu	45,698	0.34	Multiburden	78,571	0.48	Multiburden	Lower middle	71.9
Venezuela	6,240,441	0.59	Excess injury	8,394,964	0.74	Excess injury	Upper middle	34.5
Vietnam	21,617,560	0.44	Multiburden	21,947,848	0.64	Excess injury	Lower middle	1.5
Virgin Islands, U.S.	27,428	0.78	Excess injury	20,966	0.88	Excess injury	High	-23.6
Yemen	3,813,004	0.17	Multiburden	9,302,930	0.45	Multiburden	Lower middle	144.0
Zambia	2,697,374	0.30	Multiburden	5,523,037	0.46	Multiburden	Lower middle	104.8
Zimbabwe	3,505,384	0.43	Multiburden	5,150,313	0.50	Multiburden	Low	46.9

*Democratic Republic of the Congo
ǀ Federated States of Micronesia

Table 4b. This table summarises the distribution of World Bank Income groups (2016) and the 2016 Country groupings as defined by the Lancet Commission for the 195 countries

World Bank Income Group 2016	2016 Country Grouping		
	Multi-burden	Injury Excess	NCD predominant
Low	30	1	0
Lower-middle	31	6	14
Upper-middle	9	23	22
High	0	7	52

Population distributions by World Bank Income grouping and gender, for 1990 and 2016.

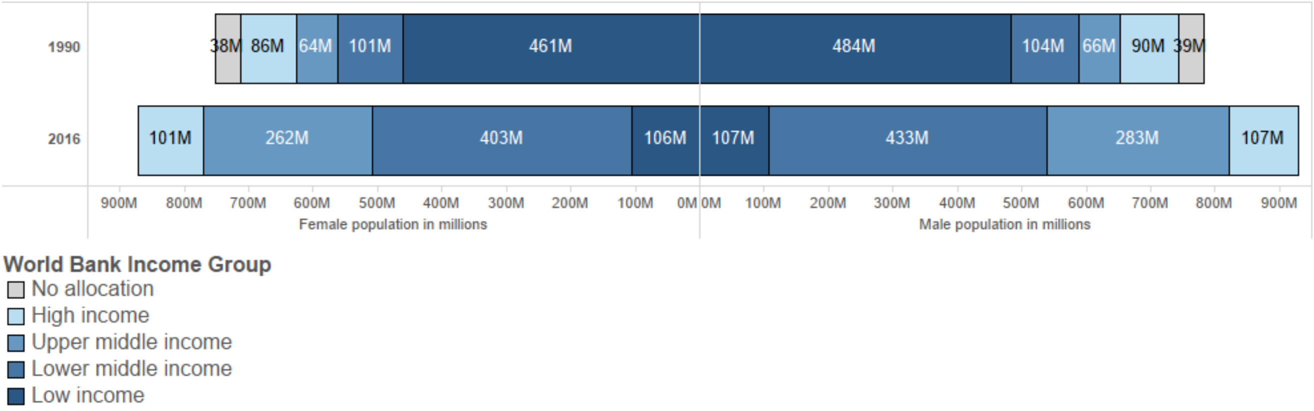

Figure 4a. Observed population distributions for China and India combined, by country group and gender, for 1990 and 2016.

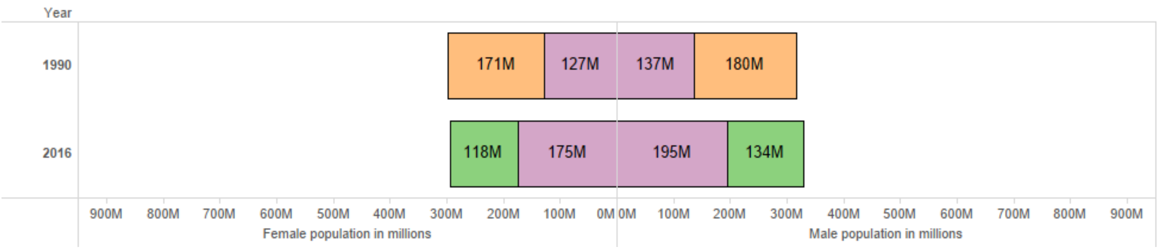

Figure 4b. Population distributions for scenario 1 (fixed demography, changing epidemiology), scenario 2 (changing demography, fixed epidemiology)

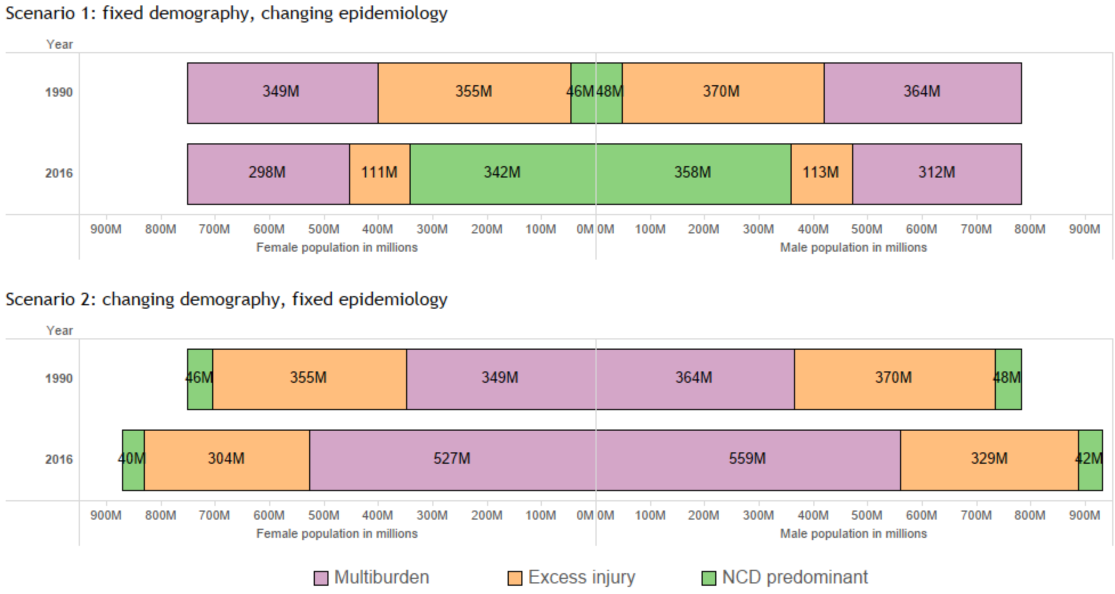

Appendix 5 – Counts of the indicators for the three country groups for two time points (typically 1990 and 2016)

This figure provides counts for each indicator, by gender for the three country groups. To the left of the dotted line, for the health outcomes are the counts of disability-adjusted life years lost (DALYs), to the right of the dotted line for risks and determinants are population counts. Counts for each indicator are shown for two time points, typically 1990 and 2016. Adolescent livebirths may be an overestimate as it is not adjusted for multiple births.

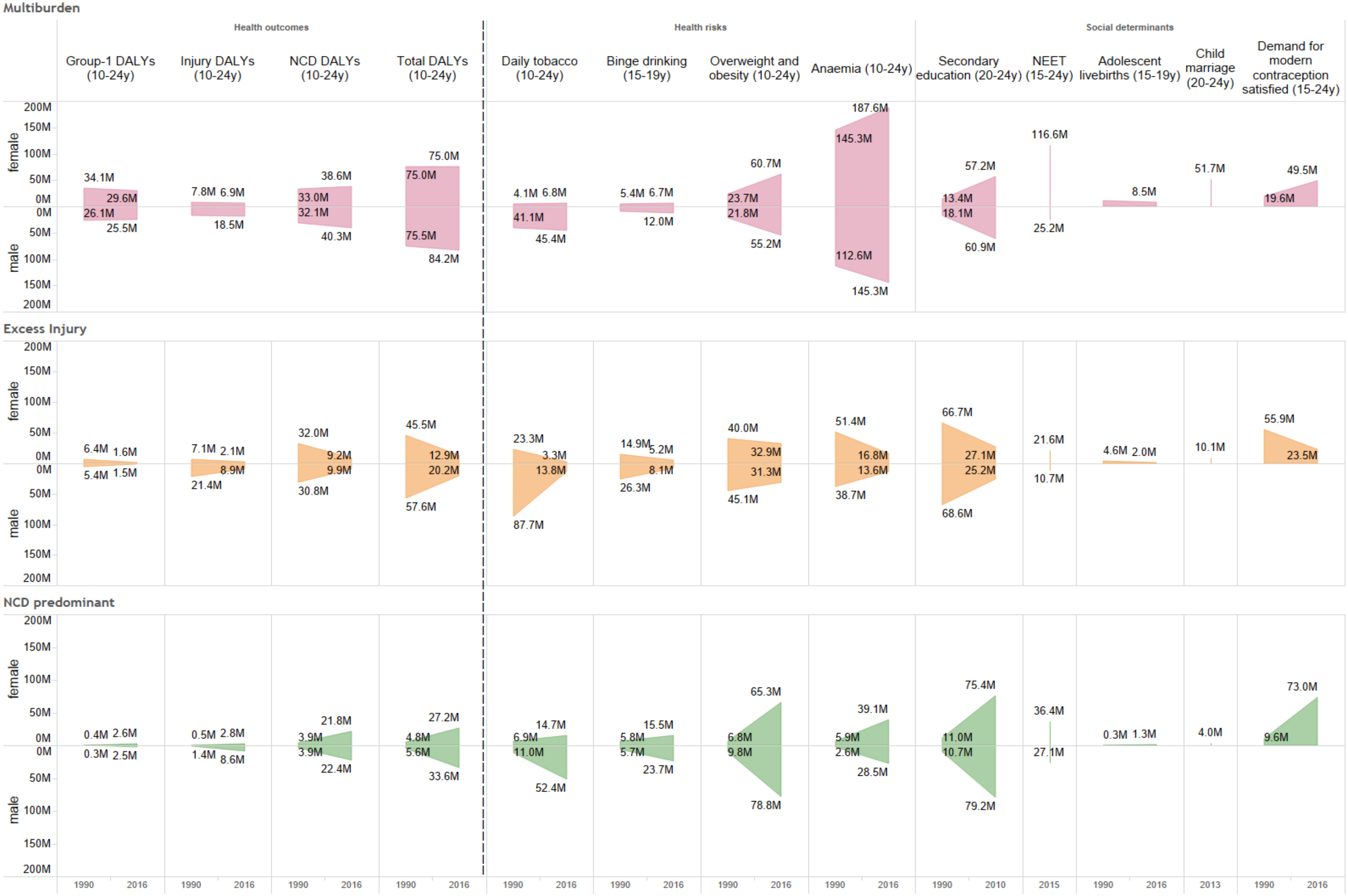

Appendix 5.1 Scenario 1 (global counts- fixed demography, changing epidemiology)

The global count (calculated as the sum of individual countries) in millions of each indicator (by gender) for the hypothetical scenario of the population remaining unchanged from 1990 but the indicator rates changing as observed in this study. To the left of the dotted line, for the health outcomes are the counts of disability-adjusted life years lost (DALYs), to the right of the dotted line for risks and determinants are population counts.

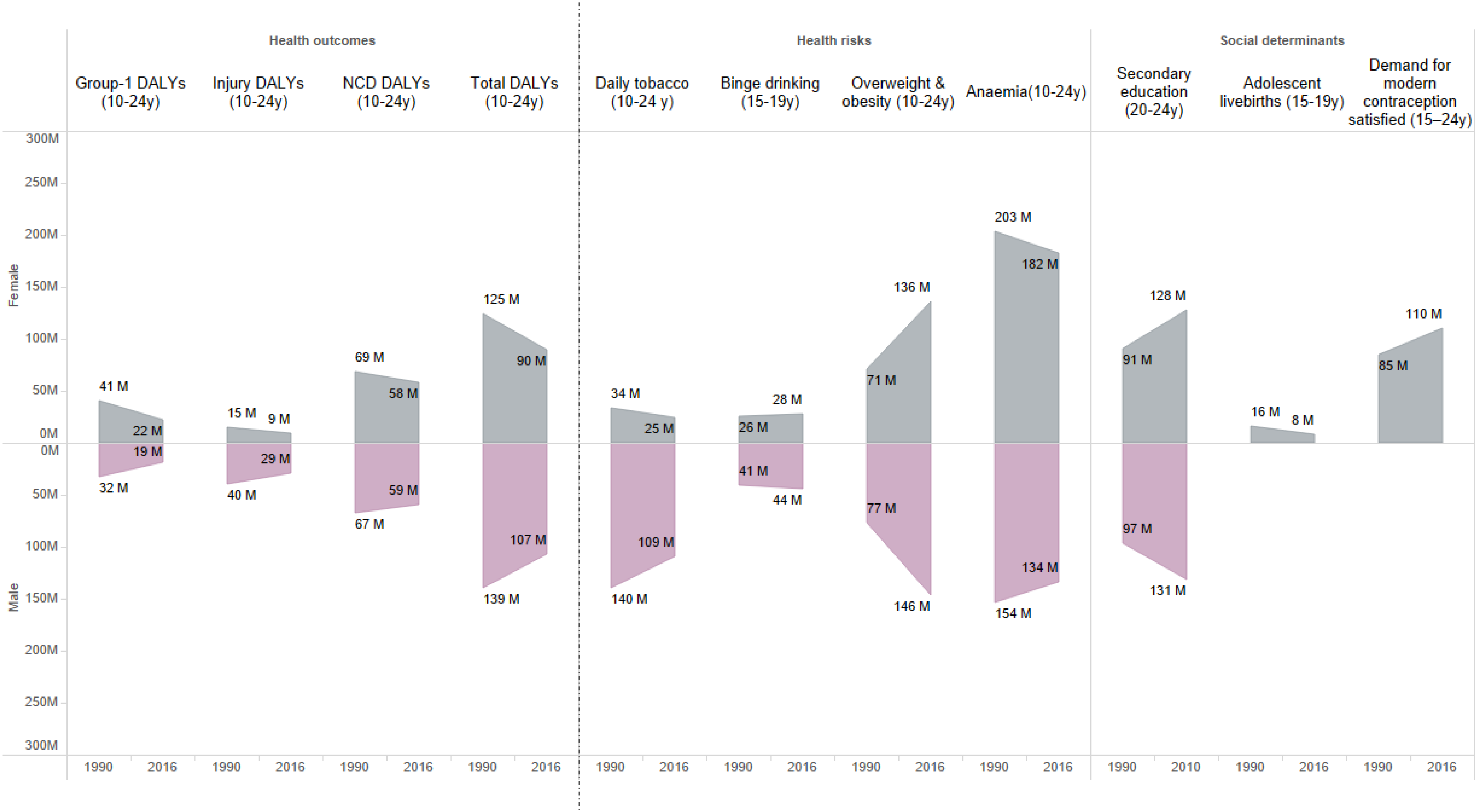

Appendix 5.2 Scenario 2 (global counts- changing demography, fixed epidemiology)
The global count (calculated as the sum of individual countries) in millions of each indicator (by gender) for the hypothetical scenario of the epidemiology (indicator prevalence or rates) remaining unchanged from 1990 but the demography changing as observed in this study. To the left of the dotted line, for the health outcomes are the counts of disability-adjusted life years lost (DALYs), to the right of the dotted line for risks and determinants are population counts.

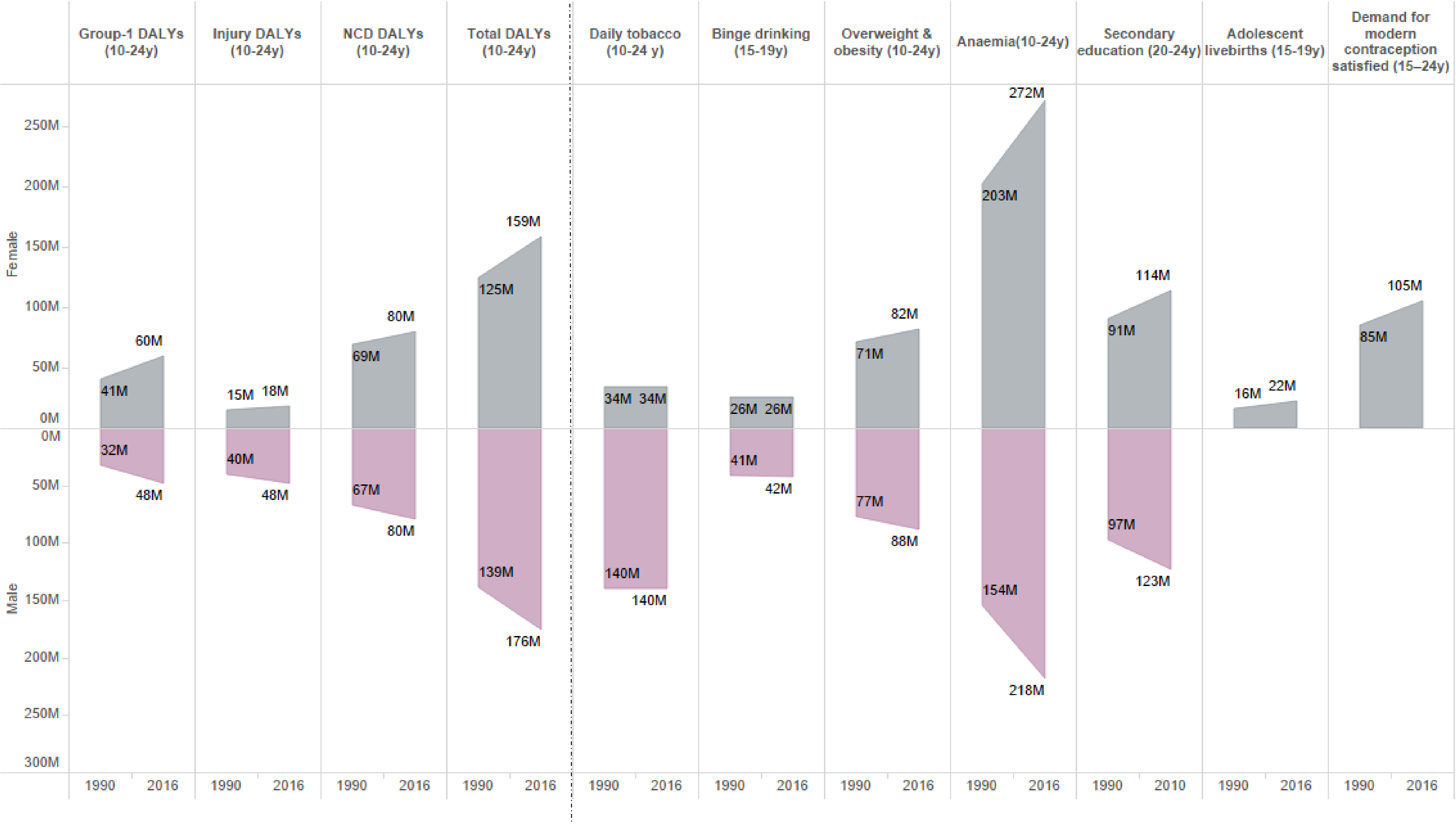

Appendix 5.3 - Counts for China and India combined.

The count calculated as the sum of China and India in millions of each indicator (by gender). To the left of the dotted line, for the health outcomes are the counts of disability-adjusted life years lost (DALYs), to the right of the dotted line for risks and determinants are population counts. Note that the indicators for NEET and Child Marriage are not included as they are not available for both locations

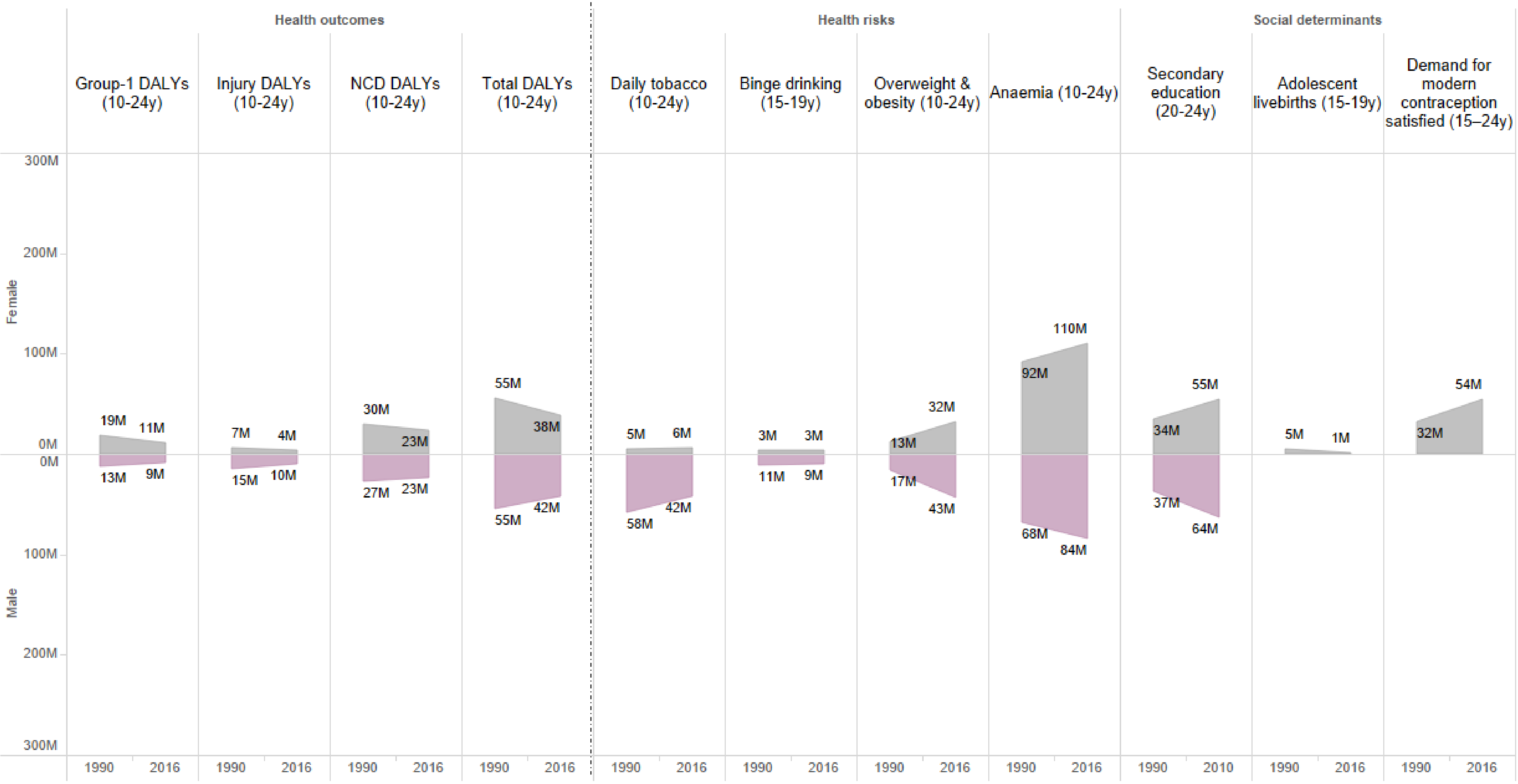

Appendix 6 – The distribution of indicator estimates, by country group

Each country in the three groups (Multi-burden in purple, injury excess in orange, NCD predominant in green) is represented by a line in the figure. The line is located at the individual country's estimate for that indicator, stratified by sex.

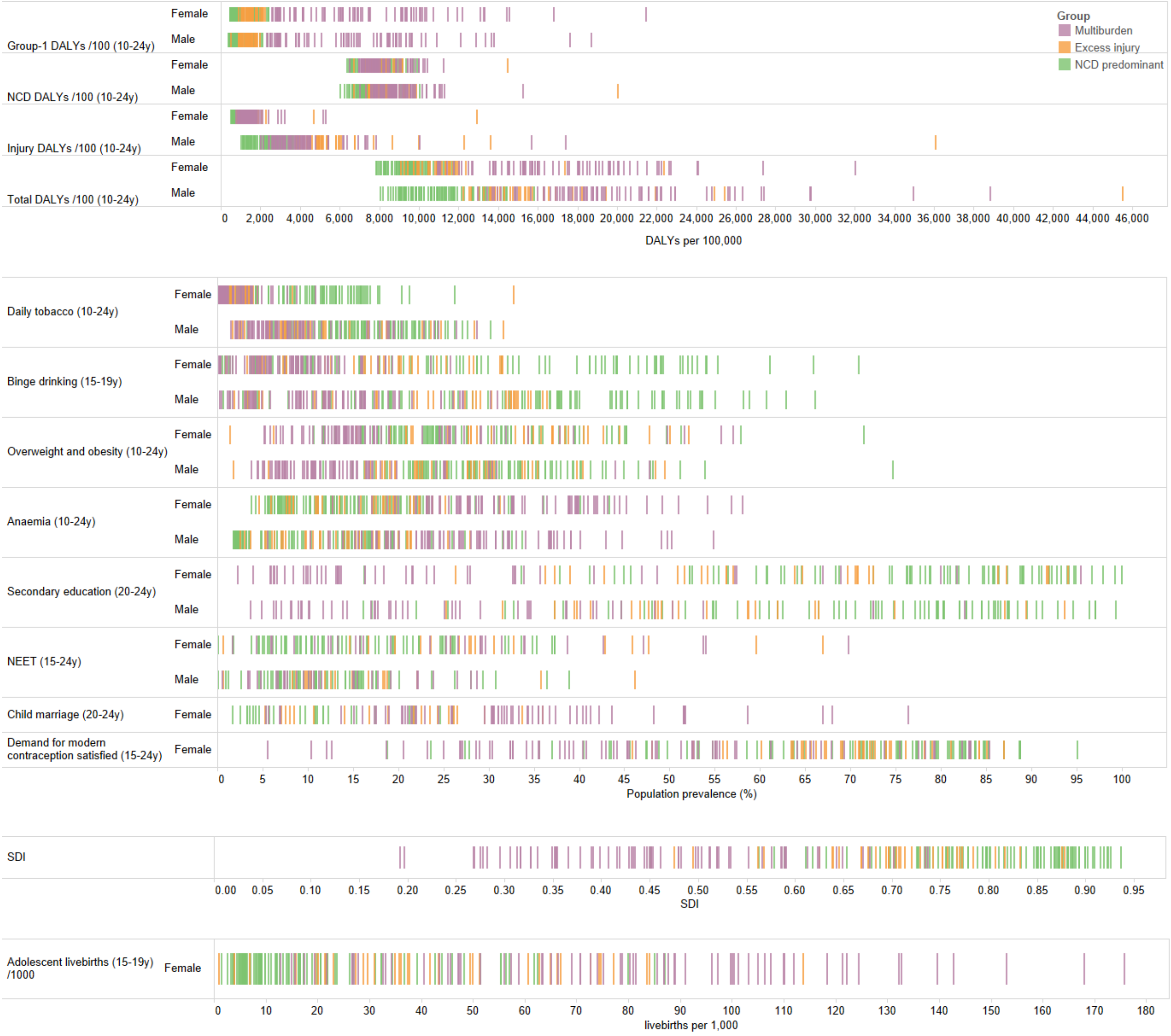

Appendix 7 – Rates of change for the indicators by country

For each indicator, annualised rate of change is shaded from green (improving) to red (declining).

-15.00 5.00

Panel A: Multi-burden countries

	Health outcomes								Health risks								Social determinants			
	Group-1 DALYs (10-24y)		Injury DALYs (10-24y)		NCD DALYs (10-24y)		Total DALYs (10-24y)		Daily tobacco (10-24y)		Binge drinking (15-19y)		Overweight and obesity (10-24y)		Anaemia (10-24y)		Secondary education (20-24y)		Adolescent livebirths (15- 19y)	Demand for modern contraception satisfied (15-24y)
	Female	Male	Female	Male	Female	Male	Female	Male	Female	Male	Female	Male	Female	Male	Female	Male	Female	Male	Female	Female
Afghanistan	-2.13	-2.15	-0.39	0.29	-0.17	-0.08	-1.06	-0.39	0.04	0.37	-0.31	-0.22	0.85	0.62	-1.01	-1.45	4.87	5.21	-2.04	12.60
Angola	-2.11	-2.21	-1.86	-1.81	-0.34	-0.42	-1.51	-1.60	0.83	1.80	-0.18	-0.09	7.11	5.63	-1.12	-1.24			-1.02	12.88
Bangladesh	-2.64	-2.53	-1.43	0.00	-1.09	-0.87	-1.81	-1.30	0.04	0.23	0.91	0.59	8.53	12.57	-1.16	-1.83	24.63	9.57	-1.69	2.30
Benin	-1.23	-1.21	-0.81	-0.60	-0.40	-0.35	-0.82	-0.75	2.18	-1.34	-0.23	0.63	12.50	5.66	-0.51	-0.57	12.18	3.19	-0.90	10.82
Bhutan	-2.40	-1.97	-2.72	-1.82	-0.76	-0.35	-1.75	-1.19	-1.01	-1.39	0.58	0.46	8.45	9.39	-0.51	-1.03			-3.60	5.86
Botswana	-2.65	-2.22	-0.28	0.10	-0.05	-0.28	-1.22	-0.87	-0.09	0.53	-0.07	-0.02	4.60	2.66	-0.75	-1.41	-0.99	-0.34	-2.52	2.77
Burkina Faso	-1.40	-1.47	-0.60	-0.46	-0.43	-0.37	-0.96	-0.94	-3.69	-0.29	0.06	-0.06	15.14	3.17	-0.45	-0.48			-0.99	14.61
Burundi	-1.96	-1.82	-2.31	-2.29	-0.45	-0.50	-1.52	-1.47	-1.21	-0.10	0.01	-0.03	0.10	0.27	-0.89	-1.07	11.16	6.28	-1.66	42.80
Cambodia	-2.51	-2.18	-2.28	-0.84	-0.50	-0.15	-1.69	-1.09	-1.61	-0.57	-0.11	0.15	7.63	4.07	-1.09	-1.36	19.12	6.00	0.07	9.36
Cameroon	-0.49	-0.41	-0.42	0.46	-0.21	0.28	-0.37	0.00	-1.62	-0.86	0.47	0.64	2.86	2.92	-0.48	-0.64	3.38	1.18	-1.45	23.95
Cape Verde	-1.68	-1.74	-0.28	0.78	-0.24	0.07	-0.77	-0.27	-0.44	0.24	0.05	0.30	5.59	5.36	-0.71	-1.17			-0.13	0.50
Central African Repub..	-0.30	-0.35	0.33	0.50	0.04	0.08	-0.15	-0.06	0.74	1.73	-0.44	-0.30	2.71	-0.51	-0.39	-0.44	0.72	0.70	-1.06	5.02
Chad	-0.76	-0.88	-1.22	-0.91	-0.17	0.06	-0.59	-0.57	-0.84	-0.16	-1.37	-0.58	4.15	2.39	-0.34	-0.22			-1.41	3.15
Comoros	-1.72	-1.46	-1.26	-1.12	-0.41	-0.40	-1.11	-0.93	-0.14	0.88	0.11	0.05	2.60	0.86	-0.68	-1.18			-1.95	2.69
Congo	-1.20	-1.59	-1.22	-1.78	-0.30	-0.52	-0.90	-1.30	1.69	2.03	-0.29	-0.56	0.48	2.13	-0.61	-0.76	-4.46	-3.65	-0.73	7.28
Cote d'Ivoire	-0.91	-0.92	-0.57	-0.34	-0.16	-0.03	-0.60	-0.50	-0.27	0.56	0.27	-0.05	3.80	10.06	-0.39	-0.46	16.78	5.19	-0.04	9.12
Democratic Republic ..	-1.38	-1.23	-1.08	-0.39	-0.24	0.00	-0.98	-0.69	0.81	-0.02	-0.27	-0.21	-0.90	-1.91	-0.52	-0.33	-1.32	-1.70	0.25	8.75
Djibouti	-0.93	-0.77	-1.38	-0.85	-0.14	-0.09	-0.64	-0.50	-0.13	0.08	0.18	0.16	11.09	9.71	-0.62	-0.39			-1.58	7.42
Equatorial Guinea	-1.90	-1.90	-2.15	-2.33	-0.90	-1.10	-1.61	-1.77	-0.12	0.00	0.03	-0.13	11.25	9.53	-1.35	-1.66			-1.39	15.92
Eritrea	-1.61	-1.43	-1.57	-1.42	-0.28	-0.38	-1.17	-1.05	-1.65	-1.12	0.08	-0.05	5.92	1.82	-1.15	-0.98			-2.25	6.02
Ethiopia	-2.45	-2.24	-2.57	-2.48	-0.61	-0.57	-1.99	-1.95	-0.09	0.50	2.33	3.63	7.57	17.30	-1.19	-1.42			-1.79	16.78
Federated States of ..	-1.28	-1.29	0.40	-0.01	0.07	-0.07	-0.28	-0.28	0.35	0.18	0.25	-0.20	1.81	1.93	-0.61	-0.42			-2.99	2.87
Fiji	-1.05	-1.37	0.58	-0.09	0.30	0.02	-0.02	-0.30	0.64	-0.35	-0.07	-0.40	1.18	1.35	-0.70	-0.87	1.00	0.80	-1.60	1.46
Gabon	-1.26	-1.40	-0.61	-1.38	-0.01	-0.37	-0.70	-1.03	0.41	0.68	-0.40	-0.38	4.33	3.06	-0.41	-0.80	1.86	1.55	-1.61	7.07
Ghana	-1.35	-0.96	-1.03	-0.36	-0.53	0.02	-0.99	-0.47	-1.70	0.10	0.59	0.20	11.20	1.79	-0.60	-0.76	8.06	4.25	-1.16	12.11
Guinea	-1.15	-0.84	-1.11	-0.24	-0.35	0.06	-0.84	-0.40	0.00	-0.32	0.24	0.51	4.13	1.82	-0.29	-0.36			-0.45	6.54
Guinea-Bissau	-0.95	-0.99	-1.20	-0.76	-0.47	-0.25	-0.80	-0.68	-0.64	-0.12	-0.04	0.22	5.48	5.88	-0.27	-0.40			-1.18	25.16
Guyana	-0.95	-1.07	0.15	0.34	0.02	-0.01	-0.19	-0.07	-2.32	-0.92	-0.63	-0.35	2.61	2.48	-0.73	-0.91	3.79	4.37	-0.68	-1.07
Haiti	-0.94	-0.35	26.51	19.56	-0.78	-0.38	3.18	6.94	-0.39	-0.20	-0.69	0.12	1.96	2.70	-0.51	-0.87	5.15	0.24	-1.35	6.70
India	-1.90	-1.73	-1.35	-0.83	-0.74	-0.46	-1.35	-0.99	6.37	0.37	-0.53	0.37	8.48	6.23	0.00	-0.02	46.46	28.28	-3.16	3.66
Indonesia	-1.96	-1.87	-1.76	0.40	-0.37	-0.42	-1.20	-0.78	1.71	0.37	0.50	-1.11	6.82	8.77	-0.07	-1.38	7.77	4.88	-1.12	0.36
Kenya	-1.03	-0.13	-0.96	0.27	-0.15	0.18	-0.70	0.04	-2.44	-1.51	-0.91	-0.89	3.63	-0.23	0.33	0.35	16.01	8.33	-1.38	1.45
Kiribati	-1.48	-1.26	-0.54	-0.63	0.01	0.24	-0.62	-0.24	-0.79	0.97	0.24	0.11	1.53	1.47	-0.51	-0.29			-3.08	3.37
Laos	-2.62	-2.52	-1.47	-0.99	-0.70	-0.60	-1.67	-1.30	0.27	-0.02	-0.45	-0.63	6.82	8.13	-1.08	-1.51	5.95	1.28	-0.36	3.47
Lesotho	6.83	6.17	0.30	1.47	0.24	0.65	3.09	2.63	-1.49	2.39	-0.05	0.02	2.34	2.34	-0.02	-0.89	0.01	-0.05	0.74	2.84
Liberia	-1.70	-1.43	-3.06	-3.06	-0.46	-0.25	-1.73	-2.07	-2.78	-1.76	0.22	1.20	1.82	1.63	-0.47	-0.82	4.11	0.10	-1.44	15.92
Madagascar	-1.36	-1.09	-0.80	-0.69	-0.32	-0.36	-0.90	-0.70	-0.82	-0.74	-0.04	-0.08	0.09	0.99	-0.89	-0.83			-0.96	16.46
Malawi	-1.01	-0.16	-1.91	-0.38	-0.36	0.47	-0.88	0.02	0.97	-0.08	-1.04	-1.22	2.79	1.19	-0.82	-0.96	32.91	8.96	-0.63	17.46
Mali	-1.66	-1.53	-1.62	-1.26	-0.68	-0.45	-1.32	-1.11	-2.18	-0.95	0.03	0.40	10.03	7.06	-0.48	-0.52	32.05	10.89	-0.42	14.54
Marshall Islands	-0.86	-0.91	0.69	0.01	0.23	-0.10	-0.01	-0.21	0.75	-0.83	0.02	-0.12	1.44	1.39	-0.48	-0.34			-1.99	1.32
Mauritania	-1.90	-1.83	-1.58	-1.73	-0.38	-0.51	-1.25	-1.27	-0.15	-0.09	-0.12	0.49	4.26	2.06	-0.65	-1.02	7.02	1.16	-1.14	1.56
Mozambique	-0.04	0.41	-2.01	-1.21	-0.48	-0.23	-0.39	-0.18	-0.10	-0.20	0.19	0.12	5.72	3.40	-0.45	-0.71	19.01	7.18	-0.75	10.07
Myanmar	-2.56	-2.43	-2.05	-0.71	-0.83	-0.48	-1.73	-1.31	-0.10	-0.83	-0.09	0.18	13.02	12.62	-1.57	-1.77	6.46	11.38	-1.93	4.91
Namibia	-0.60	-0.37	-1.68	-0.35	-0.37	-0.20	-0.64	-0.30	-0.86	0.08	0.45	1.58	2.46	1.49	-0.99	-1.17	-0.67	0.52	-1.13	3.18
Nepal	-2.62	-2.31	-0.16	0.55	-0.68	-0.47	-1.63	-0.99	-2.77	-0.81	8.39	0.96	24.35	10.82	-1.17	-1.44	9.12	-0.57	-1.10	4.66
Niger	-1.36	-1.36	-1.07	-0.82	-0.40	-0.29	-0.98	-0.90	-2.12	1.13	-0.04	0.22	4.98	2.87	-0.43	-0.24	15.95	-0.22	-0.52	12.07
Nigeria	-1.38	-1.08	-1.25	-0.54	-0.79	-0.37	-1.11	-0.73	-3.09	-2.69	0.71	0.48	5.34	2.63	-0.76	-0.73			-0.94	10.74
Pakistan	-1.87	-1.69	0.21	1.02	0.03	0.09	-0.85	-0.31	-1.28	-0.53	0.34	0.06	6.23	3.84	-0.43	-0.66	8.50	0.32	-2.07	9.33
Papua New Guinea	-1.27	-1.21	-0.35	-0.54	-0.15	-0.23	-0.63	-0.57	0.30	-0.44	-0.22	-0.32	1.41	0.48	-0.40	-0.83	1.89	-1.28	-0.95	2.44
Philippines	-1.42	-1.49	-0.90	-0.36	-0.02	-0.42	-0.55	-0.68	-0.56	-0.71	-0.21	-0.84	4.00	4.79	-1.05	-1.61	1.66	0.86	1.48	2.19
Rwanda	-2.77	-2.88	-4.11	-4.19	-0.65	-0.60	-2.30	-2.60	4.75	-1.07	-0.24	-0.50	1.41	-0.11	-1.21	-1.75	12.76	17.02	-2.21	13.37
Sao Tome and Princi..	-2.08	-1.42	-1.21	-0.43	-0.70	-0.24	-1.27	-0.65	-0.69	0.39	0.52	0.67	2.88	2.45	-0.48	-0.54			-0.87	2.26
Senegal	-1.90	-1.56	-1.34	-0.56	-0.42	0.01	-1.27	-0.77	-2.53	-1.90	-1.07	-0.61	3.25	0.58	-0.43	-0.47	0.92	-1.69	-1.43	37.49
Sierra Leone	-0.16	-0.76	-5.36	-5.69	0.04	-0.49	-0.36	-1.35	-1.26	-1.18	0.15	0.44	2.54	2.28	-0.53	-0.51	19.01	7.20	-0.77	20.05
Solomon Islands	-1.74	-1.95	1.25	0.39	0.48	0.05	-0.36	-0.46	-0.60	-0.43	-0.04	-0.13	2.30	1.84	-0.47	-0.62			-1.73	2.05
Somalia	-1.14	-0.93	-0.56	-0.49	-0.24	-0.22	-0.78	-0.58	-1.54	-1.00	-0.05	0.01	1.09	0.17	0.35	0.30			-0.41	5.45
South Africa	4.24	6.24	0.09	-0.44	0.41	0.12	1.57	0.91	-2.84	-1.61	0.07	-0.28	4.02	2.64	0.79	0.56	8.92	7.76	-1.87	0.64
South Sudan	-1.20	-1.11	-1.74	-1.70	-0.18	-0.03	-1.02	-1.06	-1.42	-0.72	0.00	-0.09	0.92	0.59	-0.12	-0.62			-1.84	2.75
Sudan	-2.07	-1.89	-1.00	-0.82	-0.56	-0.28	-1.21	-0.86	0.05	-0.18	0.10	-0.05	4.42	9.24	-0.71	-0.72	2.36	0.11	-1.88	7.42
Swaziland	4.93	5.23	-0.65	0.18	0.03	0.50	1.77	1.60	-0.75	-0.20	-0.27	-0.15	0.03	1.17	0.56	-0.07	-0.74	1.24	-1.11	3.36
Tanzania	-1.39	-0.83	-1.40	-0.54	-0.43	-0.14	-1.07	-0.53	1.03	-0.58	0.17	0.09	2.72	0.74	-0.97	-1.17	20.83	2.80	-1.05	9.50
The Gambia	-1.27	-0.99	-1.32	-0.32	-0.50	-0.02	-0.93	-0.47	-1.34	-1.05	0.12	0.32	0.40	1.48	-0.34	-0.42	1.67	0.62	-1.32	4.71
Timor-Leste	-2.74	-2.26	-3.09	-3.01	-1.06	-0.70	-2.06	-1.86	0.36	-1.53	-0.37	-0.32	10.60	-0.95	-1.31	-1.33			-0.70	2.43
Togo	-0.73	0.01	-1.19	-0.15	-0.51	0.01	-0.66	-0.02	-2.61	-0.36	-0.14	0.12	11.45	5.71	-0.40	-0.91	5.59	2.91	-0.67	22.68
Tonga	-1.00	-0.88	-0.73	-0.51	-0.07	0.15	-0.42	-0.25	-0.46	-0.81	0.02	-0.02	1.63	1.23	-0.50	-0.57	10.30	8.25	-1.88	2.27
Uganda	-1.56	-1.42	-1.15	-0.59	-0.31	0.03	-1.18	-0.90	-0.69	-1.50	-0.14	-0.68	3.77	0.64	-0.88	-0.99	13.58	5.10	-1.95	19.72
Vanuatu	-1.11	-1.41	1.28	0.63	0.31	0.13	-0.15	-0.17	1.48	-0.27	-0.20	-0.24	0.98	2.81	-0.26	-0.56			-1.49	3.35
Yemen	-1.96	-1.69	2.30	2.90	-0.58	-0.28	-0.79	0.55	-0.48	-0.44	-0.10	-0.13	0.11	4.09	-0.19	-0.32	49.32	3.40	-1.91	24.16
Zambia	-0.94	0.50	-1.70	1.36	-0.60	0.68	-0.90	0.67	1.49	-0.28	-1.95	-2.24	3.62	1.08	-0.63	-0.51	1.21	-1.09	-1.93	8.55
Zimbabwe	1.52	2.11	-0.94	0.91	0.37	1.18	0.82	1.53	-0.45	0.71	0.41	-0.79	1.59	-1.34	-0.33	-0.68	-4.64	-4.73	0.41	1.14

Panel B: Injury excess countries

	Health outcomes								Health risks								Social determinants			
	Group-1 DALYs (10-24y)		Injury DALYs (10-24y)		NCD DALYs (10-24y)		Total DALYs (10-24y)		Daily tobacco (10-24y)		Binge drinking (15-19y)		Overweight and obesity (10-24y)		Anaemia (10-24y)		Secondary education (20-24y)		Adolescent livebirths (15- 19y)	Demand for modern contraception satisfied (15-24y)
	Female	Male	Female	Male	Female	Male	Female	Male	Female	Male	Female	Male	Female	Male	Female	Male	Female	Male	Female	Female
Belize	-0.67	-0.58	-0.56	0.22	-0.03	0.01	-0.22	0.01	2.50	0.29	0.14	-0.04	2.56	2.99	-0.82	-0.63	8.70	8.10	-1.50	1.36
Brazil	-1.01	-1.18	-1.15	0.27	-0.08	0.12	-0.39	0.06	-2.96	-2.38	0.75	0.67	4.24	5.16	-0.21	-0.40	7.09	8.73	-0.81	0.90
Colombia	-1.45	-1.33	-1.96	-2.28	-0.22	-0.32	-0.73	-1.53	-1.81	-0.68	-1.64	-1.30	1.26	3.65	-1.02	-1.28	8.21	7.86	-2.10	1.89
Dominica	0.15	-0.28	4.95	2.62	0.09	0.04	0.49	0.72	-2.80	-2.38	-0.45	-0.31	2.34	2.79	-1.00	-0.30			-2.36	1.32
Dominican Republic	-0.81	-0.88	0.04	-0.52	-0.30	-0.48	-0.38	-0.55	-2.17	-2.29	-0.40	-0.47	3.21	3.21	-0.97	-1.35	5.43	5.60	-0.99	2.21
Ecuador	-2.04	-1.88	-0.48	-0.78	-0.41	-0.29	-0.77	-0.68	-1.11	-1.86	-0.31	-0.61	-0.16	1.29	-1.23	-1.43	1.70	1.31	-0.38	1.98
El Salvador	-1.84	-1.41	-1.44	-1.14	-0.61	-0.59	-0.97	-0.95	0.27	-0.29	-0.24	-0.14	3.42	4.82	-1.51	-1.55	6.76	7.29	-1.45	0.83
Georgia	-1.05	-0.95	-0.93	-0.33	0.02	0.38	-0.30	-0.04	-0.80	1.79	-0.41	-2.13	1.93	3.71	-0.30	0.03			-1.00	0.24
Greenland	-0.72	-1.22	-1.00	-2.25	-0.15	-1.45	-0.31	-1.67	-0.44	-0.30	0.14	-0.02	0.60	0.85	-0.28	-0.65			-2.47	-0.24
Grenada	-1.06	-0.83	0.25	0.88	0.10	0.30	-0.14	0.29	-0.75	2.35	-0.37	-0.27	3.16	3.54	-0.87	-1.08			-2.85	1.72
Guatemala	-2.41	-2.20	-0.68	0.50	-0.18	-0.08	-1.07	-0.38	0.86	1.19	-0.80	-0.53	4.24	4.32	-1.30	-1.42	2.99	2.55	-1.67	6.99
Honduras	-2.08	-1.71	-1.01	-0.57	-0.37	-0.43	-0.92	-0.66	-1.10	-0.16	0.38	1.28	3.38	3.86	-1.28	-1.55	4.81	3.52	-1.55	1.94
Iran	-1.41	-0.87	-2.16	-1.66	0.13	0.35	-0.83	-0.85	2.28	0.85	0.20	0.14	5.62	4.94	-1.33	-1.53	4.22	0.55	-2.16	2.27
Iraq	-1.19	-1.14	1.24	3.01	-0.30	0.04	-0.14	1.08	-1.22	-0.95	-0.09	-0.10	1.15	1.83	-0.06	-0.98	5.44	1.78	2.67	3.81
Jamaica	-0.64	-0.68	0.81	1.96	-0.13	-0.07	-0.15	0.32	1.65	0.89	-0.04	0.06	1.22	2.38	-0.52	-0.66	2.82	4.05	-1.79	0.83
Jordan	-1.33	-1.08	-0.64	-0.41	-0.35	-0.32	-0.55	-0.42	-0.97	1.26	0.06	-0.28	2.14	3.07	-0.77	-1.15	0.02	-1.21	-2.16	3.20
Kazakhstan	-1.48	-2.07	-1.11	-1.63	-0.42	-0.55	-0.74	-1.11	0.09	-0.58	1.28	1.01	0.14	1.10	-0.36	-0.01	0.00	0.31	-1.70	1.82
Libya	-0.94	-0.66	3.55	4.10	-0.25	-0.05	0.09	1.13	1.05	0.18	-0.04	0.00	1.73	2.59	-0.68	-0.31	8.52	1.94	-2.22	2.90
Mexico	-1.51	-1.38	-1.10	-1.11	-0.04	0.22	-0.43	-0.52	-1.98	-1.63	1.86	0.64	2.86	1.77	0.22	0.24	3.90	3.46	-1.42	0.90
Mongolia	-2.27	-1.43	-0.24	0.24	-0.56	0.00	-1.01	-0.14	3.39	-0.23	1.37	1.47	2.80	5.07	-0.54	-0.32	1.10	1.60	-2.21	2.61
North Korea	-4.08	-5.15	0.94	1.24	0.01	0.14	-0.67	-0.01	-0.93	-0.82	-0.33	-0.20	0.52	1.07	-1.27	-0.73			-2.72	-0.03
Oman	-2.07	-1.74	-1.75	-0.50	-0.11	0.11	-0.77	-0.35	-1.52	-0.38	0.08	0.06	8.07	13.66	-1.97	-1.91			-3.63	11.96
Panama	-0.77	0.30	-0.93	0.35	-0.08	0.24	-0.30	0.28	-1.53	-2.29	-0.47	-0.29	4.72	5.64	-1.28	-1.23	2.18	2.46	-1.13	1.11
Paraguay	-1.12	-0.65	0.06	1.52	0.15	0.31	-0.11	0.55	-1.34	-1.43	0.12	-0.28	4.81	5.70	-0.38	-0.51	5.96	5.71	-2.02	2.97
Puerto Rico	-0.96	-1.19	-1.63	-1.06	-0.22	-0.24	-0.49	-0.66	-0.43	-0.36	1.09	0.56	2.22	2.72	-0.73	-0.86			-1.93	0.69
Russia	-0.29	-0.49	-1.47	-2.23	-0.22	-0.44	-0.48	-1.19	-0.20	-1.33	1.43	0.69	1.32	3.35	-0.01	0.03	0.01	-0.09	-1.88	0.64
Saint Lucia	-1.19	-1.25	0.08	0.03	-0.13	-0.16	-0.36	-0.28	-0.25	1.36	-0.31	-0.35	1.25	0.29	-0.64	-0.96			-2.31	1.68
Saint Vincent and the ..	-0.89	-0.58	-0.20	1.03	-0.15	0.23	-0.30	0.37	0.44	-0.68	-0.10	-0.19	3.52	3.80	-0.94	-0.87			-1.80	1.90
Suriname	-0.80	-0.63	-0.85	0.16	-0.25	0.04	-0.41	-0.01	0.62	1.47	-0.12	-0.07	3.00	4.35	-0.83	-0.86			-1.59	0.56
Syria	-1.63	-0.98	42.78	38.04	-0.58	-0.72	2.59	7.54	-2.08	-1.49	0.29	0.25	1.89	3.10	-1.23	-0.95	7.24	5.24	-2.44	3.42
Thailand	-1.97	-2.15	-2.38	-2.30	-0.60	-0.78	-1.25	-1.62	-1.17	-0.75	-0.03	0.02	4.34	7.39	-1.49	-1.87	9.63	8.34	-1.53	0.45
The Bahamas	-0.65	-0.50	0.08	1.09	-0.19	0.25	-0.23	0.49	-0.12	-1.08	-0.30	-0.21	0.77	1.07	-0.44	-0.22			-2.20	0.66
Trinidad and Tobago	-1.02	-0.48	-0.04	1.49	-0.35	0.21	-0.42	0.57	2.58	0.12	0.24	0.11	3.16	5.26	-0.69	-1.20	12.03	13.43	-2.47	1.27
United Arab Emirates	-1.28	-0.96	-0.95	-0.44	-0.05	-0.12	-0.37	-0.30	0.82	0.58	-0.08	-0.13	1.89	2.04	-0.73	-1.04	0.57	0.89	-0.60	3.60
Venezuela	-0.61	-0.04	-0.03	3.42	-0.15	0.60	-0.20	1.79	-0.27	-0.03	-0.84	-0.69	2.29	2.83	-1.37	-1.11	6.28	7.05	-0.86	1.20
Vietnam	-2.02	-1.82	-1.47	-0.58	-0.28	-0.18	-0.94	-0.64	0.25	-1.50	0.28	1.06	9.47	7.09	-1.69	-2.17	31.63	25.32	1.59	0.83
Virgin Islands, U.S.	0.12	0.75	-1.02	-0.38	-0.18	-0.31	-0.24	-0.26	-2.30	-1.57	-0.29	-0.24	1.70	2.29	-0.85	-0.79			-2.32	0.66

Panel C: NCD predominant countries

	Health outcomes								Health risks								Social determinants			
	Group-1 DALYs (10-24y)		Injury DALYs (10-24y)		NCD DALYs (10-24y)		Total DALYs (10-24y)		Daily tobacco (10-24y)		Binge drinking (15-19y)		Overweight and obesity (10-24y)		Anaemia (10-24y)		Secondary education (20-24y)		Adolescent livebirths (15-19y)	Demand for modern contraception satisfied (15-24y)
	Female	Male	Female	Male	Female	Male	Female	Male	Female	Male	Female	Male	Female	Male	Female	Male	Female	Male	Female	Female
Albania	-1.61	-1.39	-1.40	-2.10	0.02	0.34	-0.38	-0.61	4.34	0.62	0.66	0.55	2.33	2.71	-1.32	-1.20	1.95	1.48	0.43	1.26
Algeria	-1.81	-1.31	-1.75	-1.77	-0.22	-0.20	-0.77	-0.85	-0.04	-0.02	-0.01	-0.03	7.38	6.50	-1.17	-1.01	5.00	0.20	-2.54	1.29
American Samoa	-0.92	-0.83	-0.57	-1.31	-0.31	-0.36	-0.46	-0.70	-1.02	-0.81	-0.19	-0.33	0.54	0.48	-1.10	-0.48			-2.60	1.38
Andorra	-0.44	-0.64	-0.82	-1.39	-0.10	-0.17	-0.18	-0.46	-1.30	-1.00	0.10	0.07	1.09	0.99	-0.44	-0.67			-1.17	0.18
Antigua and Barbuda	-0.54	-0.37	-0.77	0.90	-0.30	0.13	-0.37	0.26	0.67	-0.24	-0.36	-0.34	1.66	2.08	-0.71	-0.66			-1.63	0.80
Argentina	-0.88	-1.03	-0.67	-0.32	0.00	0.16	-0.17	-0.06	-0.69	-1.46	0.03	-0.39	3.01	2.77	-0.85	-1.06	0.38	0.40	-0.08	0.88
Armenia	-1.15	-1.34	-2.02	-2.40	-0.05	-0.12	-0.57	-1.19	-0.87	-0.16	0.88	-0.54	1.54	5.13	-0.52	-0.41	-0.21	-0.24	-2.45	2.17
Australia	-0.67	-0.78	-1.73	-2.19	-0.14	-0.59	-0.35	-1.00	-2.17	-1.88	0.05	1.03	2.10	1.65	-0.73	-0.71	-0.53	-0.81	-1.53	0.38
Austria	-0.56	-0.54	-1.76	-2.45	-0.10	-0.43	-0.34	-1.07	-0.56	-0.18	1.06	0.63	2.08	0.35	-0.82	-1.08	1.80	0.65	-2.62	1.29
Azerbaijan	-1.48	-1.47	-1.36	-1.78	-0.14	0.37	-0.55	-0.57	-0.77	0.68	0.11	-0.38	2.12	4.86	-0.23	-0.52			4.10	0.53
Bahrain	-0.94	-0.87	-1.80	-2.05	-0.40	-0.36	-0.61	-0.85	-0.75	-0.55	-0.03	-0.26	1.08	1.64	-0.45	-0.85	0.36	0.03	-1.65	2.14
Barbados	-0.01	-0.12	0.22	-0.38	-0.20	-0.25	-0.14	-0.27	-2.21	-2.02	-0.08	-0.20	0.37	-0.12	-0.46	-0.81	2.90	-1.89	-1.23	0.66
Belarus	-0.93	-0.45	-1.39	-2.03	-0.23	-0.26	-0.51	-1.02	1.94	-0.89	0.28	0.23	0.85	2.78	-0.31	0.01			-2.24	1.17
Belgium	-0.57	-0.55	-2.02	-2.33	-0.17	-0.47	-0.46	-1.06	-1.35	-1.46	0.36	0.78	0.94	0.76	-0.57	-0.68	0.36	0.33	-1.17	0.21
Bermuda	-0.49	-0.95	-1.38	-1.21	-0.48	-0.41	-0.55	-0.67	-0.67	-0.24	-0.64	-0.67	0.38	1.43	-0.34	-0.80			-0.59	0.68
Bolivia	-2.28	-1.96	-1.92	-1.56	-0.74	-0.48	-1.40	-1.13	-0.38	-0.20	-0.67	-0.32	3.10	2.41	-0.80	-0.84	2.26	1.51	-0.72	9.68
Bosnia and Herzegovi..	-1.49	-1.05	-9.63	-9.42	-0.33	-0.53	-1.50	-2.98	0.03	0.23	-0.39	-0.86	5.48	4.70	-1.26	-0.84			-2.89	5.75
Brunei	-0.85	-0.31	-1.31	-1.29	-0.13	-0.02	-0.37	-0.50	0.99	-0.94	-0.30	-0.21	4.37	4.05	-0.74	-0.65	1.66	0.76	-1.76	0.61
Bulgaria	-1.05	-1.03	-1.05	-1.39	-0.21	-0.23	-0.44	-0.68	0.51	-0.09	-0.92	0.15	0.67	0.58	-0.58	-0.11	1.33	1.64	-1.71	0.46
Canada	-0.19	-0.45	-1.28	-1.60	0.06	-0.17	-0.12	-0.59	-2.18	-1.82	0.03	-0.58	1.46	1.05	0.36	-0.10	1.53	2.03	-2.41	-0.05
Chile	-1.45	-1.48	-0.77	-1.30	0.00	-0.25	-0.22	-0.59	-0.85	-0.72	0.12	-0.06	3.36	4.01	-0.93	-1.14	2.47	2.37	-1.03	0.95
China	-2.16	-1.90	-1.98	-1.61	-1.26	-1.05	-1.50	-1.34	-1.85	-0.78	2.59	1.29	4.78	6.20	-0.09	0.01	-0.57	-0.65	-2.40	1.00
Costa Rica	-0.97	-0.87	-0.61	-0.26	-0.03	0.09	-0.20	-0.09	-1.21	-0.45	0.23	-0.35	3.41	2.28	-0.91	-0.88	2.82	1.72	-1.93	0.06
Croatia	-1.18	-0.87	-2.79	-3.00	-0.20	-0.47	-0.72	-1.42	0.32	-0.34	0.57	0.59	2.48	2.59	-0.89	-0.51	3.14	2.67	-2.45	0.17
Cuba	-1.05	-0.74	-2.00	-2.32	-0.92	-0.63	-1.08	-1.22	-2.09	-1.78	2.17	1.53	1.10	1.32	-0.80	-0.87	2.02	3.34	-1.47	0.32
Cyprus	-0.44	-0.30	-1.55	-1.64	0.07	0.01	-0.16	-0.56	2.52	1.23	-0.23	0.36	2.03	2.44	-0.23	-0.92	0.56	0.09	-2.80	0.60
Czech Republic	-0.72	-0.36	-1.03	-1.51	-0.15	-0.26	-0.34	-0.68	-0.10	-0.46	0.87	0.34	0.33	2.05	-0.14	-0.17	0.46	0.33	-2.39	1.41
Denmark	-0.61	-0.82	-1.75	-2.42	-0.21	-0.43	-0.40	-0.92	-2.42	-2.27	0.73	0.69	2.64	1.04	-0.14	-0.88	-0.08	-0.73	-2.34	0.20
Egypt	-1.99	-1.63	-1.56	-0.69	-0.42	-0.18	-0.98	-0.57	5.23	1.03	0.09	0.07	1.29	1.05	-0.89	-0.90	5.96	4.42	-1.45	2.93
Estonia	-0.55	-0.60	-2.33	-3.08	-0.49	-0.51	-0.85	-1.64	-0.91	-1.15	1.22	1.34	2.62	4.68	-0.26	-0.97	-0.09	0.05	-2.70	0.57
Finland	-0.71	-0.87	-1.52	-2.02	-0.13	-0.46	-0.32	-0.87	-1.55	-1.46	1.35	0.40	2.36	1.46	-0.80	-0.85	0.58	0.67	-1.78	0.18
France	-0.84	-1.03	-2.09	-2.34	-0.27	-0.50	-0.55	-1.11	-1.64	-1.55	0.50	0.19	2.43	1.54	-0.43	-1.03	2.07	1.77	-0.90	0.15
Germany	-0.52	-0.64	-1.89	-2.40	-0.20	-0.47	-0.42	-1.00	-0.83	-1.06	1.08	0.74	2.98	1.00	-0.38	-0.74	0.32	0.56	-2.39	0.61
Greece	-0.37	-0.53	-1.43	-1.67	-0.06	-0.13	-0.25	-0.67	-1.27	-1.07	0.12	-0.06	2.04	1.68	-0.11	-0.98	0.59	0.36	-2.09	0.67
Guam	-0.46	-0.20	0.61	-0.06	0.20	0.19	0.12	0.09	-1.41	-1.27	-0.48	-0.18	2.11	0.36	-0.71	-0.25			-1.30	0.69
Hungary	-0.54	-0.64	-1.48	-1.92	-0.42	-0.54	-0.59	-0.98	0.07	-0.60	-0.44	-0.32	0.29	1.02	-0.50	-0.87	2.37	1.87	-1.94	0.34
Iceland	-0.60	-0.70	-2.24	-2.58	-0.05	-0.42	-0.29	-1.00	-2.13	-1.98	0.01	-0.29	1.18	2.11	-0.03	-0.69	2.06	3.16	-3.24	0.28
Ireland	-0.83	-0.83	-1.44	-2.19	-0.10	-0.13	-0.28	-0.69	-0.99	-1.36	1.39	1.03	1.29	1.15	-0.99	-1.14	1.42	1.60	-1.96	0.51
Israel	-0.66	-0.48	-1.35	-1.42	-0.23	-0.28	-0.39	-0.58	-1.73	-0.77	0.39	0.21	2.56	3.11	-0.37	-0.27	0.70	0.66	-2.18	0.32
Italy	-0.85	-0.96	-1.57	-2.25	-0.25	-0.55	-0.43	-1.08	-0.92	-0.95	0.44	-0.86	1.18	0.85	-0.44	-0.75	2.03	2.29	-1.13	0.96
Japan	-0.32	-0.45	-1.58	-2.36	-0.01	-0.06	-0.17	-0.63	0.09	-0.96	-0.79	-1.15	1.30	1.79	0.08	0.05	0.03	0.12	-0.41	-0.28
Kuwait	-0.85	-0.56	-1.91	-1.67	-0.03	-0.07	-0.52	-0.85	0.22	1.10	0.05	-0.24	3.68	1.09	-0.83	-0.97	-0.10	-1.58	-3.64	2.02
Kyrgyzstan	-1.23	-1.61	-1.31	-1.99	-0.42	-0.66	-0.73	-1.24	6.31	-0.87	-0.02	-1.47	0.19	2.34	-0.06	0.12	0.41	0.52	-1.44	-0.97
Latvia	-1.11	-0.83	-2.36	-2.87	-0.29	-0.78	-0.80	-1.74	0.75	0.39	0.63	0.36	1.94	2.74	-0.79	-0.27	2.26	2.82	-2.14	1.28
Lebanon	-1.47	-1.35	-2.49	-2.24	-0.31	-0.45	-1.15	-1.54	2.78	2.81	-0.25	-0.12	1.68	3.01	-0.98	-1.02			-2.88	1.35
Lithuania	-0.34	-0.21	-1.80	-2.07	-0.28	0.00	-0.58	-0.89	2.73	-0.26	0.14	0.14	3.08	5.81	-0.32	-0.05	0.09	-0.12	-2.92	1.70
Luxembourg	-0.52	-0.95	-2.11	-2.51	-0.30	-0.70	-0.56	-1.28	-1.01	-0.97	-0.54	0.40	3.67	1.93	-0.20	-1.05	1.35	1.00	-2.32	0.23
Macedonia	-0.97	-0.80	-0.98	-1.08	-0.31	-0.13	-0.47	-0.46	3.28	1.70	0.19	0.09	2.63	2.56	-0.46	-0.48			-2.58	1.99
Malaysia	-1.49	-1.14	-1.26	-1.07	-0.10	-0.13	-0.57	-0.60	1.49	-0.32	-0.26	-0.13	4.42	5.75	-1.30	-1.15	3.43	3.25	-0.91	1.62
Maldives	-2.27	-1.91	-2.42	-2.12	-1.09	-0.85	-1.58	-1.30	0.30	2.36	-0.32	-0.20	9.88	8.70	-1.07	-1.29	-2.20	-2.58	-3.33	2.87
Malta	-0.43	-0.65	-0.78	-1.27	0.13	0.06	0.02	-0.27	-0.04	-0.71	-0.28	0.28	2.19	1.31	0.02	-0.48	-1.51	-2.20	1.01	0.81
Mauritius	-0.93	-0.67	-1.42	-0.38	-0.41	-0.18	-0.61	-0.28	3.70	1.40	-0.50	-0.17	4.84	5.48	-0.68	-0.89	3.28	2.10	-1.48	0.71
Moldova	-1.19	-1.26	-2.08	-2.28	-0.41	-0.56	-0.83	-1.34	1.49	0.40	0.66	0.61	4.62	3.27	-0.33	0.06	-0.02	-0.14	-2.53	0.29
Montenegro	-0.70	-0.41	-0.97	-1.02	-0.24	-0.26	-0.40	-0.50	5.53	5.29	0.31	0.20	0.52	1.43	-0.84	-0.33			-2.07	0.25
Morocco	-2.22	-1.83	-1.41	-1.16	-0.24	-0.16	-0.95	-0.75	-0.06	-1.20	0.02	-0.26	5.53	4.84	-0.67	-1.10	4.86	1.58	-2.03	2.13
Netherlands	-0.73	-0.52	-1.72	-2.26	-0.23	-0.27	-0.40	-0.70	-1.82	-1.70	1.98	-0.05	2.54	1.76	-1.09	-0.36	0.26	-0.17	-1.92	0.51
New Zealand	-0.75	-0.76	-1.88	-2.11	-0.12	-0.44	-0.42	-1.02	-1.51	-0.79	0.64	1.92	2.76	2.44	-0.61	-0.89	1.18	1.04	-1.26	0.36
Nicaragua	-2.05	-1.67	-2.24	-1.66	-0.34	-0.31	-0.91	-0.96	0.03	0.04	0.31	0.45	3.48	4.26	-1.40	-1.42	4.30	3.05	-1.53	2.16
Northern Mariana Isla..	-0.56	-0.44	-0.92	-1.17	-0.22	-0.19	-0.38	-0.47	0.05	-0.77	-0.13	-0.19	0.20	0.33	-0.34	-0.75			-3.01	0.79
Norway	-0.43	-0.37	-1.48	-2.13	-0.12	-0.40	-0.28	-0.85	-2.81	-2.61	0.40	-0.39	2.14	2.50	-0.02	-0.37	0.01	-0.67	-2.51	0.26
Palestine	-0.65	-0.99	-1.92	-0.64	-0.17	0.03	-0.50	-0.29	10.69	0.03	-0.08	-0.02	3.58	3.34	-0.04	-0.11			-0.23	0.74
Peru	-2.31	-2.31	-1.81	-1.80	-0.49	-0.56	-1.16	-1.28	-0.15	0.71	0.04	1.21	2.43	2.25	-0.77	-0.83	3.66	2.79	-0.67	4.36
Poland	-1.13	-0.91	-1.07	-1.57	-0.13	-0.15	-0.38	-0.71	-0.54	-1.05	0.94	0.98	3.56	3.74	-0.56	-0.46	0.47	0.43	-1.90	1.63
Portugal	-1.39	-2.94	-2.29	-2.98	-0.38	-0.78	-0.71	-1.78	0.15	-0.97	0.45	1.04	1.65	1.21	-1.05	-1.35	1.78	1.50	-2.47	0.47
Qatar	-1.56	-1.04	-0.65	-0.75	-0.41	-0.37	-0.58	-0.53	-3.61	1.36	-0.67	-0.82	4.36	1.04	-0.73	-1.15	1.86	-0.43	-2.65	2.34
Romania	-1.11	-0.90	-1.26	-1.43	-0.33	-0.30	-0.60	-0.75	0.16	-0.53	-0.29	-0.15	3.21	5.09	-0.49	-0.67	-0.07	-0.29	-1.11	1.90
Samoa	-0.92	-1.26	0.18	-0.57	-0.08	-0.54	-0.26	-0.66	-0.32	-0.97	-0.04	-0.13	1.63	0.60	-0.49	-0.50			-1.44	2.10
Saudi Arabia	-1.57	-1.34	-1.61	-0.97	-0.24	-0.04	-0.72	-0.55	-0.86	2.15	0.19	-0.15	3.01	3.99	-1.08	-2.12	7.34	4.33	-3.41	6.82
Serbia	-0.75	-0.59	-3.02	-3.17	-0.19	-0.30	-0.66	-1.21	0.10	0.40	0.39	0.32	0.99	1.32	-0.44	-0.50	3.55	2.83	-2.07	-1.26
Seychelles	-0.70	-0.50	-0.55	-0.62	-0.11	-0.36	-0.27	-0.45	0.96	0.11	-0.15	0.00	3.57	4.56	-0.79	-0.27			-0.44	0.90
Singapore	-1.05	-1.21	-1.03	-1.96	-0.48	-0.57	-0.58	-0.93	-1.50	-1.23	2.32	0.93	3.41	3.39	-0.69	-0.50	11.90	14.59	-2.53	0.61
Slovakia	-0.64	-0.63	-1.04	-1.38	-0.05	-0.15	-0.26	-0.59	-0.10	-1.23	0.08	0.34	1.04	2.96	-0.12	-0.37	0.80	0.58	-1.85	0.27
Slovenia	-0.39	-0.78	-1.69	-2.28	-0.31	-0.58	-0.56	-1.20	-0.60	-1.06	1.57	1.70	1.78	3.09	-0.10	-0.59	1.15	0.70	-2.76	0.14
South Korea	-1.42	-1.43	-2.40	-2.86	-0.23	-0.49	-0.67	-1.36	5.69	-1.78	-0.43	-0.25	0.98	3.07	-0.66	-0.78	0.49	0.34	-2.50	0.42
Spain	-1.06	-1.69	-2.04	-2.65	-0.24	-0.64	-0.52	-1.35	-1.41	-1.32	0.85	0.61	1.75	0.99	-0.58	-1.47	3.33	2.71	-0.58	0.41
Sri Lanka	-1.72	-1.89	-1.92	-2.46	-0.67	-1.16	-1.09	-1.72	-0.74	-0.04	-0.25	0.21	3.90	4.47	-1.36	-1.84	-1.32	-1.71	-2.56	2.98
Sweden	-0.25	-0.31	-1.34	-1.45	-0.01	0.09	-0.16	-0.27	-2.04	-1.76	-0.18	-0.03	3.19	2.15	0.04	-0.39	0.09	0.07	-1.71	-0.01
Switzerland	-0.47	-0.59	-2.19	-2.58	-0.41	-0.88	-0.63	-1.36	-1.96	-1.72	1.48	1.31	2.37	2.44	-0.22	-0.19	1.21	0.30	-2.38	0.09
Taiwan	-1.82	-1.74	-2.18	-2.64	-0.37	-0.59	-0.88	-1.49	-1.67	-1.83	0.24	0.15	2.19	2.69	-0.78	-1.34	0.86	3.34	-3.28	0.47
Tajikistan	-1.77	-1.32	-1.85	-1.86	-0.42	-0.09	-0.96	-0.84	-2.80	-1.91	0.07	0.11	0.28	1.65	-0.24	-0.21	-0.98	-2.01	-1.07	-0.16
Tunisia	-1.65	-1.02	-1.10	-0.65	-0.29	-0.18	-0.60	-0.41	-0.33	-0.51	0.54	-0.16	5.30	4.44	-1.26	-1.54	11.93	3.11	-2.39	0.65
Turkey	-2.00	-1.76	-1.84	-2.27	-0.49	-0.68	-0.90	-1.26	-1.44	-1.78	0.04	-0.92	1.21	3.60	-0.69	-0.88	8.29	4.05	-2.36	2.42
Turkmenistan	-1.74	-1.49	-1.78	-1.41	0.04	0.36	-0.61	-0.44	-0.23	-0.44	0.43	0.22	4.38	7.63	-1.05	-0.70			-1.40	2.82
Ukraine	-0.40	-0.16	-1.54	-2.07	-0.23	-0.31	-0.48	-1.01	0.35	-0.30	0.63	0.22	1.36	2.43	-0.24	0.36	-0.85	-0.52	-2.29	0.87
United Kingdom	-0.57	-0.64	-1.46	-1.98	-0.13	-0.36	-0.28	-0.74	-1.13	-1.00	0.18	0.29	2.06	1.63	-0.73	-0.60	8.71	6.34	-2.41	0.15
United States	-0.78	-1.12	-1.68	-1.79	0.23	0.10	-0.13	-0.62	-2.05	-1.81	-2.15	-0.52	2.71	1.72	-0.48	-0.39	0.28	0.37	-2.72	-0.63
Uruguay	-0.69	-0.10	-0.91	-0.19	0.00	0.33	-0.18	0.14	-1.43	-1.33	0.54	-0.03	3.20	5.23	-0.66	-0.84	7.27	-1.34	-0.69	0.90
Uzbekistan	-1.32	-0.76	-1.17	-0.04	-0.01	0.97	-0.48	0.42	-1.84	-0.84	-0.20	-0.39	7.11	6.17	-0.63	-0.75			-2.63	0.86
